# Characterization of the pathogenicity of strains of *Pseudomonas syringae* towards cherry and plum

**DOI:** 10.1111/ppa.12834

**Published:** 2018-02-14

**Authors:** M. T. Hulin, J. W. Mansfield, P. Brain, X. Xu, R. W. Jackson, R. J. Harrison

**Affiliations:** ^1^ NIAB EMR New Road East Malling ME19 6BJ UK; ^2^ School of Biological Sciences University of Reading Reading RG6 6AJ UK; ^3^ Faculty of Natural Sciences Imperial College London London SW7 2AZ UK

**Keywords:** non‐host, pathogenicity, *Pseudomonas*, resistance

## Abstract

Bacterial canker is a major disease of *Prunus avium* (cherry), *Prunus domestica* (plum) and other stone fruits. It is caused by pathovars within the *Pseudomonas syringae* species complex including *P. syringae* pv. *morsprunorum* (Psm) race 1 (R1), Psm race 2 (R2) and *P. syringae* pv. *syringae* (Pss). Psm R1 and Psm R2 were originally designated as the same pathovar; however, phylogenetic analysis revealed them to be distantly related, falling into phylogroups 3 and 1, respectively. This study characterized the pathogenicity of 18 newly genome‐sequenced *P. syringae* strains on cherry and plum, in the field and laboratory. The field experiment confirmed that the cherry cultivar Merton Glory exhibited a broad resistance to all clades. Psm R1 contained strains with differential specificity on cherry and plum. The ability of tractable laboratory‐based assays to reproduce assessments on whole trees was examined. Good correlations were achieved with assays using cut shoots or leaves, although only the cut shoot assay was able to reliably discriminate cultivar differences seen in the field. Measuring bacterial multiplication in detached leaves differentiated pathogens from nonpathogens and was therefore suitable for routine testing. In cherry leaves, symptom appearance discriminated Psm races from nonpathogens, which triggered a hypersensitive reaction. Pathogenic strains of Pss rapidly induced disease lesions in all tissues and exhibited a more necrotrophic lifestyle than hemibiotrophic Psm. This in‐depth study of pathogenic interactions, identification of host resistance and optimization of laboratory assays provides a framework for future genetic dissection of host–pathogen interactions in the canker disease.

## Introduction

*Pseudomonas syringae* is a globally important plant pathogen, and includes strains associated with plants and aquatic environments (Berge *et al*., 2014). Strains are divided into pathovars based on their ability to infect particular plant species; within pathovars, strains may be further distinguished into races that show specificity towards particular host cultivars (Joardar *et al*., 2005). *Pseudomonas syringae* is referred to as a species complex because of the high level of divergence between individual clades. Currently, nine genomospecies, based on DNA–DNA hybridization, and 13 phylogroups, based on multilocus sequence typing (MLST), have been described (Gardan *et al*., 1999; Parkinson *et al*., 2011).

Several distantly related pathovars of *P. syringae* are known to cause bacterial canker of *Prunus*. This genus of stone‐fruit trees includes economically important species such as cherry, plum, peach and apricot. Focusing on sweet cherry (*Prunus avium*), members within three distinct clades of *P. syringae* have been characterized as the main causal agents of canker. These are *P. syringae* pv. *syringae* (Pss), *P. syringae* pv. *morsprunorum* (Psm) race 1 (R1) and *P. syringae* pv. *morsprunorum* race 2 (Psm R2) (Bultreys & Kaluzna, 2010). More recently, Psm R1 has been classified as a member of the species *Pseudomonas amygdali*, whilst Psm R2 is a member of *Pseudomonas avellanae* (Bull *et al*., 2010). The two Psm races are specifically found only on *Prunus* species, whilst Pss strains are more variable and able to infect various plant species. Although now known to be distantly related, Psm R1 and Psm R2 were initially distinguished based on morphological features and aggressiveness towards particular cherry cultivars, so were described as ‘races’ of Psm (Freigoun & Crosse, 1975). They are able to infect throughout the year and cause brown/black lesions on all aerial plant organs, including fruit, leaves and blossom. The pathogens invade dormant woody tissues through leaf scars and wounds in winter, colonizing cambial tissue and causing cankers in spring. During the growing period, bacteria from the epiphytic population enter leaves causing brown/black spots that drop out to produce ‘shot‐hole’ symptoms.

Bacterial canker is an annual problem for the global cherry fruit industry and is particularly devastating in young orchards, where it has been reported to cause up to 75% loss of trees (Spotts *et al*., 2010). Chemical control for this disease is currently limited to spraying with copper‐based compounds, a treatment that has recently been restricted across Europe (Stone & Baker, 2010). Breeding for resistance is a desirable alternative method of control, but progress has been hampered by the complex nature of this disease (Farhadfar *et al*., 2016) and the need to prevent infection by three different clades of *P. syringae*.

An understanding of how the divergent clades of *P. syringae* cause bacterial canker is crucial to inform global breeding efforts. Field studies have revealed that the three clades coexist within orchards and with nonpathogenic pseudomonads on plant surfaces. To characterize pathogenicity, several laboratory‐ and field‐based assays have been developed (Crosse & Garrett, 1966; Vicente & Roberts, 2003). Gilbert *et al*. (2009) highlighted that improved assays are required to facilitate screening for host resistance. This study aimed to address this problem by comparing the aggressiveness of multiple strains on a range of cherry and plum cultivars in the field and in a series of more tractable, rapid laboratory‐based assays. These assays were critically assessed to determine their use in resistance screening and the classification of strain pathogenicity. By combining phylogenetic analysis with robust pathogenicity testing, this study provides a framework for future research focused on the genetic dissection of pathogenicity and disease resistance.

## Materials and methods

### Bacterial strains and inoculum

Strains of *P. syringae* (listed in Table 1) were grown on King's B agar (King *et al*., 1954) at 25 °C. For liquid culture, strains were grown in lysogeny broth (LB) at 25 °C with shaking at 150 rpm. Pss and Psm R1 included strains isolated from cherry and plum, whilst the Psm R2 strains all originated from cherry. A previously undescribed strain that did not belong to these clades (Ps 9643), which had been isolated from a symptomless plum leaf wash, was also included. Finally, an additional strain (RMA1), which is a pathogen of the perennial plant species *Aquilegia vulgaris* and that preliminary analysis had shown to be closely related to Psm R2, was included. Two additional nonhost strains, *P. syringae* pv. *phaseolicola* (Pph 1448A, pathogen of bean and related to Psm R1) and *P. syringae* pv. *avellanae* (Psv 631, pathogen on hazelnut and related to Psm R2), were also included. Bacterial inoculum was prepared from overnight LB cultures. These were centrifuged (3500 ***g***, 10 min) and resuspended in sterile 10 mm MgCl_2_. A spectrophotometer was used to measure optical density, where OD_600_ of 0.2 is *c*. 2 × 10^8^ CFU mL^−1^ (Debener *et al*., 1991). This method of concentration determination was validated for a set of strains at the start of this study (Table S1).

**Table 1:** *Pseudomonas* species used in this study with host of isolation and reference/source

Strain	Species	Clade	Plant host	*Prunus* host cultivar	Host tissue	Geographic origin	Isolator	Accession
R1‐5244	*Pseudomonas amygdali* pv. *morsprunorum*	Psm R1	*Prunus avium*	Unknown	Cankerous wood	Kent, UK	Crosse, 1960	MLEB00000000
R1‐5300	*P. amygdali* pv. *morsprunorum*	Psm R1	*Prunus domestica*	Victoria	Unknown	Kent, UK	Prunier, n.d.	MLEN00000000
R1‐9326	*P. amygdali* pv. *morsprunorum*	Psm R1	*P. domestica*	Victoria	Leaf wash	West Sussex, UK	Roberts, 2011	MLEO00000000
R1‐9629	*P. amygdali* pv. *morsprunorum*	Psm R1	*P. domestica*	Victoria	Leaf wash	Worcestershire, UK	Roberts, 2012	MLEP00000000
R1‐9646	*P. amygdali* pv. *morsprunorum*	Psm R1	*P. avium*	Stella	Leaf wash	Worcestershire, UK	Roberts, 2012	MLEE00000000
R1‐9657	*P. amygdali* pv. *morsprunorum*	Psm R1	*P. avium*	Kike‐Shidare	Leaf wash	West Sussex, UK	Roberts, 2012	MLEF00000000
R2‐5255	*Pseudomonas avellanae* pv. *morsprunorum*	Psm R2	*P. avium*	Napoleon	Unknown	Kent, UK	Prunier, n.d.	MLEC00000000
R2‐5260	*P. avellanae* pv. *morsprunorum*	Psm R2	*P. avium*	Roundel	Unknown	Kent, UK	Garrett, n.d.	MLEG00000000
R2‐leaf	*P. avellanae* pv. *morsprunorum*	Psm R2	*P. avium*	Napoleon	Leaf lesion	Kent, UK	Hulin, 2014	MLEH00000000
R2‐sc214	*P. avellanae* pv. *morsprunorum*	Psm R2	*P. avium*	Wild cherry	Leaf lesion	Oxfordshire, UK	Roberts, 1983	MLEI00000000
Pss 9097	*Pseudomonas syringae* pv. *syringae*	Pss	*P. avium*	Unknown	Cankerous wood	Wawickshire, UK	Roberts, 2010	MLEJ00000000
Pss 9293	*P. syringae* pv. *syringae*	Pss	*P. domestica*	Victoria	Leaf wash	Worcestershire, UK	Roberts, 2011	MLEQ00000000
Pss 9630	*P. syringae* pv. *syringae*	Pss	*P. domestica*	Victoria	Leaf wash	Worcestershire, UK	Roberts, 2012	MLER00000000
Pss 9644	*P. syringae* pv. *syringae*	Pss	*P. avium*	Stella	Leaf wash	Worcestershire, UK	Roberts, 2012	MLEK00000000
Pss 9654	*P. syringae* pv. *syringae*	Pss	*P. domestica*	Victoria	Leaf wash	West Sussex, UK	Roberts, 2012	MLES00000000
Pss 9656	*P. syringae* pv. *syringae*	Pss	*P. avium*	Kiku‐Shidare	Leaf wash	West Sussex, UK	Roberts, 2012	MLEM00000000
Pss 9659	*P. syringae* pv. *syringae*	Pss	*P. avium*	Kiku‐Shidare	Leaf wash	West Sussex, UK	Roberts, 2012	MLEL00000000
Ps 9643	*P. syringae*		*P. domestica*	Victoria	Leaf wash	Worcestershire, UK	Roberts, 2012	MLET00000000
RMA1	*P. syringae*		*Aquilegia vulgaris*	Winky	Leaf lesion	West Sussex, UK	Jackson, 2008	MLEU00000000
Psv 631	*P. syringae* pv. *avellanae*		*Corylus avellana*			Greece	1976	AKBS00000000
Pph 1448A	*P. syringae* pv. *phaseolicola*		*Phaseolus vulgaris*			Ethiopia	Teverson, 1965	CP000058

Strains sequenced in this study are listed first, followed by the out‐group strains Psv 631 and Pph 1448A included in pathogenicity tests. Newly classified species names are given in this table (Bull *et al.,* 2010). However, as the strains are all part of the *P. syringae* species complex they are referred to as *P. syringae* for the rest of the article. GenBank accessions are also listed. Details of further out‐groups used solely for phylogenetic analysis can be found in Table S2.

### Genome sequencing, assembly and phylogenetics

The complete genomes of 18 *P. syringae* strains were sequenced using MiSeq v. 3 (Illumina) with 300 bp paired‐end reads. DNA was extracted using the Puregene Yeast/Bact kit (QIAGEN). DNA libraries were prepared by fragmenting the DNA using a sonicating waterbath for 30 s. DNA was then size‐selected by gel electrophoresis to obtain fragments of 400–700 bp using the Zymogen Gel Extraction kit (Zymo Research). Libraries were created using the NextFlex Rapid‐DNA Sequencing kit (Bioo Scientific), and quality checked using the Fragment Analyzer (Advanced Analytical) and Qubit (Life Technologies). Barcodes were multiplexed to allow pooling of multiple samples. Raw data for each genome were checked and trimmed using fastqc‐mcf (Andrews, 2010). Each genome was then assembled using SPades v. 3.7.0 (Bankevich *et al*., 2012) and annotated using the ‘Rapid annotation using subsystem technology’ online server (RAST) (Aziz *et al*., 2008). Summary statistics were generated using quast (Gurevich *et al*., 2013). The genomes were uploaded to GenBank under the BioProject no. PRJNA345357.

A phylogenetic tree was built including the newly sequenced strains (Table 1) with other genome sequences available on NCBI (Table S2). The nucleotide sequences of seven housekeeping genes (*acnB*,* fruK*,* gapA*,* gltA*,* gyrB*,* pgi* and *rpoD*) were extracted from all genomes and individually aligned using geneious v. 7.1.9 (Kearse *et al*., 2012). The alignments were concatenated and trimmed to produce an overall alignment of 9393 bp. A Bayesian phylogeny was created using the geneious plug‐in of mrbayes (Huelsenbeck & Ronquist, 2001). The GTR gamma model of evolution was used with a burn‐in length of 100 000 and subsampling frequency of 200.

### Plant material

All *Prunus* stock material was propagated at NIAB EMR, UK. For whole‐tree inoculations performed in the glasshouse or field, 1‐year‐old grafted trees were used. Cherry cultivars Merton Glory, Napoleon, Roundel and Van were grafted on the rootstock Gisela 5, whilst plum cultivars Marjorie's Seedling and Victoria were grafted on St Julian A. Merton Glory is reported to show some resistance to canker (American Pomological Society, 1966), Freigoun & Crosse (1975) considered Napoleon and Roundel to display quantitative race‐specific differences, with Napoleon being more susceptible to Psm R1 than to Psm R2 (and vice versa in Roundel). The cultivar Van is reported to be universally susceptible (Long & Olsen, 2013). For plum, Victoria is reported to be highly susceptible and Marjorie's Seedling more resistant (Royal Horticultural Society, 2017). For detached leaf assays, 1–2‐week‐old, fully expanded leaves were obtained from glasshouse‐grown trees. Immature green cherry fruits were obtained from mature field‐grown trees.

### Characterizing pathogenicity on cherry and plum trees

#### Whole‐tree glasshouse experiment

Inoculations were conducted in February 2015 on cherry cv. Van. Bacterial suspensions of 2 × 10^7^ CFU mL^−1^ were used for inoculations through wounds. A sterile scalpel was used to cut a shallow wound into the trunk of the tree and 200 μL of inoculum was pipetted into the wound. The inoculation sites were covered with Parafilm and duct tape. Five inoculations were performed on the same tree, with four buds between inoculations. The glasshouse experiment was assessed 2 months after inoculation. All 21 strains (Table 1) and one 10 mm MgCl_2_ negative control were assessed, with five replicates of each strain. The treatments were randomized across 22 trees using an unbalanced, incomplete block design generated with R software (R Core Team, 2012). Bark was stripped back and a disease score determined as: 1, no symptoms; 2, limited browning; 3, brown/black symptoms and gumming; and 4, brown/black symptoms, gumming and spreading of lesion from the site of inoculation.

#### Field experiment using leaf scars and wounds

Trees were inoculated through either wounds or leaf scars. Inoculations were performed in October 2015 on the four cherry cultivars (Merton Glory, Napoleon, Roundel and Van) and the two plum cultivars (Marjorie's Seedling and Victoria). The field experiment was conducted using 1‐year‐old grafted trees with no symptoms of bacterial canker or other diseases. The plot was located at lat 51.279, long 0.447. Climatic conditions, including temperature, rainfall, sunshine and relative humidity can be found in Table S3. The soil type was luvisol (http://mapapps2.bgs.ac.uk/). Frosts (<0 °C) were recorded in all months apart from December and June.

The aggressiveness of eight strains (R1‐5244, R1‐5300, R2‐leaf, Pss 9097, Pss 9293, Ps 9643, RMA1 and Pph 1448A) was assessed using the two inoculation methods. To reduce the number of trees required, the eight strains were divided across two trees, with each tree also having one negative control (10 mm MgCl_2_). This meant that two adjacent trees comprised one experimental unit of all strains and controls inoculated on the same cultivar using one inoculation method. A balanced incomplete design was used to randomize strain positions onto the two trees. A balanced complete design was then used to randomize the different cultivars and inoculation methods within 10 blocks in the field. Each block contained 24 trees (16 cherry and 8 plum), and the total experiment involved 240 trees.

To inoculate leaf scars, the leaf was removed and 10 μL of bacterial suspension was pipetted on the exposed scar. Wound inoculations were performed as in the glasshouse experiment. The inoculation sites were covered with Parafilm and duct tape. Five inoculations were performed on each tree, with four buds between inoculations. Trees were left for 213 days before assessment at the start of June 2016. Disease was assessed using the same disease scale as in the glasshouse experiment and also by measuring the length of brown/black symptoms produced.

### Laboratory‐based pathogenicity assays

#### Cut shoot inoculations

Eight bacterial strains were inoculated onto the four cherry and two plum cultivars. Inoculum was prepared at a concentration of 2 × 10^7^ CFU mL^−1^ and inoculated onto dormant 1‐year‐old shoots as in a previous study (Li *et al*., 2015).

#### Inoculation of detached immature cherry fruits

A stab‐inoculation method based on Moragrega *et al*. (2003) was used. Fruits were surface sterilized in 0.5% hypochlorite for 5 min and rinsed thoroughly in distilled water. Bacteria were then scraped from 5‐day‐old cultures on King's B agar plates using a 24‐gauge needle and stabbed into fruits that were placed in transparent boxes, lined with moist tissue paper to maintain a high humidity, and incubated at 22 °C (16 h light, 8 h dark) for observation over time with final assessment 10 days post‐inoculation (dpi). This assay was performed for all strains used in this study and for cherry‐pathogenic strains on different cherry cultivars. Two independent experiments were performed on each cultivar.

#### Inoculation of detached leaves

Inoculum concentration varied from 2 × 10^6^ CFU mL^−1^ (for population counts) to 2 × 10^8^ CFU mL^−1^ (symptoms, including detection of the hypersensitive response, HR). Freshly picked, 1–2‐week‐old leaves were infiltrated with bacterial suspension (approximately 50 μL at each site) from the abaxial surface using a blunt‐ended 1 mL syringe. Leaves were then placed in plastic trays on top of a 10 mm layer of water agar (10 g agar L^−1^) covered in damp paper towel. The tray was sealed inside a transparent bag and incubated at 22 °C (16 h light, 8 h dark). The leaves were left for a maximum of 10 days before assessment. At least three leaves from different plants were inoculated with each strain.

To measure bacterial multiplication, leaf discs were excised from inoculation sites using a sterile cork borer (0.5 cm) and homogenized in 1 mL of 10 mm MgCl_2_. A dilution series was plated out to determine bacterial concentration (CFU mL^−1^). Each concentration was plated out three times (pseudoreplicates). Overall, for each bacterial strain studied there were three replicate leaf inoculations and three pseudoreplicates to measure the concentration of each. Population growth was measured for all strains in this study on cherry and for reference strains on cherry and plum as well as on different cherry cultivars. Symptom scoring was performed for reference strains on cherry and plum. Two independent experiments were performed for the population and symptom scoring assays of a subset of strains inoculated on cherry and plum and the population growth experiment on different cherry cultivars.

### Statistical analysis

R software (R Core Team, 2012) was used for all statistical analyses as described in detail in Text S1. All ANOVA tables are also presented in the supplementary data.

## Results

### Genome sequencing, assembly and phylogenetics

**Figure 1:**
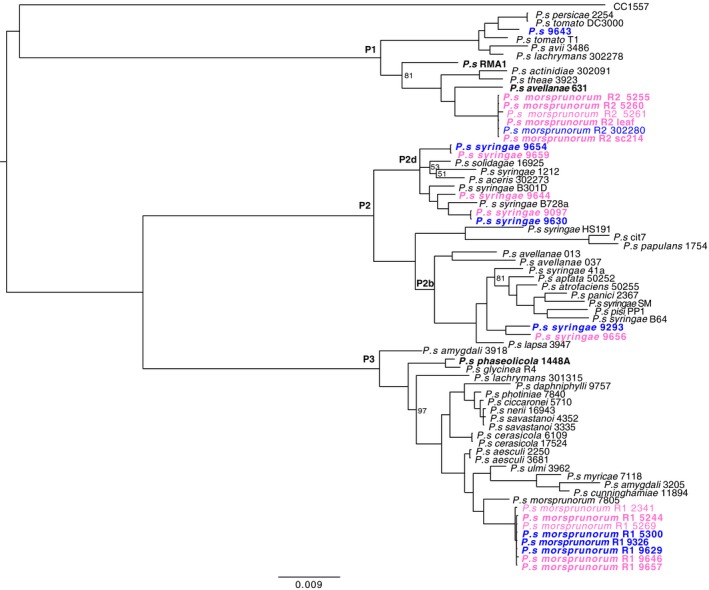
Bayesian phylogenetic tree of *Pseudomonas syringae*. The phylogeny was constructed using a concatenated alignment of seven genes (*acnB*,* fruK*,* gapA*,* gltA*,* gyrB*,* pgi* and *rpoD*). A subset of strains from the bacterial canker‐causing clades *P. syringae* pv. *syringae*,* P. syringae* pv. *morsprunorum* race 1 (R1) and *P. syringae* pv. *morsprunorum* race 2 (R2) was selected for analysis. Strains isolated from cherry are in pink, whilst those from plum are in blue. Phylogroups are labelled P1 to P3. The pathogenicity of strains in bold was tested in this study. Scale bar shows substitutions per site. Bootstrap support values are >99% unless otherwise indicated.

To analyse the characteristics of cherry canker disease, this study sought to examine a range of strains isolated from different *Prunus* species spread across UK geographical regions. The genomes of 18 strains were sequenced, along with a closely related strain, RMA1, from *A. vulgaris*. All genomes were sequenced with an average coverage of 200× and assembled into 140 contigs on average. Table S4 lists the genome assembly and annotation statistics. The *P. syringae* phylogeny based on concatenated MLST loci is presented in Figure 1, with strains isolated from cherry and plum highlighted. Psm R1, Psm R2 and Pss were found within phylogroups 3, 1 and 2, respectively, and grouped with strains already identified as these pathovars. Psm R1 and Psm R2 fell into discrete monophyletic clades, with individual strains being very closely related. By contrast, Pss strains exhibited considerable diversity. Strains isolated from cherry and plum did not form distinct host‐specific clusters in any of the pathogenic clades. The strain isolated from a plum leaf wash, Ps 9643, was closely related to *P. syringae* pv. *persicae* 2254 (a pathogen of *Prunus persicae*) and *P. syringae* pv. *tomato* DC3000. The strain from *A. vulgaris*, RMA1, was an out‐group to the clade containing strains of Psm R2, and *P. syringae* pv. *actinidiae*,* P. syringae* pv. *avellanae* and *P. syringae* pv. *theae*, which infect kiwifruit, hazelnut and tea, respectively.

### Characterizing pathogenicity on cherry and plum trees

#### Whole‐tree glasshouse experiment

**Figure 2:**
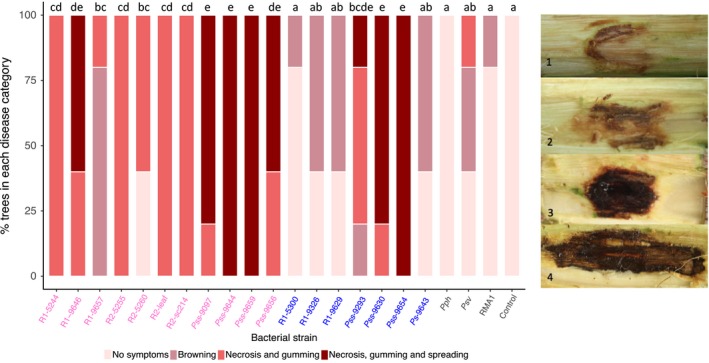
Percentage of inoculations in each disease score category after wound inoculation of cherry cv. Van with strains of *Pseudomonas syringae*:* P. syringae* pv. *syringae* (Pss), *P. syringae* pv. *morsprunorum* (Psm) race 1 (R1) and Psm race 2 (R2), selected out‐group nonpathogen strains (*P. syringae* pv*. phaseolicola* 1448A, Pph; *P. syringae* pv. *avellanae* 631, Psv; *P. syringae* RMA1) and a 10 mm MgCl_2_ control. Data presented are the percentage of replicates (*n *= 5) for each strain in each disease category. This complete experiment was performed once. Disease symptoms were scored on a ordinal scale as illustrated: 1, no symptoms; 2, limited browning; 3, brown/black symptoms and gumming; 4, brown/black symptoms, gumming and spread from site of inoculation. Strains isolated from cherry are labelled in pink, whilst those from plum are in blue. Statistical Tukey‐HSD (*P *= 0.05; confidence level: 0.95) groupings of bacterial strains determined by a proportional odds model (POM) analysis are presented above the bar. Full statistical analysis can be found in Table S5.

To determine the fundamental ability of all newly sequenced strains included in this study to cause bacterial canker on cherry, a whole‐tree wound inoculation experiment was performed. Strains exhibited a wide range of aggressiveness on cherry (Fig. 2). The non‐*Prunus* strains (Pph 1448A, Psv 631 and RMA1) and 10 mm MgCl_2_ inoculation caused very limited browning and callusing associated with a wound response. By contrast, strains were classified as pathogenic if they caused symptoms significantly different from the control inoculations. Pathogens caused brown/black discolouration and gumming symptoms that sometimes spread from the inoculation site. There was clear variation between members of the different *Prunus*‐infecting clades. Within Psm R1, strains isolated from plum (R1‐5300, R1‐9326 and R1‐9629) caused symptoms that were not significantly different from those associated with the control. Two cherry strains (R1‐5244 and R1‐9646) caused gumming and brown/black symptoms, whereas R1‐9657 showed reduced aggressiveness. Most strains of Psm R2 were recorded as pathogenic; however, R2‐5260 was the least aggressive. Apart from one strain (Pss 9293), all Pss strains (isolated from cherry or plum) were highly pathogenic, with symptoms typically spreading from the site of inoculation. The strain Ps 9643, isolated from a plum leaf and not closely related to the other canker‐causing pathogens (Fig. 1), did not cause significantly more symptoms than the non‐*Prunus* strains and the control, so was considered to be nonpathogenic on cherry.

#### Field experiment using leaf scar and wound inoculation

A set of strains with contrasting pathogenicity and origin was chosen for screening under field conditions, using leaf scar and wound inoculations on cherry and plum cultivars. Based on results from the glasshouse experiment, cherry pathogens (R1‐5244, R2‐leaf, Pss 9097 and Pss 9293) and those that were considered nonpathogens on cherry (R1‐5300, Ps 9643, Pph 1448A and RMA1) were included.

**Figure 3:**
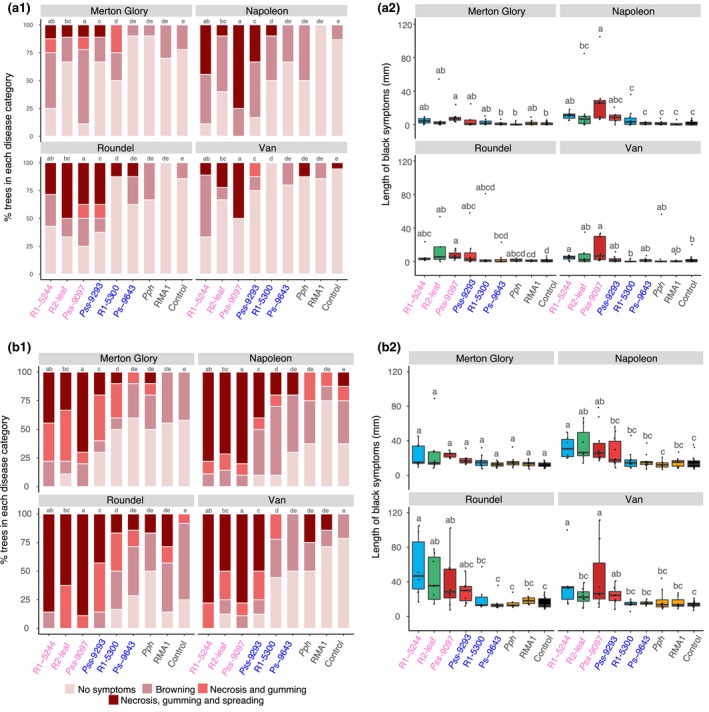
Field inoculation of cherry cultivars with selected strains of *Pseudmonas syringae*. Data presented are the disease score and length of disease symptoms observed 7 months after inoculation. (a) Leaf scar inoculation; (b) wound inoculation. The plots are ordered by host of isolation (cherry, plum, other plant species and 10 mm MgCl_2_ control) and *P. syringae* clade (*P. syringae* pv. *morsprunorum* (Psm) race 1 (R1), Psm race 2 (R2), *P. syringae* pv. *syringae* (Pss), *P. syringae* pv*. phaseolicola* 1448A (Pph), *P. syringae* RMA1). (1) Percentage of replicates for each strain (*n *= 10) in each disease score category (colour‐coded as in Figure 2). (2) Boxplot of length of symptoms associated with each strain on the four cultivars. Boxplots are colour‐coded for each strain based on clade R1 (blue), R2 (green), Pss (red), nonpathogens (orange) and 10 mm MgCl_2_ control (black). All data points (*n *= 10) are presented. This complete experiment was performed once. Strains isolated from cherry are labelled in pink, whilst those from plum are in blue. For disease score a proportional odds model (POM) analysis indicated that there was a significant difference between inoculation method (*P *< 0.01, d.f. = 1), between *P. syringae* strains (*P *< 0.01, d.f. = 8) and between cultivars (*P *< 0.01, d.f. = 3). As there was no interaction between strain and cultivar in the POM analysis, the post hoc groupings were the same across the plots. For symptom length, REML analysis indicated there were significant differences between strains and cultivars for both the leaf scar and wound experiments (*P *< 0.01, d.f. = 8 and *P *< 0.01, d.f. = 3 respectively). Tukey‐HSD (*P *= 0.05, confidence level: 0.95) groups are presented above each strain for each cultivar. Full statistical analysis can be found in Tables S6 and S7 (POM disease score analysis) and Tables S8 and S9 (REML symptom length analysis).

In cherry, data for both disease score (on an ordered categorical scale) and lesion length (mm) are presented in Figure 3. Inoculation through leaf scars (Fig. 3a) caused significantly less symptoms than through wounds (Fig. 3b), with few sites developing spreading lesions (Table S6). With both inoculation methods, the strains previously classified as pathogens (R1‐5244, R2‐leaf, Pss 9097 and Pss 9293) caused brown/black symptoms and gumming (score ≥3), and in some cases lesions spread extensively beyond the inoculation site. Strains of Psm R1 and Psm R2 as well as Pss were able to spread from sites of inoculation, whereas in the glasshouse experiment Psm R1 and Psm R2 rarely spread. This difference was probably due to differing environmental conditions and the extension of this experiment from 2 to 7 months. Strains designated as nonpathogens in the glasshouse assessment generally induced limited browning (scores 1–2), with disease score profiles similar to the control. Within this group, the plum strain R1‐5300 was not significantly different from the non‐*Prunus* strains. In the field, contamination by wild pseudomonads may have occurred, and this may explain why 6% of the 10 mm MgCl_2_ controls scored ≥3.

Because of the low frequency of establishment of spreading lesions, data for lesion lengths appeared highly variable (Fig. 3a2,b2). Nevertheless, there was clear restriction of lesions after scar inoculation for all cultivars and in both inoculation types, the cultivar Merton Glory appeared most resistant to canker. This cultivar had the lowest mean symptom length in wound inoculations and lowest overall symptom score following both inoculation methods. However, due to the large variability in lesion length, these differences were not always significant (Tables S7, S8 & S9). Apart from in cv. Van, where the R2‐leaf strain was associated with significantly reduced lesion length compared to symptoms caused by R1‐5244 and Pss 9097 (Fig. 3b2), no other clear cultivar‐specific differences in susceptibility to the different pathogenic strains emerged.

**Figure 4:**
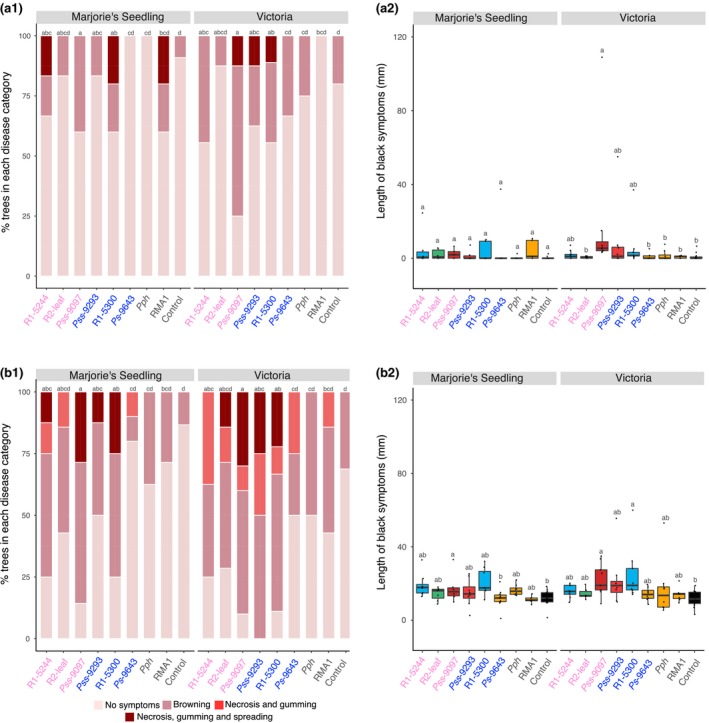
Field inoculations of different plum cultivars with selected strains of *Pseudomonas syringae*. Data are presented exactly as in Figure 3; strains are *P. syringae* pv. *morsprunorum* (Psm) race 1 (R1), Psm race 2 (R2), *P. syringae* pv. *syringae* (Pss), *P. syringae* pv*. phaseolicola* 1448A (Pph) and *P. syringae* RMA1. Strains isolated from cherry are labelled in pink, whilst those from plum are in blue. For disease score, proportional odds model (POM) analysis indicated there were significant differences between inoculation method (*P *< 0.01, d.f. = 1), strains (*P *< 0.01, d.f. = 8) and cultivars (*P *< 0.01, d.f. = 1). For symptom length, REML analysis indicated there were significant differences between strains in both inoculation experiments (*P *< 0.01, d.f. = 8) but not between host cultivars (*P *= 0.20, d.f. = 1 for leaf scar, *P *= 0.35, d.f. = 1 for wound). Tukey‐HSD (*P *= 0.05, confidence level: 0.95) groups are presented above each strain for each cultivar. Full statistical analysis can be found in Tables S10 and S11 (POM disease score analysis) and Tables S12 and S13 (REML symptom length analysis).

In plum, symptoms produced were similar to those on cherry, with blackening and gumming being indicative of disease (Fig. 4). Strains with confirmed pathogenicity against cherry were able to spread; however, in comparison to the cherry inoculations, the R1‐5300 plum strain was at least as aggressive as R1‐5244. The strain Ps 9643 did not cause symptom lengths significantly different from controls. Inoculation through wounds rather than scars caused significantly greater symptom scores (Table S10). There was no significant difference in lesion length between cultivars; however, the plum cultivar Marjorie's Seedling did not appear to be susceptible to leaf scar infection, as no strain was associated with a symptom length significantly different from the control.

### Laboratory‐based pathogenicity assays

#### Cut shoot inoculations

**Figure 5:**
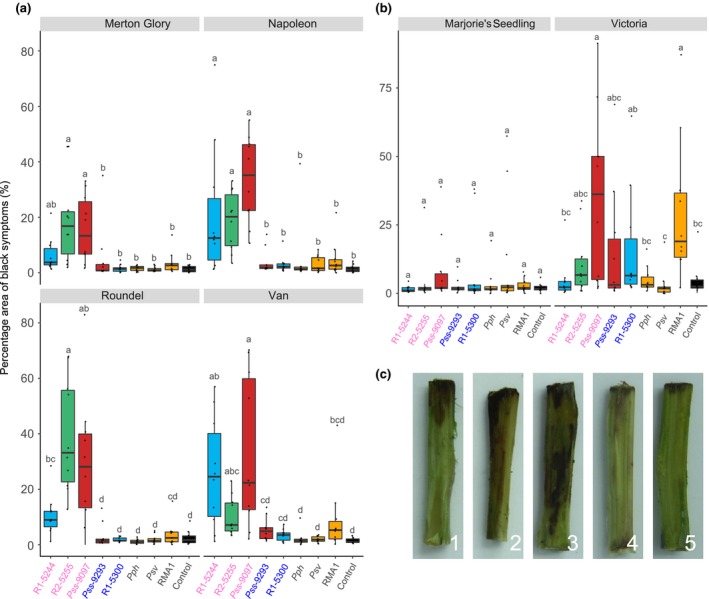
Lesion development on cut shoots of cherry and plum cultivars following inoculation with *Pseudomonas syringae*. The plots are ordered by host of isolation (cherry, plum, other plant species and the 10 mm MgCl_2_ control). (a) Boxplot of percentage area of black/brown discolouration in the top 30 mm associated with different *P. syringae* strains on four cherry cultivars (a) and two plum cultivars (b). All data points for each treatment (*n *= 10) are presented. This experiment was performed once. The bar chart is colour‐coded based on clade: *P. syringae* pv. *morsprunorum* (Psm) race 1 (R1), blue; Psm race 2 (R2), green; *P. syringae* pv. *syringae* (Pss), red; nonpathogens (*P. syringae* pv*. phaseolicola* 1448A, Pph; *P. syringae* pv. *avellanae* 631, Psv; *P. syringae* RMA1), orange; control, black. (c) Representative images of the symptoms on shoots of cherry cv. Napoleon inoculated with Pss 9097 (1–4) or the 10 mm MgCl_2_ control (5). Strains isolated from cherry are labelled in pink, whilst those from plum are in blue. An ANOVA revealed there were significant differences between bacterial strains (*P *< 0.001, d.f. = 8), no significant difference between the susceptibility of the two *Prunus* species (*P *= 0.57, d.f. = 1) and there was a significant interaction between *Prunus* species and *P. syringae* strain (*P *< 0.01, d.f. = 8) as well as interactions between strain and individual cultivars (*P *< 0.01, d.f. = 36). Tukey‐HSD (*P *= 0.05; confidence level: 0.95) significance groups for the different strains for each separate cultivar are presented above each boxplot. Full statistical analysis can be found in Table S14.

Cut shoot inoculations were performed on both cherry and plum (Fig. 5). Strains exhibited host specificity towards the two *Prunus* species. Focusing on cherry, R1‐5244, R2‐5255 and Pss 9097 were able to cause distinctive brown/black symptoms. In general, Psm R1 caused more symptoms on Van than on Roundel and vice versa with Psm R2, although the differences on Van were not statistically significant. The cut shoot test also confirmed that Merton Glory showed some resistance compared to the other cultivars, with disease symptoms significantly reduced compared to Napoleon and Van (Table S14). On plum, the severity of symptoms caused by all strains that were pathogenic in the field experiment was much greater on cultivar Victoria than on Marjorie's Seedling. As observed in the field experiment, R1‐5300 was able to cause brown/black disease symptoms on plum whereas it had failed on cherry. Unexpectedly, on plum cv. Victoria, the *A. vulgaris* pathogen RMA1 caused considerable disease symptoms.

#### Inoculation of detached immature cherry fruits

**Figure 6:**
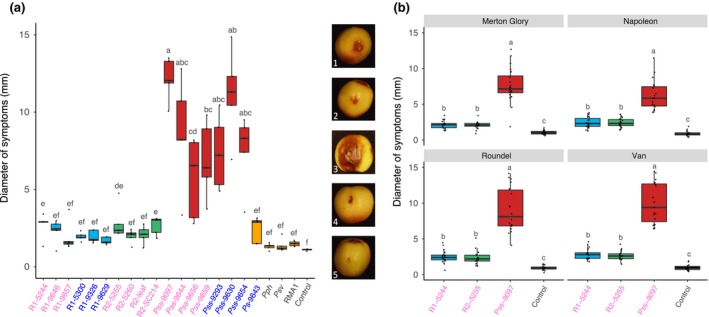
Boxplot to show diameter of brown/black lesions caused by different *Pseudomonas syringae* strains on immature cherry fruits 10 days post‐inoculation. (a) All strains used in this study. Strains isolated from cherry are labelled in pink, whilst those from plum are in blue. The boxplot is colour‐coded, *Pseudomonas syringae* pv. *morsprunorum* (Psm) race 1 (R1), blue; Psm race 2 (R2), green; *P. syringae* pv. *syringae* (Pss), red; nonpathogens, orange; control, black. All data points for each treatment (*n *= 5) are shown. This complete experiment was performed once. Representative images are presented: 1, Psm R1; 2, Psm R2; 3, Pss; 4, nonpathogens; 5, 10 mm MgCl_2_ control. An ANOVA revealed significant differences between strains (*P *< 0.01, d.f. = 21). Tukey‐HSD (*P *= 0.05, confidence level: 0.95) significance groups are presented above each bar. (b) Boxplot of diameter of brown/black symptoms caused by cherry pathogens on four cherry cultivars using immature cherry fruits. Data presented are all values (*n *= 20) per treatment of two independent experiments. An ANOVA revealed significant differences between strains (*P *< 0.01, d.f. = 3), cultivars (*P *< 0.01, d.f. = 3) and a significant interaction (*P *< 0.01, d.f. = 9). Tukey‐HSD (*P *= 0.05, confidence level: 0.95) significance groups for the different strains for each separate cultivar are presented above each boxplot. Full statistical analysis can be found in Tables S15 and S16.

The different clades that infect *Prunus* produced remarkably different symptoms in cherry fruits. Strains of Pss produced large black lesions within 2 days, and these expanded over time. By contrast, both Psm R1 and Psm R2 produced water‐soaked lesions within 2 days, and these did not increase in size. Most of the strains that had failed to induce symptoms on trees caused limited browning. Qualitative assessment based on symptom appearance allowed differentiation between pathogens and nonpathogens (Figs S1–S4); however, Ps 9643 caused water‐soaking similar to that observed with the pathogenic Psm races. Measurements of lesion diameter caused by all *P. syringae* strains (Fig. 6) confirmed significant differences between strains. However, diameters of the Psm‐induced water‐soaked lesions were not always greater than the brown lesions formed by non‐*Prunus* strains. There were limited differences between cultivars, and the resistance seen for Merton Glory in the field was not immediately apparent, with lesion diameters not significantly different from those recorded in cvs Napoleon and Roundel.

#### Inoculation of detached leaves

**Figure 7:**
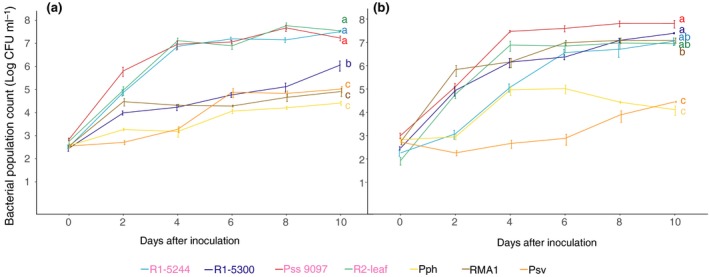
Population counts of different strains over time on cherry cv. Van (a) and plum cv. Victoria (b) leaves. Strains isolated from cherry are labelled in pink, whilst those from plum are in blue. Line colours for each strain are presented in the key and are colour‐coded by clade: *Pseudomonas syringae* pv. *morsprunorum* (Psm) race 1 (R1), blue; Psm race 2 (R2), green; *P. syringae* pv. *syringae* (Pss), red; nonpathogens, orange. Population counts are log CFU mL ^−1^. Data presented are the mean values (*n *= 9), with error bars showing standard error above and below the mean. This complete experiment was performed once. An ANOVA revealed significant differences between strains (*P *< 0.01, d.f. = 8). Tukey‐HSD (*P *= 0.05, confidence level: 0.95) significance groups for the different strains (based on day‐10 populations) are presented. Full statistical analysis can be found in Table S17.

A pilot experiment determined the best method for leaf inoculation was by blunt syringe‐infiltration (Fig. S5). Following inoculation at low concentration, the cherry pathogens identified from whole‐tree inoculations (R1‐5244, R2‐leaf and Pss 9097) exceeded levels of 10^6^ CFU mL^−1^ within 4 days (Fig. 7a) and caused disease symptoms after 7–10 dpi. The previously designated nonpathogens to cherry, Pph 1448A, Psv 631, RMA1, and Psm R1‐5300, isolated from plum, failed to reach 10^6^ CFU mL^−1^ or induce symptoms even after 10 days *in planta* (Fig. 7). On plum, the pathogens (including R1‐5300) also exceeded 10^6^ CFU mL^−1^ after 4 days. In contrast to its behaviour in cherry, RMA1 was capable of multiplication in plum leaves (Fig. 7b), potentially reflecting its ability to cause symptoms in cut shoots (Fig. 5).

**Figure 8:**
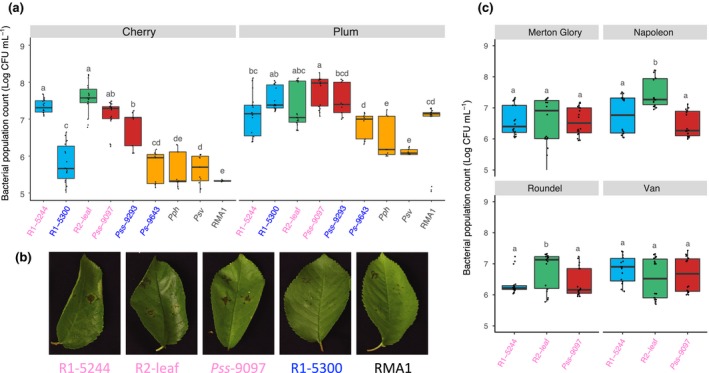
Pathogenicity of different strains, assessed by population counts on cherry and plum leaves and on different cherry cultivars. (a) Boxplots of day‐10 population counts on cherry cv. Van and plum cv. Victoria. Strains isolated from cherry are labelled in pink, whilst those from plum are in blue. Boxplots are colour‐coded by clade: *Pseudomonas syringae* pv. *morsprunorum* (Psm) race 1 (R1), blue; Psm race 2 (R2), green; *P. syringae* pv. *syringae* (Pss), red; nonpathogens, orange. Population counts are log CFU mL ^−1^. Data presented are all the values from two independent experiments (*n *= 18), although Pss 9293 and Ps 9643 were assessed only once. REML analysis for both cherry and plum revealed significant differences between strains (*P *< 0.01, d.f. = 8). Tukey‐HSD (*P *= 0.05, confidence level: 0.95) significance groups for the different strains are presented. (b) Representative images of symptom development at inoculation sites on cherry cv. Van leaves 10 dpi. Black pen dots mark the edge of inoculations. The pathogens Psm R1‐5244, Psm R2‐leaf and Pss 9097 caused brown/black disease lesions with some yellowing around the lesion edge; nonpathogens Psm R1‐5300 and RMA1 failed to produce any symptoms. (c) Boxplot of day‐10 population counts of three pathogenic *P. syringae* strains on different cherry cultivars. Data presented are all the values for each treatment of two independent experiments (*n *= 18). Tukey‐HSD (*P *= 0.05, confidence level: 0.95) significance groups for the different strains on each separate cultivar are presented. An ANOVA revealed significant differences between strains (*P *< 0.01, d.f. = 2), cultivars (*P *< 0.01, d.f. = 3) and a significant interaction (*P *< 0.01, d.f. = 6). Tukey‐HSD groups comparing the different cultivars are also presented. Full statistical analysis can be found in Tables S18 (cherry day‐10 populations), S19 (plum day‐10 populations) and S20 (cherry cultivars).

The leaf population count method was validated using all strains in this study (Fig. S6; Table S21) and produced a similar differentiation between strains as that observed in the whole‐tree glasshouse experiment. Psm R1 strains varied in aggressiveness; Psm R2 and Pss multiplied to high levels and the non‐*Prunus* strains and nonpathogen Ps 9643 were unable to reach pathogen‐level growth. The leaf population assays were then repeated on cherry and plum using additional strains. The bacterial population counts 10 days post‐inoculation are presented in Figure 8a. Figure 8b shows several representative images of cherry leaves inoculated with cherry‐pathogenic strains and nonpathogens, illustrating the clear differentiation achieved based not only on populations but also on symptom development. Differentiation was less distinct in plum leaves, in which the designated nonpathogens multiplied to higher levels *in planta*. For example, RMA1 was not significantly restricted compared to some of the *Prunus*‐isolated pathogenic strains; however, it caused little or no tissue discolouration.

To determine if there were differences in multiplication in different cherry varieties, strains of the three cherry‐infecting pathovars, Psm R1‐5244, Psm R2‐leaf and Pss 9097, were compared in cvs Merton Glory, Napoleon, Roundel and Van (Fig. 8c). In contrast to the assays of symptom development on trees and cut shoots, Merton Glory did not display comparative resistance to infection, with population counts not significantly different from those in cvs Van and Roundel.

**Figure 9:**
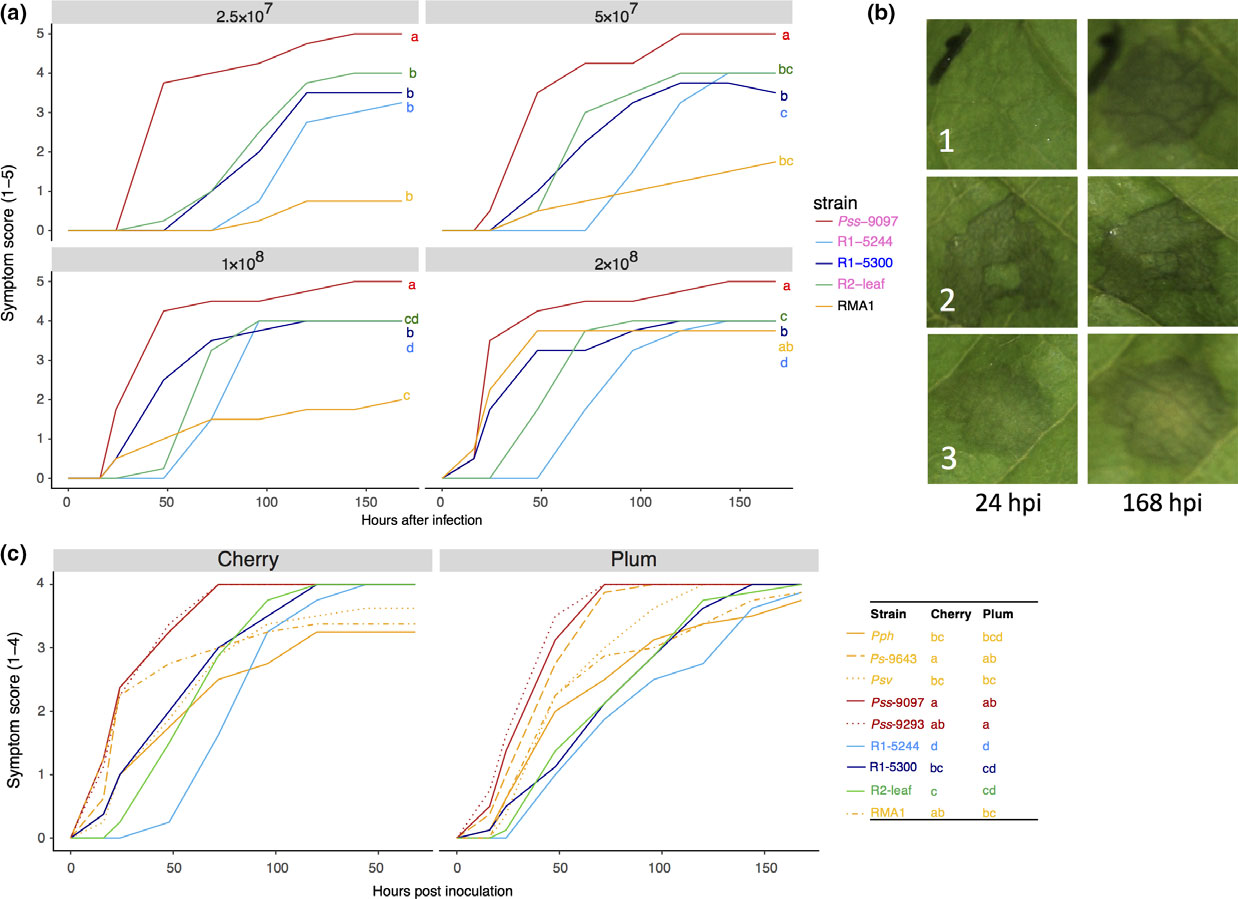
Symptom score analysis on cherry and plum. (a) Symptom development over time after inoculation of various *Pseudmonas syringae* strains in leaves of cherry cv. Van at different concentrations. Strains isolated from cherry are labelled in pink, whilst those from plum are in blue. Symptoms were scored from 0 to 5: 0, no symptoms; 1, limited browning; 2, browning <50% of inoculated site; 3, browning >50% of inoculated site; 4, complete browning; 5, spread from site of inoculation. Data presented are the means (*n *= 4). This experiment was performed once. Symptom development over time (0–48 h) was analysed using AUDPC. An ANOVA revealed significant differences between strains (*P *< 0.01, d.f. = 4), concentrations (*P *< 0.01, d.f. = 3) and a significant interaction (*P *< 0.01, d.f. = 12). Tukey‐HSD (*P *= 0.05, confidence level: 0.95) significance groups are presented. (b) Typical symptom development 24 and 168 h post‐inoculation (hpi): 1, Psm R1‐5244; 2, Pss‐9097; 3, RMA1. (c) Symptom development over time for multiple strains on cherry and plum leaves. Symptoms were scored as in (a), but there is no score 5 as no spreading was observed in these experiments. Data presented are the mean values from two independent experiments (*n *= 8). ANOVAs of AUDPC values (0–48 hpi) for cherry and plum revealed significant differences between strains (*P *< 0.01, d.f. = 8). Tukey‐HSD (*P *= 0.05, confidence level: 0.95) significance groups are presented in the table next to the plot. Full statistical analysis can be found in Tables S22 (variable concentrations), S23 (AUDPC cherry leaves 0–48 h) and S24 (AUDPC plum leaves 0–48 h). AUDPC data can be found in Tables S25, S26 and S27 (variable concentration, cherry and plum experiments, respectively).

At the low concentrations of inoculum used for population counts (2 × 10^6^ mL^−1^), the nonpathogens failed to produce any symptoms in cherry leaves. To assess if they caused a HR, higher concentrations were tested (≥10^8^ mL^−1^). The nonpathogenic strains were found to cause the rapid appearance of brown/black lesions within 48 h indicative of the activation of the HR (Fig. 9a), whereas lesion formation by Psm R1 and Psm R2 was significantly delayed. Data presented in Figure 9a illustrate the differential observed with the nonpathogens RMA1 and R1‐5300 compared with R1‐5244 and R2‐leaf at 2 × 10^8^ CFU mL^−1^. Intriguingly, the pathogenic Pss 9097 caused the most rapid symptom appearance at all concentrations of inoculum (Fig. 9a), and in some replicates spread slightly from the point of inoculation (score = 5). Figure 9b shows the different responses observed 24 h and 168 h after inoculation for three representative strains: symptom development after inoculation with the pathogenic R1‐5244 was delayed, whilst symptoms developed rapidly after inoculation with Pss 9097 and RMA1.

Induction of the HR was further examined using additional strains in both cherry and plum leaves (Fig. 9c). This experiment confirmed the rapid symptom production by Pss in both hosts and showed that the plum strain R1‐5300 exhibited differential behaviour, triggering HR‐like symptoms in cherry but not in its host of origin. Some of the nonpathogens of cherry caused clear HR‐like symptoms. Both RMA1 and Ps 9643 produced symptoms significantly more rapidly than pathogenic R1‐5244 and R2‐leaf in cherry. Reactions to Pph 1448A and Psv 631 were less distinctive and not significantly different from R2‐leaf. There was less clear separation of strains based on symptom severity on plum leaves, but the timing of collapse again separated pathogens from nonpathogens. The progress of symptom appearance over time in representative cherry and plum leaves is shown in Figure S7.

## Discussion

The combination of phylogenetics and pathogenicity testing described here highlights the complexity of *P. syringae* and the difficulties associated with differentiation into pathovars and races. The distinction of pathovars is historically based on the production of characteristic disease symptoms in a specific host plant, for example in halo blight of bean caused by *P. syringae* pv. *phaseolicola* (Arnold *et al*., 2011). The pathogenicity of specialist pathovars on a particular host has been linked to the possession of a discrete set of proteinaceous Type III effectors that act as virulence factors adapted to suppress host immunity (Sarkar *et al*., 2006). Bacterial canker of *Prunus* breaks the established pathovar dogma because several unrelated strains of *P. syringae* cause the same symptoms in cherry and plum. Further unravelling of the genomes of the canker pathogens may reveal that a distinct set of effectors is required for virulence and is present in members of the unrelated clades.

Psm was originally divided into two races based on morphological differences and differential aggressiveness on two cultivars of cherry (Freigoun & Crosse, 1975). In other pathovars, complex race structures have been identified based on avirulence/virulence reactions on large sets of host cultivars. Genetic dissection has allowed the functional characterization of certain Type III effectors as avirulence factors and also the identification of matching resistance (*R*) genes in cultivars of the host plant (Arnold *et al*., 2011). Race structures have been shown to be based on effector‐triggered immunity (ETI) and induction of the HR in resistant host varieties (Jones & Dangl, 2006). The race structure currently proposed for Psm is misleading. Phylogenetics revealed that Psm R1 and Psm R2 are distantly related, forming distinct monophyletic clades within phylogroups 3 and 1, respectively, and have recently been classified as separate species within the *P. syringae* complex (Bull *et al*., 2010; Scortichini *et al*., 2013). This study failed to clearly separate Psm R1 and Psm R2 based on aggressiveness towards different cultivars; however, differences in experimental procedure, strains and climatic conditions prevent a direct comparison with historical results. The original studies used fully mature trees, which may exhibit contrasting resistance to the young trees used in this study (Freigoun & Crosse, 1975). It seems likely that the field differentiation of Psm R1 and Psm R2 recorded in previous studies is not based solely on single‐gene mediated ETI, but is dictated by a set of quantitative traits in both host and pathogen. By contrast, the clear differentiation of Psm R1 plum strains from cherry strains was observed in all pathogenicity assays tested. Strains of Psm R1 isolated from plum, such as R1‐5300, caused a rapid HR and failed to multiply in cherry leaves, but were pathogenic in plum tissues. Such a clear differential is more representative of ETI in action. These results support studies done at East Malling on Psm R1 host specificity (Crosse & Garrett, 1970). The cherry strain R1‐9657 was reduced in aggressiveness on cherry and may represent an intermediate between the groups. The induction of the HR in cherry by pathogens of other plants, such as RMA1, was also indicative of ETI regulating nonhost resistance.

Unlike Psm, the strains of Pss isolated from *Prunus* were not monophyletic and were found throughout phylogroup 2. Cherry and plum strains did not form distinct clades and all were interspersed with other strains designated as Pss isolated from other host plants. Strains were originally designated as Pss based on their pathogenicity to lilac. Their wide host range means that the pathovar concept does not really apply as in other *P. syringae* pathovars. They also had a much more necrotrophic mode of parasitism than Psm (preliminary electron microscopy has confirmed the biotrophic multiplication of Psm R2 in leaves of cherry cv. Van; Fig. S8). The speed of formation of lesions by Pss on cherry fruits and leaves was striking. Studies have shown that the aggressiveness of Pss to various host plants may be based more on low molecular weight phytotoxin production rather than effector‐mediated suppression of immunity (Dudnik & Dudler, 2014). Cherry fruit tissue may be particularly sensitive to toxins (Scholz‐Schroeder *et al*., 2001). The lack of genetic similarity amongst the *Prunus*‐pathogenic Pss strains raises the possibility that they may use very different sets of virulence factors to cause the same canker disease. As such, future breeding efforts should use more than one strain of Pss to ensure resistance acts against all possible pathogens.

This study aimed to assess several different laboratory‐based pathogenicity assays. Results were compared to a field experiment where strains were inoculated through leaf scars and wounds, which represent natural entry points for this disease. The field test revealed differences between strains, where only those strains designated as pathogens in the glasshouse experiment were associated with lesion lengths significantly different from the 10 mm MgCl_2_ control. It also revealed cultivar‐specific differences in susceptibility, with Merton Glory exhibiting broad resistance to all pathogenic clades. The field inoculations were assessed using both disease score and symptom length. The percentage of inoculation sites with high disease scores was significantly reduced in leaf scars compared to wound inoculations. The leaf scar may act as a barrier to infection and reduce bacterial concentrations as the bacterial population becomes bottle‐necked (Crosse, 1966). For symptom lengths, results were highly variable, with most inoculations only spreading slightly. This could be influenced by a range of climatic factors such as frost, which could promote symptom development (Lamichhane *et al*., 2014). There was some contamination of controls with wild pseudomonads, as in previous studies (Freigoun & Crosse, 1975), which highlights the prevalence of this disease.

**Figure 10:**
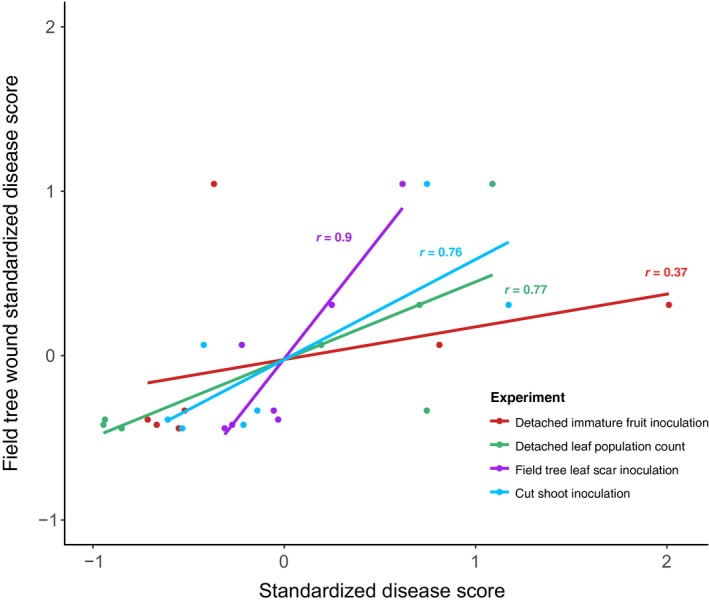
Correlation between results from various pathogenicity tests with the length of symptoms recorded after wound inoculation of field trees. The wound inoculation on field trees was assumed to be most representative of the natural disease. The datasets included field leaf scar symptom length (Fig. 3a), cut shoot percentage area of brown/black symptoms (Fig. 5), immature cherry fruit symptom diameter (Fig. 6a) and bacterial population counts in CFU mL ^−1^ (Fig. S6). Each dataset included the results for seven bacterial strains on cherry cv. Van. Data were standardized (mean⁄SD) to allow correlation analysis to be performed. A linear model line was plotted for each experiment and Pearson's correlation coefficients are presented. Data used and the correlation results are presented in Tables S28 and S29.

Apart from cherry fruit inoculation, all of the laboratory‐based tests distinguished pathogens from nonpathogenic *P. syringae* on cherry. To provide an objective assessment of the suitability of the different tests to reflect natural levels of disease, results obtained for each strain on cv. Van were correlated with lesion length data recorded following wound inoculation in the field, which were considered a true indication of disease potential (Fig. 10). In contrast to fruit assays (*r *= 0.37), field leaf scar, cut shoot and detached leaf population assays all correlated well with the field wound results (*r *> 0.70). In terms of identifying cultivar differences in susceptibility, both the leaf and fruit assays failed to differentiate Merton Glory from other cultivars. The cut shoot assay proved to be the best laboratory‐based assessment and identified the broad‐spectrum reduced susceptibility of cherry cv. Merton Glory. However, as found in the field inoculations, there was a large degree of variability of symptom development in the shoot test. This variability in symptoms appeared to depend on the cultivar response. If the cultivar was resistant, variability was low, but high if the cultivar was susceptible. The variability of the field and shoot tests highlights the importance of having sufficient replication to establish quantitative differences.

Although unable to differentiate cultivar susceptibility, the cherry leaf population counts and symptom scoring could reliably discriminate pathogenic and nonpathogenic strains. These two tests could therefore be integrated to characterize strains. Nonpathogenic strains induced a putative HR at high inoculation concentration but failed to multiply to the same level as pathogens when inoculated from a lower concentration. However, the plum leaf population assay was not consistent with field results, as the nonpathogen RMA1 was able to grow to similar levels to the cherry pathogens. In the plum cut shoot assay RMA1 also caused brown/black symptoms. The field experiment showed that RMA1 is not a true pathogen of plum; however, its pathogenicity in the laboratory‐based assays may indicate it has adaptive potential to cause disease when inoculated in unnaturally high concentrations directly onto plant tissue. Plum has the potential to act as a host plant for the strain, as the bacteria were able to multiply.

Based on the efficacy of the different tests assessed, the results suggest that the efficient screening of *Prunus* germplasm for resistance should include the following sequence of assays, using various strains from each of the different pathogenic clades: (i) rapid leaf inoculation tests to detect any HR‐based resistance due to the presence of major *R* genes; (ii) cut shoot tests to confirm the presence of *R* genes and interrogate quantitative differences; then, based on these results, (iii) a subset of desirable genotypes would be screened by whole‐tree inoculations through both wounds and leaf scars. It is important to include leaf scar infection as the results showed significant differences between inoculations as the morphology of the scar may provide a physical barrier before cellular interactions define the success or failure of strains to colonize and cause disease (Crosse, 1966). The final assessment would be in the field, bearing in mind the often‐reported lack of correlation between laboratory/glasshouse and field performance (Farhadfar *et al*., 2016). Future genetic studies of resistance would require multiyear repetition to ensure results are consistent in varying climatic conditions and stages of plant development.

Breeding resistance to at least three rather distinct groups of a pathogen, Psm R1 and R2 and Pss, remains a challenging prospect and may require pathogen mixtures to be used in routine screening. Results showed that representatives of the three clades of *P. syringae* containing bacterial canker pathogens vary in aggressiveness and therefore may trigger differing host resistance mechanisms in cherry. In addition to screening untested genotypes, further research is much needed on the biochemical and genetic basis of broad‐spectrum resistance already identified in cultivars such as Merton Glory that restrict disease development following challenge by each of the three clades of canker pathogen.

## Supporting information


**Figure S1.** Images of immature cherry fruits inoculated with *Pseudomonas syringae* pv. *morsprunorum* race 1 strains. Images were taken 10 dpi. Five replicate cherries were inoculated per strain. Strains from cherry are labelled in pink, whilst those from plum are in blue.

**Figure S2.** Images of immature cherry fruits inoculated with *Pseudomonas syringae* pv. *morsprunorum* race 2 strains. Images were taken 10 dpi. Five replicate cherries were inoculated per strain. All strains were from cherry.

**Figure S3.** Images of immature cherry fruits inoculated with *Pseudomonas syringae* pv. *syringae* strains. Images were taken 10 dpi. Five replicate cherries were inoculated per strain. Strains from cherry are labelled in pink, whilst those from plum are in blue.

**Figure S4.** Images of immature cherry fruits inoculated with previously designated nonpathogenic strains in the glasshouse whole‐tree experiment and a 10 mm MgCl_2_ control. Images were taken 10 dpi. Five replicate cherries were inoculated per strain.

**Figure S5.** Symptoms observed in detached cherry leaves using different inoculation methods. Representative images of the four methods: infiltration, stab, droplet, and wound + droplet. Leaves show inoculation with R1‐5244 or a 10 mm MgCl_2_ control.

**Figure S6.** Boxplot of day‐10 population counts of all strains used in this study on cherry cv. Van leaves. Strains isolated from cherry are labelled in pink, whilst those from plum are in blue. The boxplots are coloured by clade: *P. syringae* pv. *morsprunorum* (Psm) race 1 (R1), blue; Psm race 2 (R2), green; *P. syringae* pv. *syringae* (Pss), red; nonpathogens (*P. syringae* pv*. phaseolicola* 1448A, Pph; *P. syringae* pv. *avellanae* 631, Psv; *P. syringae* RMA1), orange. The 10 mm MgCl_2_ control is not included as no bacteria were found. The data presented are all values for each treatment (*n *= 9). This complete experiment was performed once. An ANOVA revealed significant differences between strains (*P *< 0.01, d.f. = 20). Tukey‐HSD (*P *= 0.05, confidence level: 0.95) significance groups for the different strains are presented. Full statistical analysis can be found in Table S18.

**Figure S7.** Images of symptom development over time on cherry and plum. (a) Cherry cv. Van, (b) plum cv. Victoria. The same leaf was imaged 16, 24, 48 and 72 h post‐inoculation. Arrows indicate the first appearance of symptoms for that particular strain. Strains are labelled: 1, R1‐5244; 2, R1‐5300; 3, R2‐leaf; 4, Ps 9643; 5, Pss 9097; 6, Pss 9293; 7, RMA1; 8, Psv 631; 9, Pph 1448A; C, 10 mm MgCl_2_ control.

**Figure S8.** Transmission electron microscope images of *Pseudomonas syringae* pv. *morsprunorum* R2‐leaf in a detached cherry leaf, 1 week after inoculation. Electron microscopy was performed by Ian Brown (University of Kent) on infected cherry leaves. Detached leaves were infiltrated with bacteria at 2 × 10^6^ CFU mL^−1^ and incubated for 1 week at 22 °C. Microscopy was then performed on inoculation sites as previously described (Soylu *et al*., 2005). (a) Bacteria colonizing the apoplastic space next to live mesophyll cells. (b) Cell wall alterations (papilla formation) shown by arrows in plant cells. (c) A bacterial colony containing putatively dead and live bacteria next to plant cells.

**Table S1.** Validation of spectrophotometer‐based concentration measurements of *Pseudomonas syringae* cultures.

**Table S2.** All strains used in phylogenetic analysis in addition to those used for pathogenicity testing in this study.

**Table S3.** Environmental data for the field experiment conducted October 2015–June 2016.

**Table S4.** Genome assembly statistics for all *Pseudomonas syringae* strains sequenced in this study.

**Table S5.** Proportional odds model (POM) analysis of the glasshouse whole‐tree wound inoculations.

**Table S6.** Proportional odds model (POM) analysis of the cherry field inoculations.

**Table S7.** Lsmean Tukey‐HSD groupings for different treatment combinations from the proportional odds model (POM) analysis of cherry field inoculations.

**Table S8.** REML analysis of field inoculation of cherry inoculated by leaf scar.

**Table S9.** REML analysis of field inoculation of cherry inoculated by wound.

**Table S10.** Proportional odds model (POM) analysis of the plum field inoculations.

**Table S11.** Lsmeans Tukey‐HSD groupings for different treatment combinations from the proportional odds model (POM) analysis of plum field inoculations.

**Table S12.** REML analysis of field inoculation of plum inoculated by leaf scar.

**Table S13.** REML analysis of field inoculation of plum inoculated by wound.

**Table S14.** ANOVA table of cut shoot inoculations.

**Table S15.** ANOVA table of immature cherry fruit inoculations.

**Table S16.** REML analysis of immature cherry fruit inoculations where bacterial strains were inoculated onto different host cultivars.

**Table S17.** ANOVA table of day‐10 leaf population counts of different bacterial strains inoculated on cherry and plum.

**Table S18.** REML analysis of day‐10 leaf population counts of reference bacterial strains inoculated on cherry leaves.

**Table S19.** REML analysis of day‐10 leaf population counts of reference bacterial strains inoculated on plum leaves.

**Table S20.** ANOVA table of day‐10 leaf population counts of different bacterial strains inoculated on different cherry cultivars.

**Table S21.** ANOVA table of leaf population counts of all strains used in this study.

**Table S22.** ANOVA table of AUDPC analysis of symptom score on leaves of several bacterial strains inoculated at different concentrations.

**Table S23.** ANOVA table of AUDPC analysis of leaf symptom score over time of different bacterial strains inoculated on cherry.

**Table S24.** ANOVA table of AUDPC analysis of leaf symptom score over time of different bacterial strains inoculated on plum.

**Table S25.** AUDPC values based on symptom development 0–48 h after inoculation for several bacterial strains on cherry leaves inoculated at different starting concentrations (0.5 × 10^7^, 10^7^, 10^8^, 2 × 10^8^ CFU mL^−1^).

**Table S26.** AUDPC values based on symptom development 0–48 h after inoculation for several bacterial strains on cherry leaves during two independent experiments.

**Table S27.** AUDPC values based on symptom development 0–48 h after inoculation for several bacterial strains on plum leaves during two independent experiments.

**Table S28.** Data used in correlation analysis for Figure 10.

**Table S29.** Correlation coefficients calculated for each different inoculation experiment correlated with the symptom length results of the field tree wound inoculation experiment.

**Text S1.** Supplementary methods.
