# Supplementary figures and images for: Characterization of the pathogenicity of strains of Pseudomonas syringae towards cherry and plum
Source: Plant Pathol. 2018 Feb 14;67(5):1177–93. doi: 10.1111/ppa.12834 (PMC5993217; doi:10.1111/ppa.12834)

R2-leaf

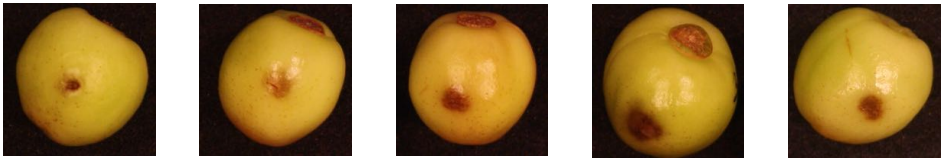

R2-5255

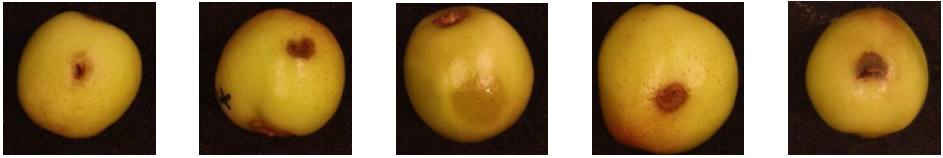

R2-SC214

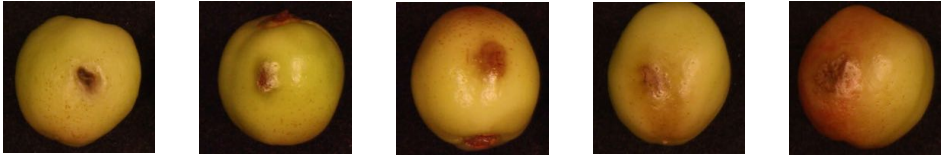

R2-5260

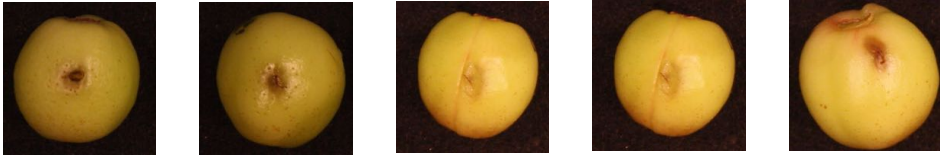

Supplement: Supplementary file 2 — Figure S2. Images of immature cherry fruits inoculated with Pseudomonas syringae pv. morsprunorum race 2 strains. Images were taken 10 dpi. Five replicate cherries were inoculated per strain. All strains were from cherry. [file PPA-67-1177-s002.pdf]

*Pss-9654*

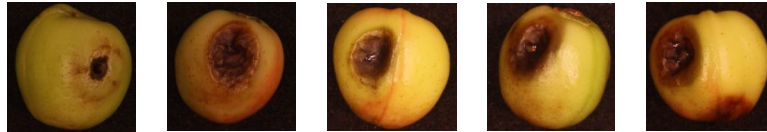

*Pss-9656*

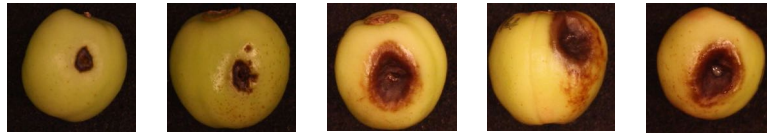

*Pss-9630*

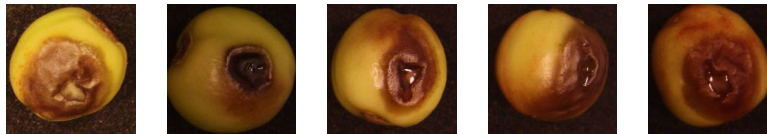

*Pss-9644*

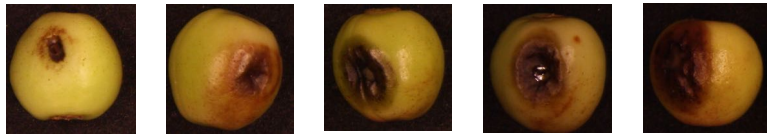

*Pss-9097*

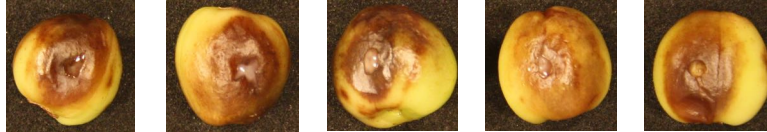

*Pss-9659*

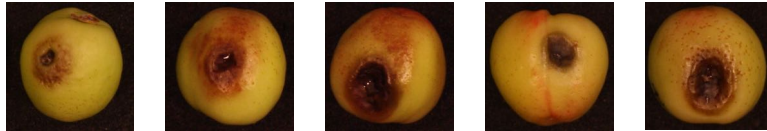

*Pss-9293*

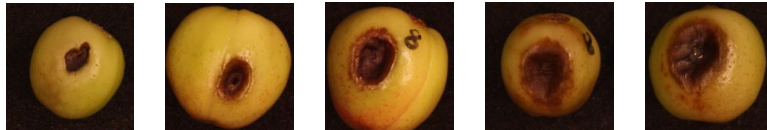

Supplement: Supplementary file 3 — Figure S3. Images of immature cherry fruits inoculated with Pseudomonas syringae pv. syringae strains. Images were taken 10 dpi. Five replicate cherries were inoculated per strain. Strains from cherry are labelled in pink, whilst those from plum are in blue. [file PPA-67-1177-s003.pdf]

RMA1

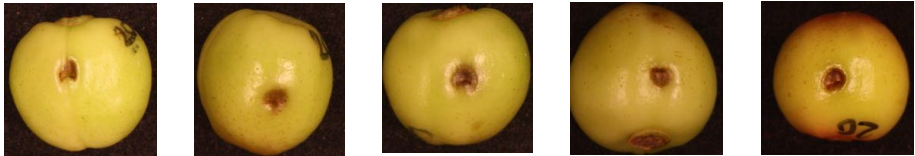

*Pph*

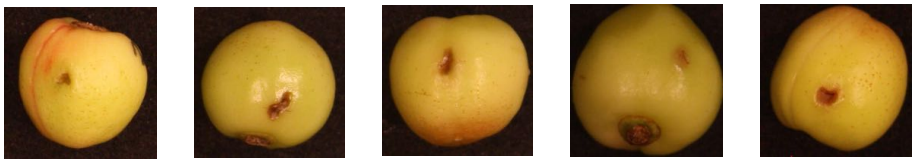

*Psv*

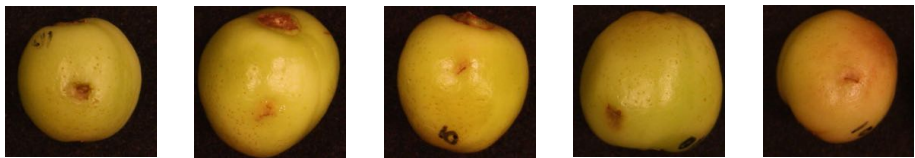

*Ps-9643*

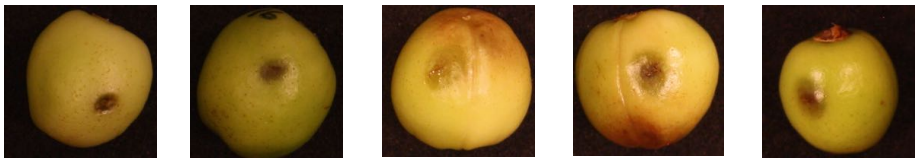

Control

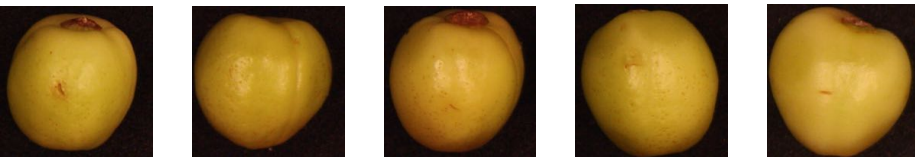

Supplement: Supplementary file 4 — Figure S4. Images of immature cherry fruits inoculated with previously designated nonpathogenic strains in the glasshouse whole‐tree experiment and a 10 mm MgCl2 control. Images were taken 10 dpi. Five replicate cherries were inoculated per strain. [file PPA-67-1177-s004.pdf]

|                   | Psm R1                                                                               | Control                                                                               |
|-------------------|--------------------------------------------------------------------------------------|---------------------------------------------------------------------------------------|
| Infiltration      | 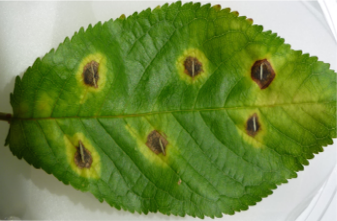   | 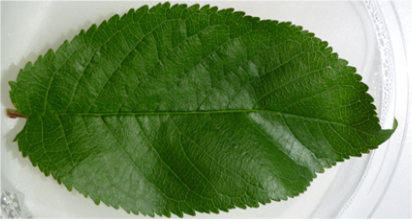   |
| Stab              | 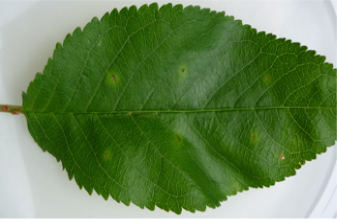   | 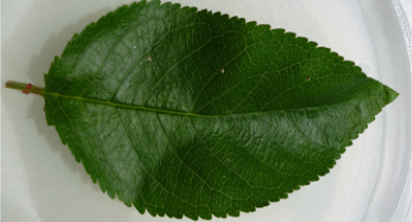   |
| Droplet           | 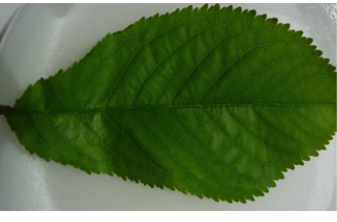  | 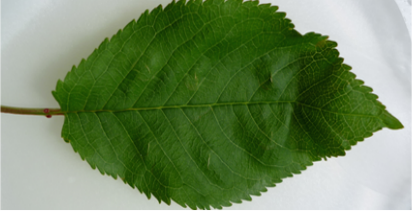  |
| Wound and Droplet | 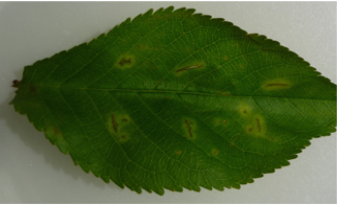 | 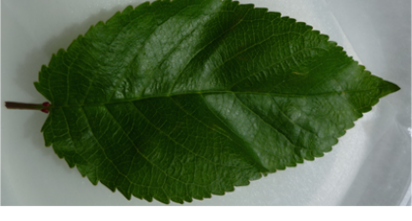 |

Supplement: Supplementary file 5 — Figure S5. Symptoms observed in detached cherry leaves using different inoculation methods. Representative images of the four methods: infiltration, stab, droplet, and wound + droplet. Leaves show inoculation with R1‐5244 or a 10 mm MgCl2 control. [file PPA-67-1177-s005.pdf]

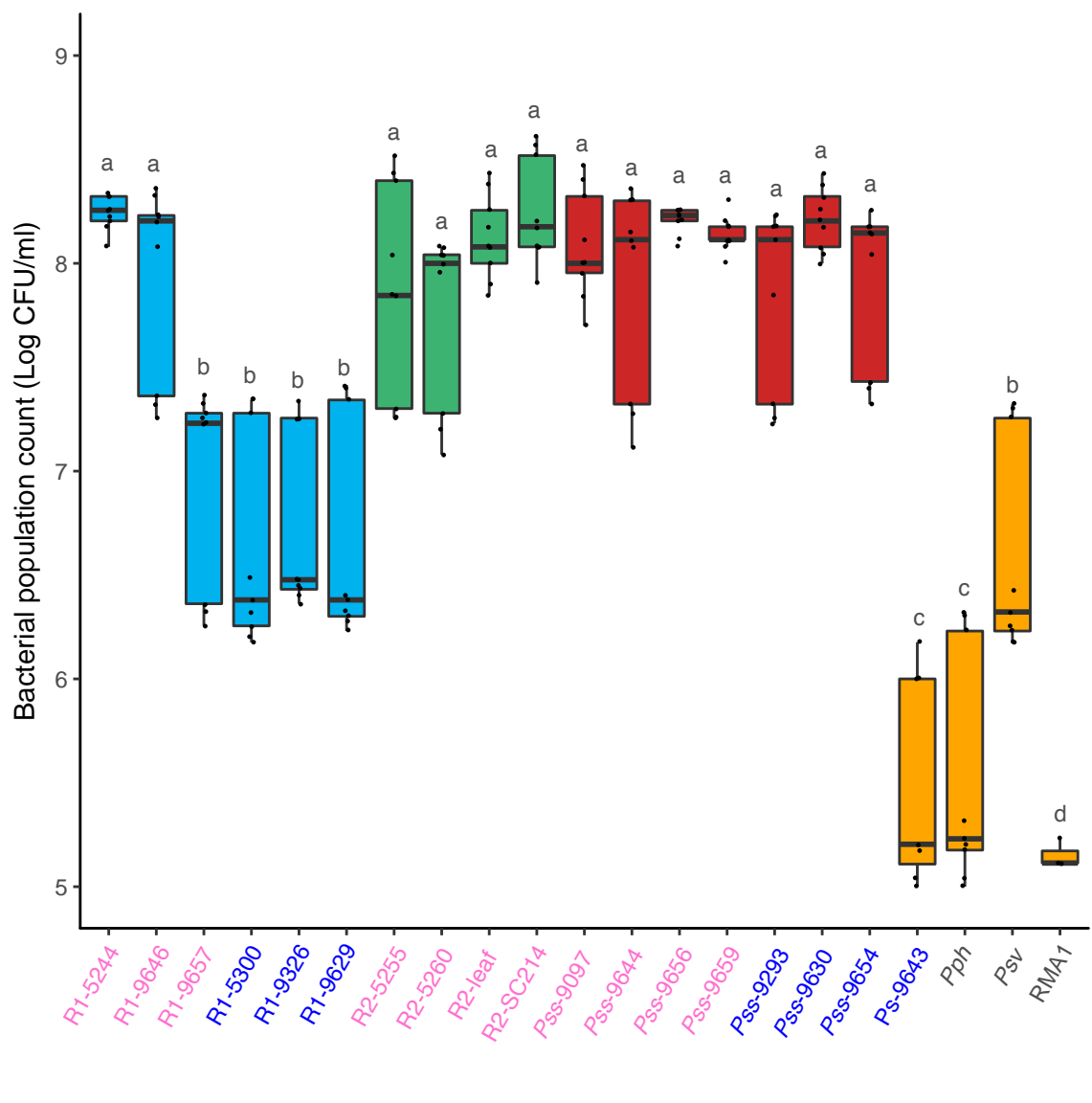

Supplement: Supplementary file 6 — Figure S6. Boxplot of day‐10 population counts of all strains used in this study on cherry cv. Van leaves. Strains isolated from cherry are labelled in pink, whilst those from plum are in blue. The boxplots are coloured by clade: P. syringae pv. morsprunorum (Psm) race 1 (R1), blue; Psm race 2 (R2), green; P. syringae pv. syringae (Pss), red; nonpathogens (P. syringae pv. phaseolicola 1448A, Pph; P. syringae pv. avellanae 631, Psv; P. syringae RMA1), orange. The 10 mm MgCl2 control is not included as no bacteria were found. The data presented are all values for each treatment (n = 9). This complete experiment was performed once. An ANOVA revealed significant differences between strains (P < 0.01, d.f. = 20). Tukey‐HSD (P = 0.05, confidence level: 0.95) significance groups for the different strains are presented. Full statistical analysis can be found in Table S18. [file PPA-67-1177-s006.pdf]

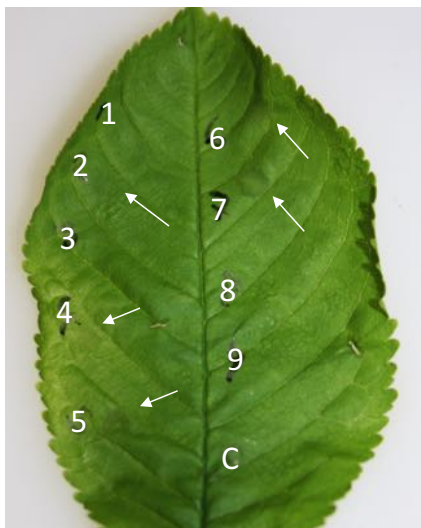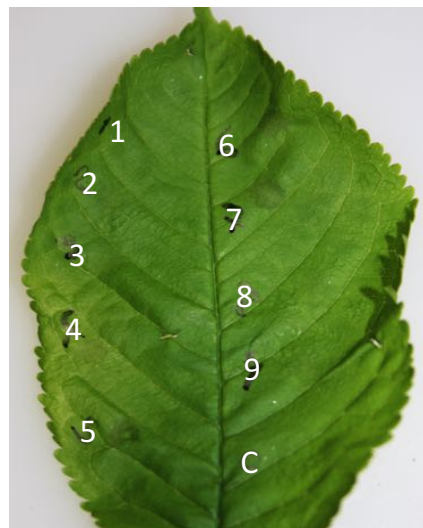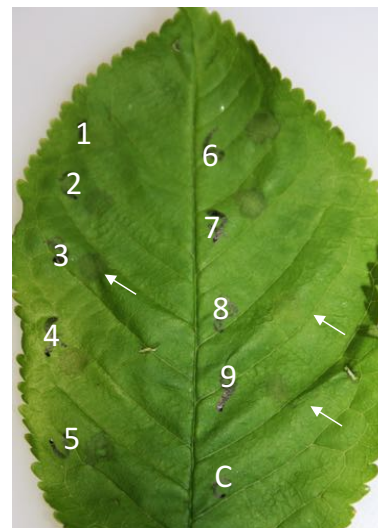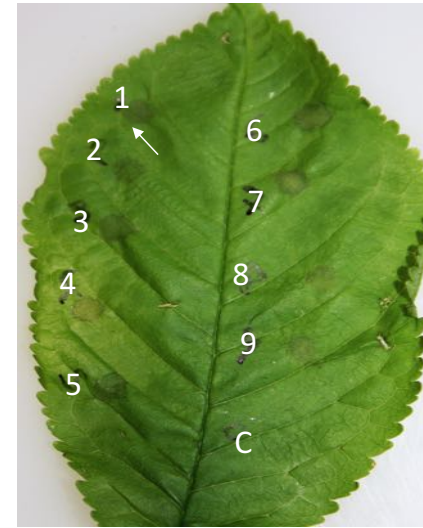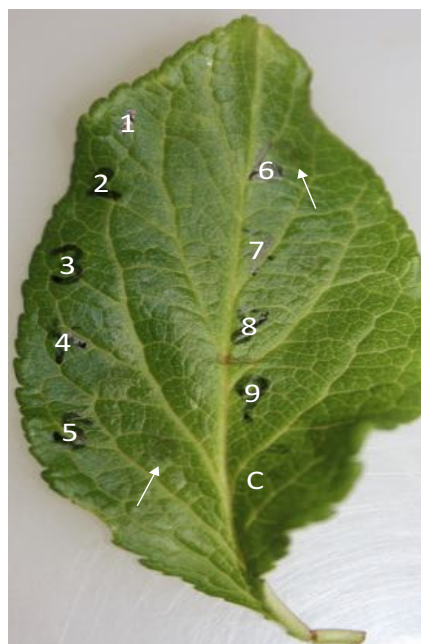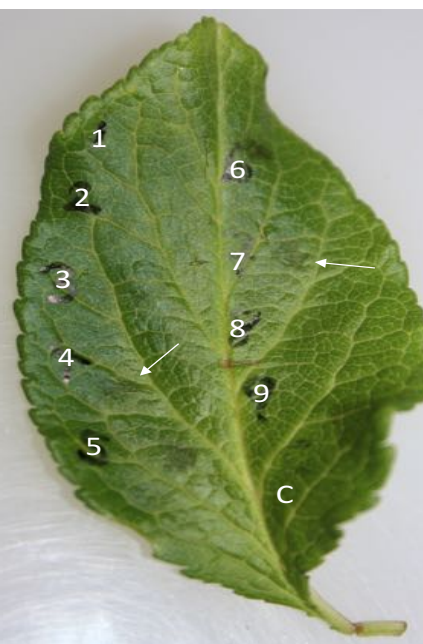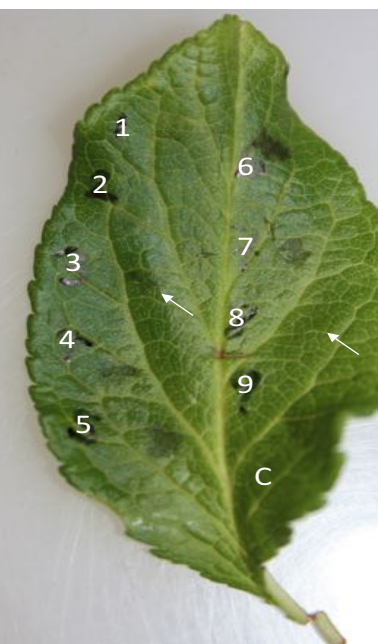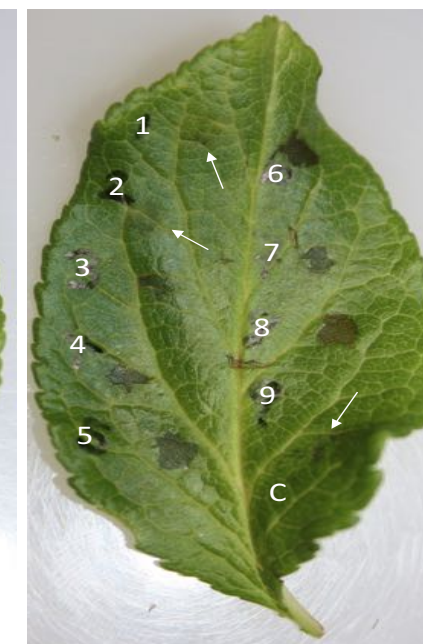

16

24

48

72

Supplement: Supplementary file 7 — Figure S7. Images of symptom development over time on cherry and plum. (a) Cherry cv. Van, (b) plum cv. Victoria. The same leaf was imaged 16, 24, 48 and 72 h post‐inoculation. Arrows indicate the first appearance of symptoms for that particular strain. Strains are labelled: 1, R1‐5244; 2, R1‐5300; 3, R2‐leaf; 4, Ps 9643; 5, Pss 9097; 6, Pss 9293; 7, RMA1; 8, Psv 631; 9, Pph 1448A; C, 10 mm MgCl2 control. [file PPA-67-1177-s007.pdf]

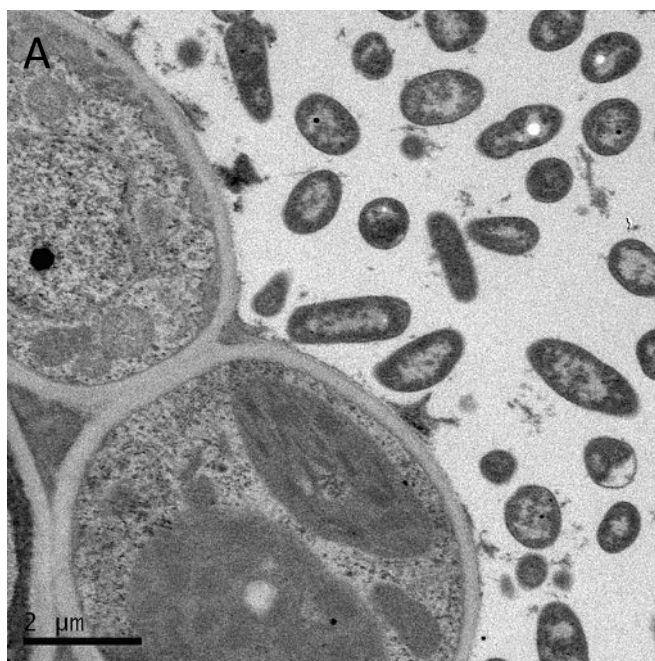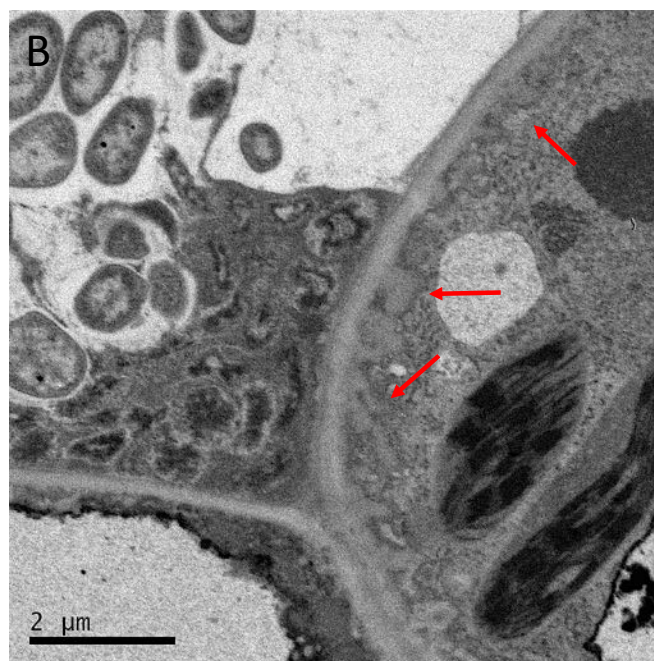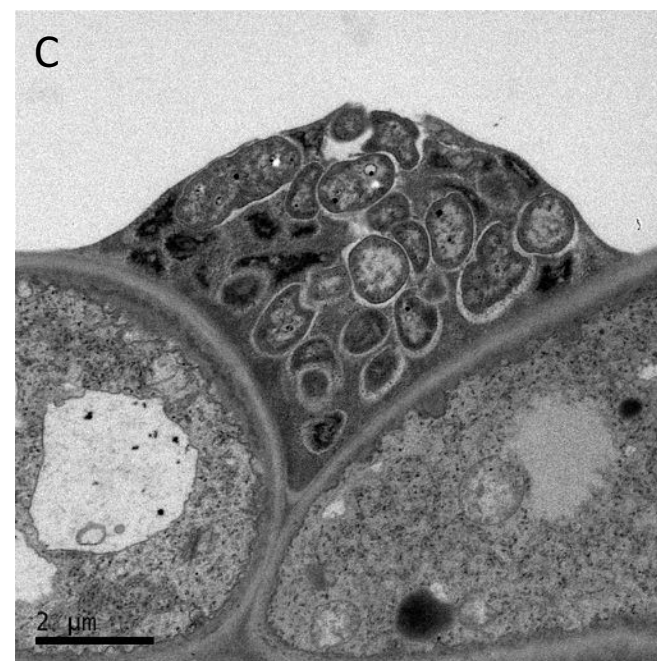

Supplement: Supplementary file 8 — Figure S8. Transmission electron microscope images of Pseudomonas syringae pv. morsprunorum R2‐leaf in a detached cherry leaf, 1 week after inoculation. Electron microscopy was performed by Ian Brown (University of Kent) on infected cherry leaves. Detached leaves were infiltrated with bacteria at 2 × 10^6^ CFU mL−1 and incubated for 1 week at 22 °C. Microscopy was then performed on inoculation sites as previously described (Soylu et al., 2005). (a) Bacteria colonizing the apoplastic space next to live mesophyll cells. (b) Cell wall alterations (papilla formation) shown by arrows in plant cells. (c) A bacterial colony containing putatively dead and live bacteria next to plant cells. [file PPA-67-1177-s008.pdf]
