# Supplementary material for: Characterization of the pathogenicity of strains of Pseudomonas syringae towards cherry and plum
Source: Plant Pathol. 2018 Feb 14;67(5):1177–93. doi: 10.1111/ppa.12834 (PMC5993217; doi:10.1111/ppa.12834)
Supplement: Supplementary file 9 — Table S1. Validation of spectrophotometer‐based concentration measurements of Pseudomonas syringae cultures. [file PPA-67-1177-s009.docx]

| Strain | CFU/ml |
| --- | --- |
| *Psm* R1-5244 | 7.4x10^7^ |
| *Psm* R1-5300 | 1.06x10^8^ |
| *Psm* R1-9326 | 1.075x10^8^ |
| *Psm* R1-9646 | 1.52x10^8^ |
| *Psm* R1-9657 | 8.8x10^7^ |
| *Psm* R2-5255 | 8.2x10^7^ |
| *Psm* R2-5260 | 1.72x10^8^ |
| *Psm* R2-SC214 | 9.6x10^7^ |
| *Pss* 9097 | 1.02x10^8^ |
| *Pss* 9293 | 2.8x10^8^ |
| *Pss* 9630 | 1x10^8^ |
| *Pss* 9644 | 6.5x10^7^ |
| *Pss* 9654 | 1.075x10^8^ |
| *Pss* 9656 | 9.7x10^7^ |
| *Ps* 9643 | 8.8x10^7^ |
| RMA1 | 1.9x10^8^ |

**Table S1: Validation of spectrophotometer-based concentration measurements of *P. syringae* cultures.** All strains were diluted to a concentration of approximately 2x10^8^ CFU/ml based on spectrophotometer absorbance at OD_600_. The table lists the corresponding concentration of these strains based on dilution plating of each culture.
