# Supplementary material for: Characterization of the pathogenicity of strains of Pseudomonas syringae towards cherry and plum
Source: Plant Pathol. 2018 Feb 14;67(5):1177–93. doi: 10.1111/ppa.12834 (PMC5993217; doi:10.1111/ppa.12834)
Supplement: Supplementary file 10 — Table S2. All strains used in phylogenetic analysis in addition to those used for pathogenicity testing in this study. [file PPA-67-1177-s010.docx]

| Strain | Pathovar | Phylogroup | Host/Isolate source | | Reference | | BioProject/accession | |  |
| --- | --- | --- | --- | --- | --- | --- | --- | --- | --- |
| acer302273 | *aceris* | 2 | *Acer* sp. | | Baltrus et al. 2011 | | AEAO00000000 | |  |
| act302091 | *actinidiae* | 1 | *Actinidia deliciosa* | | Baltrus et al. 2011 | | AEAL00000000 | |  |
| aes2250 | *aesculi* | 3 | *Aesculus hippocastanum* | | Green *et al.* 2010 | | ACXT00000000 | |  |
| aes3681 | *aesculi* | 3 | *Aesculus hippocastanum* | | Green *et al.* 2010 | | ACXS00000000 | |  |
| amy3205 | *amygdali* | 3 | *Prunus dulcis* | | Bartoli *et al.* 2015 | | JYHB00000000 | |  |
| amyICMP3918 | *amygdali* | 3 | *Prunus dulcis* | | Thakur *et al.* 2016 | | LJPQ00000000 | |  |
| aptaDSM50255 | *aptata* | 2 | *Beta vulgaris* | | Baltrus et al. 2011 | | AEAN00000000 | |  |
| atroDSM50255 | *atrofaciens* | 2 | *Triticum aestivum* | | Baltrus *et al.* 2014a | | AWUI00000000 | |  |
| avelVe013 | *avellanae* | 2 | *Corylus avellana* | | O'Brien *et al.* 2012 | | AKCK00000000 | |  |
| avelVe037 | *avellanae* | 2 | *Corylus avellana* | | O'Brien *et al.* 2012 | | AKCJ00000000 | |  |
| avii3846 | *avii* | 1 | *Prunus avium* | | Nowell *et al.* 2016 | | LIIJ00000000 | |  |
| CC1557 | - | - | Snow | | Hockett *et al.* 2014 | | AVEH00000000 | |  |
| cera6109 | *cerasicola* | 3 | *Prunus yedoensis* | | Nowell *et al.* 2016 | | LIIG00000000 | |  |
| ceraICMP17524 | *cerasicola* | 3 | *Prunus yedoensis* | | Thakur *et al.* 2016 | | LJQA00000000 | |  |
| ciccICMP5710 | *ciccaronei* | 3 | *Ceratonia siliqua* | | Thakur *et al.* 2016 | | LJPY00000000 | |  |
| cit7 | - | 2 | *Citrus sinensis* | | Baltrus et al. 2011 | | AEAJ00000000 | |  |
| cunnICMP11894 | *cunninghamiae* | 3 | *Cunninghamia lanceolata* | | Thakur *et al.* 2016 | | LJQE00000000 | |  |
| daphICMP9757 | *daphniphylli* | 3 | *Daphniphyllum teijsmannii* | | Thakur *et al.* 2016 | | LJQF00000000 | |  |
| glycR4 | *glycinea* | 3 | *Glycine max* | | Qi *et al.* 2011 | | AEGH00000000 | |  |
| lach301315 | *lachrymans* | 3 | *Cucumis sativus* | | Baltrus et al. 2011 | | AEAF00000000 | |  |
| lach302278 | *lachrymans* | 1 | *Cucumis sativus* | | Baltrus et al. 2011 | | AEAM00000000 | |  |
| lapsaICMP3947 | *lapsa* | 2 | *Zea* sp. | | Thakur *et al.* 2016 | | LJQQ00000000 | |  |
| morsU7805 | *morsprunorum* | 3 | *Prunus mume* | | Mott *et al.* 2016 | | LGLQ00000000 | |  |
| Strain | *Pathovar* |  | *Host/Isolate source* | | Reference | | BioProject/accession | |  |
| myriICMP7118 | *myricae* | 3 | *Myrica rubra* | | Thakur *et al.* 2016 | | LJQV00000000 | |  |
| neriiICMP16943 | *savastanoi* | 3 | *Olea europea* | | Thakur *et al.* 2016 | | LJQW00000000 | |  |
| paniLMG2367 | *panici* | 2 | *Panicum miliaceum* | | Liu *et al.* 2012 | | ALAC00000000 | |  |
| papu1754 | *papulans* | 2 | *Malus sylvestris* | | Nowell *et al.* 2016 | | JYHI00000000 | |  |
| persNCPPB2254 | *persicae* | 1 | *Prunus persica* | | Zhao *et al.* 2015 | | LAZV00000000 | |  |
| photICMP7840 | *photiniae* | 3 | *Photinia glabra* | | Thakur *et al.* 2016 | | LJQO00000000 | |  |
| pisiPP1 | *pisi* | 2 | *Pisum sativum* | | Baltrus *et al.* 2014b | | AUZR00000000 | |  |
| R1-2341 | *morsprunorum* | 3 | *Prunus cerasus* | | Nowell *et al.* 2016 | | LIIB00000000 | |  |
| R1-5269 | *morsprunorum* | 3 | *Prunus cerasus* | | Nowell *et al.* 2016 | | LIHZ00000000 | |  |
| R2-302280 | *morsprunorum* | 2 | *Prunus domestica* | | Baltrus et al. 2011 | | AEAE00000000 | |  |
| R2-5261 | *morsprunorum* | 2 | *Prunus avium* | | Nowell *et al.* 2016 | | LIIA00000000 | |  |
| sava3335 | *savastanoi* | 3 | *Olea europea* | | Rodriguez-Palenzuela *et al.* 2010 | | ADMI00000000 | |  |
| sava4352 | *savastanoi* | 3 | *Olea europea* | | Thakur *et al.* 2016 | | LGKR00000000 | |  |
| soliICMP16925 | *solidagae* | 2 | *Solidago altissima* | | Thakur *et al.* 2016 | | JYHF00000000 | |  |
| syr1212 | *syringae* | 2 | *Pisum sativum* | | Baltrus *et al.* 2014a | | AVCR00000000 | |  |
| syr41a | *syringae* | 2 | *Prunus armeniaca* | | Bartoli *et al.* 2015 | | JYHJ00000000 | |  |
| syrB301D | *syringae* | 2 | *Pyrus communis* | | Ravindran *et al.* 2015 | | CP005969 | |  |
| syrB64 | *syringae* | 2 | *Triticum aestivum* | | Dudnik and Dudler 2013 | | ANZF00000000 | |  |
| syrB728a | *syringae* | 2 | *Phaseolus vulgaris* | | Feil *et al.* 2005 | | CP000075 | |  |
| syrHS191 | *syringae* | 2 | *Panicum miliaceum* | | Ravindran *et al.* 2015 | | CP006256 | |  |
| syrSM | *syringae* | 2 | *Triticum aestivum* | | Dudnik and Dudler 2013 | | APWT00000000 | |  |
| thea3923 | *theae* | 1 | *Camelia sinensis* | | Mazzaglia *et al.* 2012 | | AGNN00000000 | |  |
| tomDC3000 | *tomato* | 1 | *Solanum lycopersicum* | | Buell *et al.* 2003 | | AE016853 | |  |
| tomT1 | *tomato* | 1 | *Solanum lycopersicum* | | Almeida *et al.* 2009 | | ABSM00000000 | |  |
| ulmiICMP3962 | *ulmi* | 3 | *Ulmus* sp. | | Thakur *et al.* 2016 | | LJRQ00000000 |  |  |
|  |  |  |  |  |  |  | | |  |

**Table S2: All strains used in phylogenetic analysis in addition to those used for pathogenicity testing in this study**. Strain ID, pathovar, phylogroup, host/isolation source, reference to the publication of each genome and NCBI accession numbers are listed.
