# Supplementary material for: Characterization of the pathogenicity of strains of Pseudomonas syringae towards cherry and plum
Source: Plant Pathol. 2018 Feb 14;67(5):1177–93. doi: 10.1111/ppa.12834 (PMC5993217; doi:10.1111/ppa.12834)
Supplement: Supplementary file 11 — Table S3. Environmental data for the field experiment conducted October 2015–June 2016. [file PPA-67-1177-s011.docx]

| **Date** | **Max temperature** | **Min temperature** | **Mean temperature** | **Rainfall** | **Sunshine** | **%RH** | **Maximum humidity** | **Minimum humidity** |
| --- | --- | --- | --- | --- | --- | --- | --- | --- |
| 16/10/2015 | 11.4 | 8.2 | 15.5 | 1 | 0.2 | 88 | 96.1 | 74.4 |
| 17/10/2015 | 11.1 | 9.7 | 15.95 | 2.4 | 0 | 88 | 97.6 | 77.8 |
| 18/10/2015 | 12.1 | 9.6 | 16.9 | 0 | 0.2 | 86 | 95.5 | 70.9 |
| 19/10/2015 | 13.9 | 10 | 18.9 | 0 | 2.5 | 87 | 93.9 | 71.3 |
| 20/10/2015 | 13.2 | 3.1 | 14.75 | 0 | 1.6 | 87 | 100.1 | 79.7 |
| 21/10/2015 | 13.9 | 3.9 | 15.85 | 2.2 | 5.6 | 86 | 100.1 | 59.6 |
| 22/10/2015 | 13.3 | 9.3 | 17.95 | 3.6 | 0.8 | 93 | 98 | 82.8 |
| 23/10/2015 | 13.6 | 8.3 | 17.75 | 0 | 0.1 | 84 | 96.4 | 63.3 |
| 24/10/2015 | 12.8 | 9.9 | 17.75 | 0 | 0 | 78 | 86 | 69 |
| 25/10/2015 | 13.6 | 2.4 | 14.8 | 4.8 | 1.7 | 90 | 97.1 | 76.6 |
| 26/10/2015 | 12.4 | 1.3 | 13.05 | 0 | 5.3 | 87 | 100.1 | 56.7 |
| 27/10/2015 | 14.4 | 7.3 | 18.05 | 0 | 4.6 | 92 | 100.1 | 79.3 |
| 28/10/2015 | 17.1 | 11.6 | 22.9 | 18.6 | 3.1 | 89 | 98.7 | 69.7 |
| 29/10/2015 | 15.5 | 0 | 15.5 | 0.2 | 3.3 | 91 | 100 | 75.3 |
| 30/10/2015 | 13.8 | 12 | 19.8 | 2 | 0.1 | 90 | 97.1 | 82.1 |
| 31/10/2015 | 15.4 | 5.7 | 18.25 | 0.8 | 0.8 | 89 | 100.1 | 75.9 |
| 01/11/2015 | 14.1 | 3.9 | 16.05 | 0.2 | 4.9 | 95 | 100.1 | 78.7 |
| 02/11/2015 | 12 | 7.7 | 15.85 | 0.6 | 0.2 | 100 | 100.1 | 98.6 |
| 03/11/2015 | 14.1 | 7.3 | 17.75 | 0 | 4.4 | 95 | 100.1 | 85.9 |
| 04/11/2015 | 13 | 7.4 | 16.7 | 10.4 | 0.5 | 93 | 100.1 | 82 |
| 05/11/2015 | 14.3 | 11.3 | 19.95 | 1.6 | 1.2 | 94 | 98.5 | 87.4 |
| 06/11/2015 | 14.9 | 12.6 | 21.2 | 8 | 0.1 | 92 | 97.5 | 85.2 |
| 07/11/2015 | 16.2 | 13 | 22.7 | 4.2 | 0 | 93 | 97.7 | 88.8 |
| 08/11/2015 | 16.7 | 6.7 | 20.05 | 2 | 1.1 | 93 | 100.1 | 83.1 |
| 09/11/2015 | 15.8 | 8.6 | 20.1 | 0 | 0.3 | 87 | 96.6 | 78.9 |
| 10/11/2015 | 14.2 | 10.4 | 19.4 | 0 | 1.1 | 83 | 93.7 | 70.7 |
| 11/11/2015 | 15.4 | 12.5 | 21.65 | 1 | 0.3 | 84 | 91 | 73.6 |
| 12/11/2015 | 15.1 | 10.2 | 20.2 | 0 | 0.7 | 83 | 99 | 67.3 |
| 13/11/2015 | 14.7 | 7.5 | 18.45 | 0.2 | 1.8 | 80 | 88.8 | 66.7 |
| 14/11/2015 | 11.8 | 6.2 | 14.9 | 0.2 | 4 | 72 | 83.3 | 51.8 |
| 15/11/2015 | 14.4 | 8.1 | 18.45 | 8 | 0 | 88 | 96.2 | 78.8 |
| 16/11/2015 | 15.2 | 12.3 | 21.35 | 0 | 1.1 | 80 | 92.8 | 70.9 |
| 17/11/2015 | 14 | 10.9 | 19.45 | 3 | 0.6 | 81 | 97.4 | 69.9 |
| 18/11/2015 | 15.2 | 9.6 | 20 | 2.6 | 0.5 | 77 | 95.6 | 62.4 |
| 20/11/2015 | 12.1 | 6.7 | 15.45 | 2.4 | 0 | 90 | 97.1 | 79.4 |
| 21/11/2015 | 8.2 | 0.9 | 8.65 | 2 | 1.5 | 75 | 93.9 | 65.7 |
| 22/11/2015 | 5.1 | -3.5 | 3.35 | 1.6 | 1.7 | 73 | 92.7 | 64.2 |
| 23/11/2015 | 5.3 | -3.2 | 3.7 | 0 | 4.6 | 87 | 99.6 | 64.7 |
| 24/11/2015 | 9.4 | 0.5 | 9.65 | 11.2 | 2.4 | 84 | 97.7 | 68.5 |
| 25/11/2015 | 9.7 | 6.3 | 12.85 | 1 | 1.3 | 87 | 94.8 | 77.3 |
| 26/11/2015 | 9.8 | 2.6 | 11.1 | 0 | 1.6 | 85 | 100 | 60 |
| 27/11/2015 | 10.9 | 5.6 | 13.7 | 0 | 1.6 | 91 | 100 | 76.3 |
| 28/11/2015 | 11.4 | 1.9 | 12.35 | 1 | 1.1 | 86 | 95.5 | 75.9 |
| 29/11/2015 | 10.6 | 3.4 | 12.3 | 0.2 | 2.5 | 76 | 83.9 | 69.8 |
| 30/11/2015 | 12.8 | 7.7 | 16.65 | 2.6 | 0.1 | 77 | 97.1 | 67.7 |
| 01/12/2015 | 12.8 | 11 | 18.3 | 0.2 | 0.1 | 87 | 93.6 | 74.6 |
| 02/12/2015 | 13.8 | 10.2 | 18.9 | 0 | 0.6 | 74 | 86.5 | 64.1 |
| 03/12/2015 | 12.2 | 10.7 | 17.55 | 0.2 | 0.3 | 83 | 90.3 | 73 |
| 04/12/2015 | 12.8 | 4.7 | 15.15 | 7 | 0.9 | 83 | 92.2 | 75.3 |
| 05/12/2015 | 11.8 | 5.7 | 14.65 | 0 | 5.7 | 77 | 82.9 | 67.4 |
| 06/12/2015 | 12.1 | 10.7 | 17.45 | 0.2 | 0 | 76 | 90.9 | 65.3 |
| 07/12/2015 | 13.3 | 10.9 | 18.75 | 1.4 | 0.3 | 92 | 96.4 | 79.8 |
| 08/12/2015 | 12.1 | 9.7 | 16.95 | 6.6 | 1.5 | 89 | 97.1 | 83.7 |
| 09/12/2015 | 12.9 | 1.3 | 13.55 | 1 | 2.1 | 84 | 98.4 | 72 |
| 10/12/2015 | 11.4 | 2.9 | 12.85 | 0.6 | 5.3 | 79 | 91.1 | 69.6 |
| 12/12/2015 | 10.7 | 3.9 | 12.65 | 0.2 | 0.1 | 81 | 88.7 | 69.7 |
| 13/12/2015 | 12.2 | 6 | 15.2 | 1 | 0.1 | 85 | 98.3 | 77.1 |
| 14/12/2015 | 9.6 | 6.2 | 12.7 | 0 | 0 | 93 | 99 | 87.9 |
| 15/12/2015 | 10.3 | 7.5 | 14.05 | 3.8 | 0.6 | 93 | 98.1 | 81.2 |
| 16/12/2015 | 12.8 | 10.3 | 17.95 | 7.4 | 0.2 | 95 | 98.5 | 88.6 |
| 17/12/2015 | 14.6 | 11.3 | 20.25 | 0.2 | 0.1 | 86 | 96.9 | 78.6 |
| 18/12/2015 | 14.6 | 9.8 | 19.5 | 1 | 1.6 | 84 | 95.9 | 67.4 |
| 19/12/2015 | 13.8 | 11.1 | 19.35 | 0 | 0.3 | 88 | 96.3 | 79.6 |
| 20/12/2015 | 15.7 | 11.5 | 21.45 | 0 | 1.2 | 77 | 85.3 | 73 |
| 21/12/2015 | 12.9 | 6.2 | 16 | 5 | 0.4 | 85 | 93.3 | 81.2 |
| 22/12/2015 | 13.5 | 7.8 | 17.4 | 2.8 | 0.9 | 90 | 97.1 | 80.4 |
| 24/12/2015 | 11.8 | 8.1 | 15.85 | 0 | 4.5 | 79 | 90.3 | 64.3 |
| 25/12/2015 | 12.7 | 3.3 | 14.35 | 1.8 | 1.4 | 81 | 98.1 | 67.3 |
| 26/12/2015 | 14 | 8.2 | 18.1 | 2.2 | 0 | 86 | 98 | 80.4 |
| 27/12/2015 | 13.9 | 12.4 | 20.1 | 0 | 0 | 79 | 84.3 | 75.8 |
| 28/12/2015 | 13.4 | 7.2 | 17 | 0 | 0.8 | 89 | 95.6 | 76.8 |
| 29/12/2015 | 12 | 9.2 | 16.6 | 0.4 | 3.7 | 80 | 90 | 69.2 |
| 30/12/2015 | 11.9 | 7.5 | 15.65 | 0.8 | 2.1 | 83 | 92.4 | 69.3 |
| 31/12/2015 | 12.2 | 7.1 | 15.75 | 5.4 | 0.9 | 77 | 94.4 | 66.4 |
| 03/01/2016 | 10.4 | 4.8 | 12.8 | 3 | 0.2 | 89 | 97 | 78.2 |
| 04/01/2016 | 8.2 | 4.2 | 10.3 | 11 | 0.2 | 95 | 97.8 | 89.1 |
| 05/01/2016 | 9 | 5.9 | 11.95 | 7 | 0.1 | 93 | 96.9 | 88.7 |
| 06/01/2016 | 9.4 | 1.6 | 10.2 | 9.4 | 0.7 | 96 | 100 | 88.2 |
| 07/01/2016 | 8 | 3.6 | 9.8 | 10.4 | 0.9 | 94 | 100 | 86.5 |
| 08/01/2016 | 10.1 | -1.9 | 9.15 | 2.8 | 2.5 | 83 | 99.6 | 66.6 |
| 09/01/2016 | 9.6 | -0.6 | 9.3 | 8.4 | 3.9 | 89 | 98.6 | 66.4 |
| 10/01/2016 | 9.9 | 4.6 | 12.2 | 14.6 | 0.1 | 87 | 92.1 | 80.6 |
| 12/01/2016 | 6.9 | 3.4 | 8.6 | 0.8 | 1.3 | 89 | 95.6 | 80.6 |
| 13/01/2016 | 6.8 | 2.6 | 8.1 | 0.8 | 4 | 79 | 87.1 | 67.5 |
| 14/01/2016 | 6.2 | 1.2 | 6.8 | 3.2 | 3.2 | 82 | 94 | 60.9 |
| 15/01/2016 | 6.3 | 0 | 6.3 | 0 | 1.5 | 68 | 86.2 | 54.7 |
| 16/01/2016 | 5.2 | 0.5 | 5.45 | 0 | 6.7 | 73 | 84.8 | 53.8 |
| 17/01/2016 | 4.5 | -1.2 | 3.9 | 0.2 | 2.2 | 83 | 93.3 | 66.8 |
| 19/01/2016 | 3.4 | -5.9 | 0.45 | 0 | 0.9 | 83 | 92.9 | 64.4 |
| 20/01/2016 | 5.8 | -6.3 | 2.65 | 0 | 5.8 | 80 | 92.8 | 37.5 |
| 21/01/2016 | 4.7 | -5 | 2.2 | 0 | 2.3 | 89 | 94.2 | 76 |
| 22/01/2016 | 7.1 | -1.4 | 6.4 | 0.2 | 0.4 | 75 | 94.7 | 63.3 |
| 23/01/2016 | 9.6 | 1.6 | 10.4 | 5.2 | 1.1 | 90 | 95.3 | 78.1 |
| 24/01/2016 | 11.4 | 2.9 | 12.85 | 2.2 | 0.6 | 92 | 96.6 | 86.1 |
| 25/01/2016 | 13.1 | 5.9 | 16.05 | 0 | 0.9 | 91 | 95.4 | 87.4 |
| 26/01/2016 | 13.2 | 5 | 15.7 | 0 | 4.2 | 83 | 94.5 | 68.3 |
| 02/02/2016 | 12.8 | 8.8 | 17.2 | 0 | 0.4 | 71 | 80.9 | 58.7 |
| 03/02/2016 | 10.1 | 1.7 | 10.95 | 0 | 5.3 | 68 | 81.2 | 48.4 |
| 04/02/2016 | 10 | 3.7 | 11.85 | 1 | 2.7 | 73 | 91.3 | 59.8 |
| 05/02/2016 | 13.7 | 8.6 | 18 | 0 | 1.5 | 81 | 90.3 | 67.3 |
| 06/02/2016 | 11.1 | 9.3 | 15.75 | 0.4 | 0.3 | 81 | 86.3 | 75.7 |
| 07/02/2016 | 11.5 | 5.1 | 14.05 | 4 | 0.5 | 78 | 89.1 | 66.4 |
| 08/02/2016 | 10.6 | 6.6 | 13.9 | 8.4 | 4.4 | 74 | 91.5 | 58.2 |
| 09/02/2016 | 9.8 | 4.7 | 12.15 | 1.4 | 0.9 | 69 | 87.8 | 58.2 |
| 12/02/2016 | 8.9 | -2.5 | 7.65 | 0.8 | 8.2 | 81 | 95.1 | 50.6 |
| 13/02/2016 | 5.5 | -1.8 | 4.6 | 0 | 0.1 | 87 | 94.3 | 78.3 |
| 14/02/2016 | 4.5 | 2.5 | 5.75 | 8 | 0 | 89 | 92.4 | 81.3 |
| 15/02/2016 | 6.2 | 0.3 | 6.35 | 0 | 2.2 | 76 | 87 | 54.9 |
| 16/02/2016 | 5.7 | -3.9 | 3.75 | 0 | 3.1 | 82 | 94.6 | 66 |
| 18/02/2016 | 7.4 | 3 | 8.9 | 1.6 | 2.8 | 79 | 92.8 | 56 |
| 19/02/2016 | 7.5 | -3.5 | 5.75 | 0 | 5.6 | 79 | 94.1 | 54 |
| 20/02/2016 | 9.8 | 1.2 | 10.4 | 0.4 | 5 | 81 | 92.8 | 60.9 |
| 21/02/2016 | 12.6 | 8.8 | 17 | 0.6 | 0 | 85 | 92.7 | 70.2 |
| 22/02/2016 | 13.2 | 8 | 17.2 | 2 | 0 | 86 | 92.8 | 80.4 |
| 23/02/2016 | 8.5 | 2.8 | 9.9 | 2.8 | 0.2 | 74 | 93 | 63.7 |
| 24/02/2016 | 9 | -4.1 | 6.95 | 0 | 6.5 | 81 | 94.7 | 56.5 |
| 26/02/2016 | 5.8 | -4 | 3.8 | 0.2 | 3.1 | 81 | 94.2 | 61.7 |
| 27/02/2016 | 5.9 | -1 | 5.4 | 0 | 0.3 | 68 | 91 | 57.1 |
| 28/02/2016 | 5.9 | 2.9 | 7.35 | 0 | 0.5 | 60 | 83.2 | 49.3 |
| 29/02/2016 | 7.4 | 0.9 | 7.85 | 0.2 | 6.5 | 76 | 90.4 | 58.9 |
| 01/03/2016 | 7.9 | 3.3 | 9.55 | 0.8 | 4.8 | 72 | 93 | 51 |
| 02/03/2016 | 11.8 | 3.9 | 13.75 | 4.4 | 2.4 | 81 | 93 | 67.8 |
| 03/03/2016 | 7.1 | 2.9 | 8.55 | 2.6 | 2.9 | 73 | 85.3 | 62.4 |
| 04/03/2016 | 9.6 | 2.1 | 10.65 | 5.4 | 7.2 | 73 | 93 | 47 |
| 05/03/2016 | 8.8 | -2 | 7.8 | 0.6 | 8.1 | 76 | 93.4 | 44 |
| 06/03/2016 | 6.2 | 1.2 | 6.8 | 1.4 | 2.6 | 81 | 88.5 | 69.6 |
| 07/03/2016 | 7.9 | 0.5 | 8.15 | 0 | 6.1 | 73 | 82.7 | 59.5 |
| 08/03/2016 | 6.9 | -3.4 | 5.2 | 0.8 | 5 | 77 | 95.3 | 59.5 |
| 09/03/2016 | 8.7 | 1 | 9.2 | 14.6 | 1.7 | 81 | 95.4 | 54.6 |
| 10/03/2016 | 9.3 | 5.3 | 11.95 | 1.2 | 0.8 | 86 | 91 | 79.5 |
| 11/03/2016 | 9.5 | -1.2 | 8.9 | 0 | 2.7 | 86 | 100.2 | 55.4 |
| 12/03/2016 | 9.4 | -0.1 | 9.35 | 0.2 | 5.8 | 88 | 99.8 | 65.7 |
| 13/03/2016 | 10.8 | 1.1 | 11.35 | 0 | 4.1 | 87 | 98.8 | 59.1 |
| 14/03/2016 | 8.4 | 4.4 | 10.6 | 0 | 3.7 | 78 | 85.7 | 67.3 |
| 15/03/2016 | 7.5 | 2 | 8.5 | 0 | 6.2 | 83 | 97.2 | 67.2 |
| 16/03/2016 | 7.4 | 5.8 | 10.3 | 0.8 | 0.3 | 91 | 96.5 | 84.2 |
| 17/03/2016 | 8.9 | 0.2 | 9 | 0 | 3.8 | 79 | 96.7 | 59.5 |
| 18/03/2016 | 9.5 | 1.8 | 10.4 | 0 | 6.5 | 78 | 92.2 | 51.6 |
| 19/03/2016 | 7 | 3.3 | 8.65 | 0 | 0 | 82 | 90 | 75.6 |
| 20/03/2016 | 8.3 | 4.5 | 10.55 | 0.2 | 0.4 | 80 | 92.8 | 69.4 |
| 21/03/2016 | 10.1 | 5.5 | 12.85 | 0 | 2.5 | 79 | 92.1 | 58.1 |
| 22/03/2016 | 11.4 | -1.1 | 10.85 | 0 | 3.1 | 76 | 99 | 47 |
| 23/03/2016 | 12.7 | 4.2 | 14.8 | 0 | 4.1 | 72 | 88.3 | 47.8 |
| 24/03/2016 | 10.3 | 5.1 | 12.85 | 0 | 0 | 70 | 83.5 | 60.1 |
| 25/03/2016 | 11.2 | 7 | 14.7 | 8.8 | 1 | 87 | 96.8 | 66.9 |
| 27/03/2016 | 13.5 | 5.7 | 16.35 | 6 | 0.5 | 86 | 93.5 | 79.6 |
| 28/03/2016 | 10.6 | 6.4 | 13.8 | 18 | 3.3 | 80 | 92.7 | 64.1 |
| 29/03/2016 | 12.4 | 4.6 | 14.7 | 0.6 | 7.3 | 70 | 89.1 | 41.6 |
| 30/03/2016 | 12.8 | 4.6 | 15.1 | 8.2 | 5.9 | 78 | 90.7 | 49 |
| 31/03/2016 | 13 | 3.6 | 14.8 | 0.2 | 4.7 | 76 | 94.2 | 47.1 |
| 01/04/2016 | 12.4 | -1.5 | 11.65 | 0.4 | 7.5 | 79 | 99.4 | 50.2 |
| 02/04/2016 | 12.4 | 2.6 | 13.7 | 0 | 9.3 | 71 | 94.7 | 53.8 |
| 03/04/2016 | 13.7 | 6.7 | 17.05 | 0.8 | 7 | 78 | 95.9 | 46.5 |
| 04/04/2016 | 16.2 | 7.8 | 20.1 | 4.2 | 5.3 | 82 | 98.5 | 53 |
| 05/04/2016 | 12.9 | 3.8 | 14.8 | 0 | 2.5 | 85 | 98.9 | 65.4 |
| 06/04/2016 | 14.3 | 4.9 | 16.75 | 0.2 | 3.2 | 75 | 93.5 | 56.5 |
| 07/04/2016 | 13.2 | 3.7 | 15.05 | 0.8 | 6.5 | 68 | 93.9 | 44.5 |
| 08/04/2016 | 10.1 | 2.1 | 11.15 | 2 | 2.3 | 84 | 98.4 | 65.2 |
| 09/04/2016 | 13 | 5.4 | 15.7 | 0.4 | 1.8 | 79 | 94.6 | 59.3 |
| 10/04/2016 | 10.2 | -1.6 | 9.4 | 0.2 | 4.3 | 80 | 98 | 55.8 |
| 11/04/2016 | 12.7 | 8 | 16.7 | 0 | 8.4 | 69 | 86 | 33.1 |
| 12/04/2016 | 14.2 | 12.4 | 20.4 | 0 | 2.5 | 83 | 94.5 | 64 |
| 13/04/2016 | 16.8 | 1.7 | 17.65 | 0 | 9.5 | 74 | 99 | 38 |
| 14/04/2016 | 17.2 | 3.5 | 18.95 | 2.6 | 6 | 76 | 98.5 | 41.5 |
| 15/04/2016 | 16.8 | 8.5 | 21.05 | 6 | 5.2 | 78 | 97.6 | 49.3 |
| 16/04/2016 | 13.5 | 5 | 16 | 7.2 | 2.2 | 87 | 94.4 | 77.9 |
| 17/04/2016 | 8.2 | 0.4 | 8.4 | 0 | 5.1 | 78 | 93.7 | 59.8 |
| 18/04/2016 | 11.5 | -0.1 | 11.45 | 0 | 11.3 | 67 | 95.4 | 37.2 |
| 19/04/2016 | 12.3 | 8.5 | 16.55 | 0 | 2 | 65 | 77.8 | 52.1 |
| 20/04/2016 | 14.8 | 0.9 | 15.25 | 0 | 11.5 | 70 | 98.8 | 40.7 |
| 21/04/2016 | 12.3 | 0 | 12.3 | 0 | 11.6 | 75 | 86.6 | 58.2 |
| 22/04/2016 | 14.1 | 8.4 | 18.3 | 0 | 4.8 | 73 | 83.1 | 56.3 |
| 24/04/2016 | 10.4 | 2.8 | 11.8 | 0.4 | 3.7 | 71 | 90.4 | 44.2 |
| 26/04/2016 | 11.7 | 1.7 | 12.55 | 1.8 | 4.5 | 77 | 89.7 | 58.4 |
| 27/04/2016 | 9 | 1.8 | 9.9 | 0 | 6.6 | 67 | 85.8 | 42.9 |
| 29/04/2016 | 11.7 | 5.7 | 14.55 | 0 | 7.2 | 61 | 83.8 | 39.3 |
| 30/04/2016 | 12 | 1.3 | 12.65 | 0.4 | 8.9 | 66 | 89.5 | 39.4 |
| 01/05/2016 | 13.6 | -1.1 | 13.05 | 0 | 9.8 | 68 | 97.1 | 28.7 |
| 02/05/2016 | 15.2 | 8.6 | 19.5 | 0 | 8.2 | 67 | 93.2 | 36.7 |
| 03/05/2016 | 14.7 | 5.3 | 17.35 | 0.2 | 5.3 | 77 | 90.2 | 57.2 |
| 04/05/2016 | 16.4 | 2.1 | 17.45 | 0 | 12.1 | 64 | 98.8 | 34.3 |
| 05/05/2016 | 16.9 | 2.2 | 18 | 0 | 14.5 | 59 | 96.5 | 22.4 |
| 06/05/2016 | 19.2 | 5.1 | 21.75 | 0 | 11.7 | 59 | 96.9 | 27.9 |
| 07/05/2016 | 22.8 | 9 | 27.3 | 0 | 11.8 | 65 | 97.5 | 31.8 |
| 08/05/2016 | 23.2 | 7.4 | 26.9 | 0 | 8.5 | 65 | 95.6 | 38.6 |
| 09/05/2016 | 25.3 | 11.1 | 30.85 | 0 | 10.6 | 52 | 82.9 | 30.4 |
| 10/05/2016 | 23.4 | 14.5 | 30.65 | 4.6 | 4.2 | 77 | 96.3 | 47.5 |
| 11/05/2016 | 18.7 | 13.7 | 25.55 | 9.8 | 0.1 | 93 | 98.9 | 78.7 |
| 12/05/2016 | 20.2 | 12.6 | 26.5 | 3.6 | 4.9 | 86 | 97.3 | 72.5 |
| 13/05/2016 | 20 | 11 | 25.5 | 0 | 9.4 | 75 | 89.4 | 63.3 |
| 14/05/2016 | 20.5 | 7 | 24 | 0 | 6.9 | 64 | 74.6 | 50 |
| 15/05/2016 | 12.6 | 1.1 | 13.15 | 0 | 7.4 | 72 | 99.3 | 47.9 |
| 16/05/2016 | 17.9 | 8.3 | 22.05 | 0 | 6.7 | 67 | 93.2 | 35.4 |
| 17/05/2016 | 17.7 | 4.5 | 19.95 | 0 | 9.5 | 77 | 99.4 | 54 |
| 18/05/2016 | 18.2 | 10.4 | 23.4 | 0 | 3.8 | 71 | 87.7 | 44.3 |
| 19/05/2016 | 14.3 | 10.5 | 19.55 | 8 | 0.8 | 88 | 94.9 | 74.5 |
| 20/05/2016 | 17.8 | 11.9 | 23.75 | 0 | 4.7 | 78 | 93.9 | 54.7 |
| 21/05/2016 | 19 | 11.1 | 24.55 | 0 | 4.5 | 74 | 88.9 | 56.9 |
| 22/05/2016 | 17.8 | 11.8 | 23.7 | 0.8 | 1.4 | 82 | 96.1 | 68.8 |
| 23/05/2016 | 18.2 | 10.7 | 23.55 | 7.2 | 1.9 | 76 | 94.8 | 51 |
| 25/05/2016 | 16.1 | 8.8 | 20.5 | 0 | 7 | 69 | 80.3 | 58.3 |
| 26/05/2016 | 14.4 | 3.1 | 15.95 | 0 | 3.7 | 86 | 99.6 | 65.3 |
| 27/05/2016 | 18.4 | 10.5 | 23.65 | 0 | 7.2 | 73 | 87.9 | 52.5 |
| 28/05/2016 | 18.8 | 11.5 | 24.55 | 0.4 | 9.4 | 80 | 91.5 | 64.5 |
| 29/05/2016 | 18.3 | 9.3 | 22.95 | 0 | 7.2 | 77 | 89.5 | 63.3 |
| 30/05/2016 | 20.4 | 11.6 | 26.2 | 0 | 6.6 | 77 | 87.3 | 64.3 |
| 31/05/2016 | 15.5 | 10.5 | 20.75 | 37.4 | 0.1 | 81 | 95.3 | 72 |
| 01/06/2016 | 12.7 | 10.9 | 18.15 | 3 | 0 | 93 | 97.3 | 85.4 |
| 02/06/2016 | 12.5 | 10 | 17.5 | 7 | 0 | 94 | 96.2 | 87.4 |
| 03/06/2016 | 13.2 | 9.2 | 17.8 | 0 | 2.9 | 84 | 90 | 76.4 |
| 04/06/2016 | 13.7 | 10.1 | 18.75 | 0 | 1.2 | 91 | 98.4 | 81.8 |
| 05/06/2016 | 18.4 | 11.6 | 24.2 | 0 | 14.5 | 87 | 95.9 | 80 |
| 06/06/2016 | 20.6 | 10.4 | 25.8 | 0 | 12.3 | 79 | 94 | 61.4 |

**Table S3: Environmental data for the field experiment conducted October 2015-June 2016.** This was obtained using the NIAB EMR weather station. For each date, the max/min temperature, mean temperature, mean rainfall, sunshine hours, % relative humidity and max/min humidity values are listed.
