# Supplementary material for: Characterization of the pathogenicity of strains of Pseudomonas syringae towards cherry and plum
Source: Plant Pathol. 2018 Feb 14;67(5):1177–93. doi: 10.1111/ppa.12834 (PMC5993217; doi:10.1111/ppa.12834)
Supplement: Supplementary file 12 — Table S4. Genome assembly statistics for all Pseudomonas syringae strains sequenced in this study. [file PPA-67-1177-s012.docx]

| **Assembly** | **no. contigs** | **Total length** | **GC%** | **N50** | **Average coverage** | **Features** |
| --- | --- | --- | --- | --- | --- | --- |
| R1-5244 | 198 | 6302385 | 58.08 | 227422 | 265 | 5810 |
| R1-5300 | 201 | 6342586 | 57.88 | 142021 | 180 | 5844 |
| R1-9326 | 268 | 6353636 | 57.91 | 142021 | 81 | 5874 |
| R1-9629 | 216 | 6341664 | 57.94 | 142021 | 172 | 5856 |
| R1-9646 | 171 | 6302776 | 58.03 | 235429 | 180 | 5801 |
| R1-9657 | 191 | 6317852 | 57.91 | 145272 | 158 | 5848 |
| R2-5255 | 206 | 6448834 | 58.38 | 102760 | 112 | 5966 |
| R2-5260 | 223 | 6495620 | 58.41 | 101794 | 458 | 5995 |
| R2-leaf | 203 | 6366714 | 58.48 | 100658 | 180 | 5846 |
| R2-sc214 | 203 | 6253818 | 58.56 | 108341 | 180 | 5747 |
| *Ps* 9643 | 58 | 5937102 | 58.78 | 243355 | 212 | 5386 |
| *Pss* 9097 | 66 | 5892389 | 59.35 | 316078 | 158 | 5117 |
| *Pss* 9293 | 73 | 6135031 | 58.84 | 557853 | 196 | 5302 |
| *Pss* 9630 | 57 | 5940819 | 59.33 | 347701 | 206 | 5175 |
| *Pss* 9644 | 75 | 6173193 | 59.13 | 251053 | 208 | 5334 |
| *Pss* 9654 | 49 | 5941610 | 59.37 | 245023 | 147 | 5148 |
| *Pss* 9656 | 39 | 5980728 | 59.10 | 1007808 | 205 | 5184 |
| *Pss* 9659 | 51 | 5943090 | 59.37 | 235830 | 116 | 5148 |
| RMA1 | 95 | 6306889 | 58.73 | 187448 | 320 | 5825 |

**Table S4: Genome assembly statistics for all *P. syringae* strains sequenced in this study.** The table lists the number of contigs in each genome assembly, the total length, %GC cotent, the N50 and average coverage and number of annotated features (protein-encoding and RNA genes).
