# Supplementary material for: Characterization of the pathogenicity of strains of Pseudomonas syringae towards cherry and plum
Source: Plant Pathol. 2018 Feb 14;67(5):1177–93. doi: 10.1111/ppa.12834 (PMC5993217; doi:10.1111/ppa.12834)
Supplement: Supplementary file 13 — Table S5. Proportional odds model (POM) analysis of the glasshouse whole‐tree wound inoculations. [file PPA-67-1177-s013.docx]

| **Optimising models** | | | | | | | | | |
| --- | --- | --- | --- | --- | --- | --- | --- | --- | --- |
|  | **formula:** | **link:** | **threshold:** |  |  | |  | |  |
| fm1 | score ~1 | logit | flexible |  |  | |  | |  |
| fm3 | score ~g1 | logit | flexible |  |  | |  | |  |
| fm2 | score ~strain | logit | flexible |  |  | |  | |  |
|  |  |  |  |  |  | |  | |  |
|  | **no.par** | **AIC** | **logLik** | **LR.stat** | **df** | | **Pr(>Chisq)** | |  |
| fm1 | 3 | 305.58 | -149.79 |  |  | |  | |  |
| fm3 | 10 | 135.94 | -56.97 | 183.64 | 7 | | <2e-16 | | *** |
| fm2 | 24 | 154.85 | -53.43 | 7.09 | 14 | | 0.93 | |  |
| **Coefficients**: | |  |  |  |  | |  | |  |
|  | Estimate | Std. | Error | z value | Pr(>\|z\|) | |  | |  |
| g12 | -2.04 | 0.10 | -2.08 | 0.04 | * | |  | |  |
| g13 | -5.65 | 1.30 | -4.34 | 1.45E-05 | *** | |  | |  |
| g14 | -5.64 | 2.08 | -2.71 | 0.01 | ** | |  | |  |
| g15 | -9.68 | 1.78 | -5.43 | 5.67E-08 | *** | |  | |  |
| g16 | -11.25 | 1.71 | -6.57 | 5.00E-11 | *** | |  | |  |
| g17 | -13.78 | 1.85 | -7.45 | 9.15E-14 | *** | |  | |  |
| g18 | -9.29 | 1.90 | -4.89 | 1.04E-06 | *** | |  | |  |
| **Groups** |  |  |  |  | |  | |  |  |
| g1 | lsmean | SE | df | asymp.LCL | | asymp.UCL | | Group |  |
| 7 | -6.15 | 0.97 | NA | -8.05 | | -4.26 | | 1 |  |
| 6 | -3.63 | 0.70 | NA | -5.01 | | -2.25 | | 12 |  |
| 5 | -2.06 | 0.94 | NA | -3.89 | | -0.22 | | 23 |  |
| 8 | -1.66 | 1.16 | NA | -3.94 | | 0.61 | | 23 |  |
| 3 | 1.97 | 0.87 | NA | 0.26 | | 3.69 | | 34 |  |
| 4 | 1.98 | 1.85 | NA | -1.63 | | 5.60 | | 2345 |  |
| 2 | 5.59 | 1.15 | NA | 3.34 | | 7.84 | | 45 |  |
| 1 | 7.62 | 1.20 | NA | 5.26 | | 9.98 | | 5 |  |
| \| **Strains in each grouping** \| \| \| \| \| --- \| --- \| --- \| --- \| \| 1: *Pss*-9097, *Pss*-9644, *Pss*-9654, *Pss*-9659, *Pss*-9630 \| \| \| \| \| 2: *Pss*-9656, R1-9646 \| \|  \|  \| \| 3: R1-5244, R2-5255, R2-leaf, R2-SC214 \| \| \|  \| \| 4: *Pss*-9293 \|  \|  \|  \| \| 5: R1-9657 \|  \|  \|  \| \| 6: Ps-9643, *Psv*, R1-9326, R1-9629 \| \| \|  \| \| 7: Control, *Pph*, R1-5300, RMA1 \| \| \|  \| \| 8: R2-5260 \|  \|  \|  \| | | | | | | | |  |  |

**Table S5: Proportional Odds Model (POM) analysis of the glasshouse whole-tree wound inoculations.** Model comparisons are first shown with the ANOVA comparing models. The summary of the final model (score ~g1) is shown along with lsmeans and Tukey-HSD groupings of strains (corresponds to groupings on Figure 2).
