# Supplementary material for: Characterization of the pathogenicity of strains of Pseudomonas syringae towards cherry and plum
Source: Plant Pathol. 2018 Feb 14;67(5):1177–93. doi: 10.1111/ppa.12834 (PMC5993217; doi:10.1111/ppa.12834)
Supplement: Supplementary file 14 — Table S6. Proportional odds model (POM) analysis of the cherry field inoculations. [file PPA-67-1177-s014.docx]

| **Optimising models** | | | | | | | | |
| --- | --- | --- | --- | --- | --- | --- | --- | --- |
|  | | **formula:** | | | | **link:** | | **threshold:** |
| fm1 | | score ~ 1 | | | | logit | | flexible |
| fm2 | | score ~ strain + block | | | | logit | | flexible |
| fm3 | | score ~ strain + cv + block | | | | logit | | flexible |
| fm4 | | score ~ strain + cv + ino + block | | | | logit | | flexible |
| fm7 | | score ~ strain + cv * ino + block | | | | logit | | flexible |
| fm6 | | score ~ strain * ino + cv + block | | | | logit | | flexible |
| fm5 | | score ~ strain * cv + ino + block | | | | logit | | flexible |
|  | **no.par** | | **AIC** | **logLik** | **LR.stat** | **df** | **Pr(>Chisq)** |  |
| fm1 | 3 | | 1637.3 | -815.63 |  |  |  |  |
| fm2 | 20 | | 1446.4 | -703.18 | 224.9 | 17 | <2.20E-16 | *** |
| fm3 | 23 | | 1427.7 | -690.87 | 24.63 | 3 | 1.85E-05 | *** |
| fm4 | 24 | | 1293.2 | -622.58 | 136.59 | 1 | <2.20E-16 | *** |
| fm7 | 27 | | 1292.3 | -619.13 | 6.9 | 3 | 0.08 | . |
| fm6 | 32 | | 1302 | -618.99 | 0.28 | 5 | 0.1 |  |
| fm5 | 48 | | 1321.3 | -612.64 | 12.69 | 16 | 0.7 |  |

| **Final model: clm(score~strain+cv+ino + block)** | | | | | | | | | |  |
| --- | --- | --- | --- | --- | --- | --- | --- | --- | --- | --- |
| link | threshold | | nobs | | logLik | | AIC | niter | max.grad | cond.H |
| logit | flexible | | 658 | | -622.58 | | 1293.15 | 6(0) | 1.86E-13 | 2.6E+02 |
|  | |  | |  | |  |  |  |  |  |
| **Coefficients:** | | | | | | | |  |  |  |
|  | | Estimate | | Std. | | Error | z value | Pr(>\|z\|) |  |  |
| strain*Pph* | | 0.56 | | 0.34 | | 1.64 | 0.1 |  |  |  |
| strain*Ps*-9643 | | 0.49 | | 0.33 | | 1.5 | 0.13 |  |  |  |
| strain*Pss*-9097 | | 3.88 | | 0.35 | | 11.05 | <2.00E-16 | *** |  |  |
| strain*Pss*-9293 | | 2.22 | | 0.32 | | 6.9 | 5.05E-12 | *** |  |  |
| strainR1-5244 | | 3.27 | | 0.33 | | 9.83 | <2.00E-16 | *** |  |  |
| strainR1-5300 | | 1.04 | | 0.32 | | 3.28 | 0.001 | ** |  |  |
| strainR2-leaf | | 2.52 | | 0.32 | | 7.87 | 3.61E-15 | *** |  |  |
| strainRMA1 | | 0.07 | | 0.36 | | 0.2 | 0.84 |  |  |  |
| cvnapoleon | | 0.82 | | 0.22 | | 3.67 | 0.0002 | *** |  |  |
| cvroundel | | 0.83 | | 0.24 | | 3.52 | 0.0004 | *** |  |  |
| cvvan | | -0.08 | | 0.23 | | -0.34 | 0.73 |  |  |  |
| inowound | | 1.98 | | 0.18 | | 10.94 | <2.00E-16 | *** |  |  |
| blockB | | 0.23 | | 0.35 | | 0.68 | 0.5 |  |  |  |
| blockC | | 0.44 | | 0.35 | | 1.24 | 0.21 |  |  |  |
| blockD | | -0.34 | | 0.37 | | -0.93 | 0.35 |  |  |  |
| blockE | | 0.12 | | 0.35 | | 0.33 | 0.74 |  |  |  |
| blockF | | -0.77 | | 0.39 | | -1.99 | 0.05 | * |  |  |
| blockG | | 0.03 | | 0.35 | | 0.09 | 0.93 |  |  |  |
| blockH | | -0.35 | | 0.38 | | -0.91 | 0.36 |  |  |  |
| blockI | | -0.1 | | 0.34 | | -0.28 | 0.78 |  |  |  |
| blockJ | | -0.23 | | 0.38 | | -0.61 | 0.54 |  |  |  |

**Table S6: POM analysis of the cherry field inoculations.** Model comparisons are first shown with the ANOVA comparing models. The summary of the final model (score~strain+cv+ino+block) is then presented.
