# Supplementary material for: Characterization of the pathogenicity of strains of Pseudomonas syringae towards cherry and plum
Source: Plant Pathol. 2018 Feb 14;67(5):1177–93. doi: 10.1111/ppa.12834 (PMC5993217; doi:10.1111/ppa.12834)
Supplement: Supplementary file 15 — Table S7. Lsmean Tukey‐HSD groupings for different treatment combinations from the proportional odds model (POM) analysis of cherry field inoculations. [file PPA-67-1177-s015.docx]

| **Lsmeans (strains on different cultivars with different inoculation methods)** | | | | | | |
| --- | --- | --- | --- | --- | --- | --- |
| **Merton Glory scar inoculation** | | |  |  |  |  |
| strain | lsmean | SE | df | asymp.LCL | asymp.UCL | .group |
| Control | -3.87 | 0.3 | NA | -4.46 | -3.27 | 1 |
| RMA1 | -3.79 | 0.37 | NA | -4.52 | -3.06 | 12 |
| *Ps*-9643 | -3.38 | 0.33 | NA | -4.03 | -2.72 | 12 |
| *Pph* | -3.31 | 0.34 | NA | -3.97 | -2.64 | 12 |
| R1-5300 | -2.82 | 0.31 | NA | -3.44 | -2.21 | 2 |
| *Pss*-9293 | -1.65 | 0.29 | NA | -2.22 | -1.07 | 3 |
| R2-leaf | -1.34 | 0.29 | NA | -1.91 | -0.78 | 34 |
| R1-5244 | -0.6 | 0.29 | NA | -1.16 | -0.03 | 45 |
| *Pss*-9097 | 0.01 | 0.29 | NA | -0.57 | 0.59 | 5 |
| **Merton Glory wound inoculation** | | | | | | |
| strain | lsmean | SE | df | asymp.LCL | asymp.UCL | .group |
| Control | -1.88 | 0.25 | NA | -2.38 | -1.39 | 1 |
| RMA1 | -1.81 | 0.33 | NA | -2.47 | -1.16 | 12 |
| *Ps*-9643 | -1.39 | 0.3 | NA | -1.98 | -0.81 | 12 |
| *Pph* | -1.33 | 0.31 | NA | -1.93 | -0.72 | 12 |
| R1-5300 | -0.84 | 0.29 | NA | -1.41 | -0.28 | 2 |
| *Pss*-9293 | 0.34 | 0.28 | NA | -0.21 | 0.88 | 3 |
| R2-leaf | 0.64 | 0.28 | NA | 0.09 | 1.18 | 34 |
| R1-5244 | 1.39 | 0.29 | NA | 0.82 | 1.95 | 45 |
| *Pss*-9097 | 1.99 | 0.3 | NA | 1.4 | 2.58 | 5 |
| **Napoleon scar inoculation** | | | | | | |
| strain | lsmean | SE | df | asymp.LCL | asymp.UCL | .group |
| Control | -3.05 | 0.29 | NA | -3.61 | -2.48 | 1 |
| RMA1 | -2.98 | 0.37 | NA | -3.69 | -2.26 | 12 |
| *Ps*-9643 | -2.56 | 0.32 | NA | -3.19 | -1.93 | 12 |
| *Pph* | -2.49 | 0.33 | NA | -3.13 | -1.85 | 12 |
| R1-5300 | -2 | 0.3 | NA | -2.59 | -1.42 | 2 |
| *Pss*-9293 | -0.83 | 0.29 | NA | -1.4 | -0.26 | 3 |
| R2-leaf | -0.53 | 0.28 | NA | -1.08 | 0.03 | 34 |
| R1-5244 | 0.22 | 0.29 | NA | -0.34 | 0.79 | 45 |
| *Pss*-9097 | 0.83 | 0.3 | NA | 0.23 | 1.42 | 5 |
| **Napoleon wound inoculation** | | | | | | |
| strain | lsmean | SE | df | asymp.LCL | asymp.UCL | .group |
| Control | -1.07 | 0.25 | NA | -1.56 | -0.57 | 1 |
| RMA1 | -1 | 0.34 | NA | -1.66 | -0.33 | 12 |
| *Ps*-9643 | -0.58 | 0.3 | NA | -1.16 | 0 | 12 |
| *Pph* | -0.51 | 0.31 | NA | -1.11 | 0.09 | 12 |
| R1-5300 | -0.02 | 0.28 | NA | -0.57 | 0.52 | 2 |
| *Pss*-9293 | 1.15 | 0.29 | NA | 0.59 | 1.72 | 3 |
| R2-leaf | 1.46 | 0.28 | NA | 0.9 | 2.01 | 34 |
| R1-5244 | 2.2 | 0.3 | NA | 1.61 | 2.79 | 45 |
| *Pss*-9097 | 2.81 | 0.32 | NA | 2.18 | 3.44 | 5 |
| **Roundel scar inoculation** | | | | | | |
| strain | lsmean | SE | df | asymp.LCL | asymp.UCL | .group |
| Control | -3.03 | 0.3 | NA | -3.61 | -2.45 | 1 |
| RMA1 | -2.96 | 0.37 | NA | -3.69 | -2.24 | 12 |
| *Ps*-9643 | -2.54 | 0.33 | NA | -3.19 | -1.9 | 12 |
| *Pph* | -2.48 | 0.34 | NA | -3.14 | -1.81 | 12 |
| R1-5300 | -1.99 | 0.31 | NA | -2.6 | -1.38 | 2 |
| *Pss*-9293 | -0.81 | 0.3 | NA | -1.4 | -0.23 | 3 |
| R2-leaf | -0.51 | 0.3 | NA | -1.09 | 0.07 | 34 |
| R1-5244 | 0.24 | 0.3 | NA | -0.36 | 0.83 | 45 |
| *Pss*-9097 | 0.84 | 0.31 | NA | 0.24 | 1.45 | 5 |
| **Roundel wound inoculation** | | | | | | |
| strain | lsmean | SE | df | asymp.LCL | asymp.UCL | .group |
| Control | -1.05 | 0.26 | NA | -1.57 | -0.54 | 1 |
| RMA1 | -0.98 | 0.34 | NA | -1.65 | -0.31 | 12 |
| *Ps*-9643 | -0.56 | 0.3 | NA | -1.16 | 0.03 | 12 |
| *Pph* | -0.5 | 0.32 | NA | -1.13 | 0.13 | 12 |
| R1-5300 | -0.01 | 0.3 | NA | -0.59 | 0.57 | 2 |
| *Pss*-9293 | 1.17 | 0.3 | NA | 0.59 | 1.75 | 3 |
| R2-leaf | 1.47 | 0.3 | NA | 0.89 | 2.05 | 34 |
| R1-5244 | 2.22 | 0.31 | NA | 1.6 | 2.83 | 45 |
| *Pss*-9097 | 2.82 | 0.33 | NA | 2.18 | 3.46 | 5 |
| **Van scar inoculation** | | | | | | |
| strain | lsmean | SE | df | asymp.LCL | asymp.UCL | .group |
| Control | -3.94 | 0.31 | NA | -4.56 | -3.33 | 1 |
| RMA1 | -3.87 | 0.38 | NA | -4.63 | -3.12 | 12 |
| *Ps*-9643 | -3.45 | 0.34 | NA | -4.12 | -2.79 | 12 |
| *Pph* | -3.39 | 0.35 | NA | -4.07 | -2.7 | 12 |
| R1-5300 | -2.9 | 0.32 | NA | -3.52 | -2.28 | 2 |
| *Pss*-9293 | -1.72 | 0.3 | NA | -2.32 | -1.13 | 3 |
| R2-leaf | -1.42 | 0.3 | NA | -2 | -0.84 | 34 |
| R1-5244 | -0.67 | 0.29 | NA | -1.24 | -0.11 | 45 |
| *Pss*-9097 | -0.07 | 0.31 | NA | -0.67 | 0.53 | 5 |
| **Van wound inoculation** | | | | | | |
| strain | lsmean | SE | df | asymp.LCL | asymp.UCL | .group |
| Control | -1.96 | 0.27 | NA | -2.49 | -1.44 | 1 |
| RMA1 | -1.89 | 0.35 | NA | -2.58 | -1.21 | 12 |
| *Ps*-9643 | -1.47 | 0.31 | NA | -2.07 | -0.87 | 12 |
| *Pph* | -1.41 | 0.32 | NA | -2.04 | -0.77 | 12 |
| R1-5300 | -0.92 | 0.29 | NA | -1.49 | -0.35 | 2 |
| *Pss*-9293 | 0.26 | 0.29 | NA | -0.31 | 0.83 | 3 |
| R2-leaf | 0.56 | 0.29 | NA | 0 | 1.12 | 34 |
| R1-5244 | 1.31 | 0.29 | NA | 0.74 | 1.88 | 45 |
| *Pss*-9097 | 1.91 | 0.32 | NA | 1.29 | 2.53 | 5 |
| **Lsmeans (cultivars with different inoculation methods)** | | | | | | |
| **Scar inoculation** | | | | | | |
| cv | lsmean | SE | df | asymp.LCL | asymp.UCL | .group |
| mglory | -2.3 | 0.2 | NA | -2.7 | -1.9 | 1 |
| van | -2.38 | 0.22 | NA | -2.81 | -1.96 | 1 |
| napoleon | -1.49 | 0.19 | NA | -1.87 | -1.11 | 2 |
| roundel | -1.47 | 0.21 | NA | -1.88 | -1.06 | 2 |
| **Wound inoculation** | | | | | | |
| cv | lsmean | SE | df | asymp.LCL | asymp.UCL | .group |
| mglory | -0.32 | 0.17 | NA | -0.65 | -0.01 | 1 |
| van | -0.4 | 0.19 | NA | -0.77 | 0.04 | 1 |
| napoleon | 0.49 | 0.17 | NA | 0.15 | 0.84 | 2 |
| roundel | 0.51 | 0.19 | NA | 0.13 | 0.89 | 2 |
| **Lsmeans (strains with different inoculation methods)** | | | | | | |
| **Scar inoculation** | | | | | | |
| strain | lsmean | SE | df | asymp.LCL | asymp.UCL | .group |
| Control | -3.53 | 0.27 | NA | -4.05 | -3.01 | 1 |
| RMA1 | -3.45 | 0.35 | NA | -4.13 | -2.77 | 12 |
| *Ps*-9643 | -3.03 | 0.3 | NA | -3.62 | -2.43 | 12 |
| *Pph* | -2.97 | 0.31 | NA | -3.58 | -2.36 | 12 |
| R1-5300 | -2.47 | 0.28 | NA | -3.02 | -1.93 | 2 |
| *Pss*-9293 | -1.28 | 0.26 | NA | -1.79 | -0.77 | 3 |
| R2-leaf | -1.02 | 0.26 | NA | -1.52 | -0.51 | 34 |
| R1-5244 | -0.23 | 0.25 | NA | -0.73 | 0.27 | 45 |
| *Pss*-9097 | 0.35 | 0.27 | NA | -0.17 | 0.88 | 5 |
| **Wound inoculation** | | | | | | |
| strain | lsmean | SE | df | asymp.LCL | asymp.UCL | .group |
| Control | -1.48 | 0.22 | NA | -1.91 | -1.06 | 1 |
| RMA1 | -1.4 | 0.31 | NA | -2.01 | -0.8 | 12 |
| *Ps*-9643 | -0.98 | 0.27 | NA | -1.5 | -0.46 | 12 |
| *Pph* | -0.93 | 0.28 | NA | -1.48 | -0.38 | 12 |
| R1-5300 | -0.43 | 0.25 | NA | -0.93 | 0.06 | 2 |
| *Pss*-9293 | 0.76 | 0.25 | NA | 0.27 | 1.25 | 3 |
| R2-leaf | 1.03 | 0.25 | NA | 0.54 | 1.52 | 34 |
| R1-5244 | 1.82 | 0.26 | NA | 1.3 | 2.33 | 45 |
| *Pss*-9097 | 2.39 | 0.28 | NA | 1.84 | 2.95 | 5 |

**Table S7: Lsmean Tukey-HSD groupings for different treatment combinations from the POM analysis of cherry field inoculations.** Groups for strains on different cultivars per inoculation method are presented, followed by groupings of cultivars in each inoculation method and then strains across the two inoculation methods. This table corresponds to the statistical groupings seen in Figure 3A1/B1.
