# Supplementary material for: Characterization of the pathogenicity of strains of Pseudomonas syringae towards cherry and plum
Source: Plant Pathol. 2018 Feb 14;67(5):1177–93. doi: 10.1111/ppa.12834 (PMC5993217; doi:10.1111/ppa.12834)
Supplement: Supplementary file 16 — Table S8. REML analysis of field inoculation of cherry inoculated by leaf scar. [file PPA-67-1177-s016.docx]

|  |  |  |  |  |  |  |  |
| --- | --- | --- | --- | --- | --- | --- | --- |
| **Model: lmer(log2(length+1) ~ cv * strain + (1\|block/no.))** | | | | | | | |
|  |  |  |  |  |  |  |  |
| **REML criterion at convergence: 1147.276** | | | |  |  |  |  |
| Random effects: | |  |  |  |  |  |  |
| Groups | Name | Std.Dev. |  |  |  |  |  |
| no.:block | (Intercept) | 0.4094 |  |  |  |  |  |
| block | (Intercept) | 0.2575 |  |  |  |  |  |
| Residual | 1.4777 |  |  |  |  |  |  |
|  |  |  |  |  |  |  |  |
| **ANOVA** |  |  |  |  |  |  |  |
|  | Sum Sq | Mean Sq | NumDF | DenDF | F.value | Pr(>F) |  |
| cv | 28.081 | 9.36 | 3 | 54.647 | 4.2866 | 0.008676 | ** |
| strain | 291.283 | 36.41 | 8 | 257.493 | 16.6742 | <2.20E-16 | *** |
| cv:strain | 51.668 | 2.153 | 24 | 256.549 | 0.9859 | 0.485315 |  |
| **Lsmeans Cultivar** | | | |  |  |  |  |
| Cultivar | lsmean | SE | df | lower.CL | upper.CL | .group |  |
| Van | 1.43 | 0.21 | 42.41 | 1.02 | 1.85 | 1 |  |
| Mglory | 1.51 | 0.2 | 39.34 | 1.1 | 1.92 | 1 |  |
| Roundel | 2.01 | 0.23 | 45.71 | 1.56 | 2.47 | 12 |  |
| Napoleon | 2.29 | 0.22 | 54.81 | 1.85 | 2.74 | 2 |  |
| **Lsmeans Strain** | |  |  |  |  |  |  |
| strain | lsmean | SE | df | lower.CL | upper.CL | .group |  |
| RMA1 | 0.83 | 0.32 | 189.53 | 0.2 | 1.47 | 12 |  |
| Control | 0.89 | 0.21 | 65.15 | 0.48 | 1.31 | 1 |  |
| *Pph* | 0.97 | 0.29 | 157.81 | 0.41 | 1.54 | 12 |  |
| *Ps*-9643 | 1.02 | 0.28 | 149.78 | 0.46 | 1.57 | 12 |  |
| R1-5300 | 1.35 | 0.27 | 138.32 | 0.82 | 1.88 | 123 |  |
| *Pss*-9293 | 2.1 | 0.29 | 159.99 | 1.53 | 2.68 | 234 |  |
| R2-leaf | 2.46 | 0.28 | 149.22 | 1.91 | 3.02 | 345 |  |
| R1-5244 | 3.08 | 0.28 | 148.77 | 2.53 | 3.64 | 45 |  |
| *Pss*-9097 | 3.6 | 0.28 | 149.16 | 3.05 | 4.15 | 5 |  |
| **Lsmeans Strain x Cultivars** | | | | | | |  |
| **Merton Glory** | |  |  |  |  |  |  |
| strain | lsmean | SE | df | lower.CL | upper.CL | .group |  |
| *Pph* | 0.62 | 0.49 | 278.69 | -0.34 | 1.59 | 1 |  |
| *Ps*-9643 | 0.85 | 0.49 | 278.65 | -0.11 | 1.81 | 1 |  |
| Control | 1.03 | 0.37 | 234.76 | 0.3 | 1.76 | 1 |  |
| RMA1 | 1.1 | 0.49 | 278.68 | 0.14 | 2.06 | 12 |  |
| *Pss*-9293 | 1.4 | 0.52 | 281.3 | 0.38 | 2.41 | 12 |  |
| R1-5300 | 1.57 | 0.55 | 283.19 | 0.49 | 2.64 | 12 |  |
| R2-leaf | 1.81 | 0.52 | 280.98 | 0.79 | 2.82 | 12 |  |
| R1-5244 | 2.16 | 0.55 | 283.15 | 1.08 | 3.23 | 12 |  |
| *Pss*-9097 | 3.07 | 0.52 | 280.99 | 2.06 | 4.09 | 2 |  |
|  |  |  |  |  |  |  |  |
| **Napoleon** |  |  |  |  |  |  |  |
| strain | lsmean | SE | df | lower.CL | upper.CL | .group |  |
| RMA1 | 0.71 | 0.77 | 287.65 | -0.8 | 2.22 | 1 |  |
| Control | 0.97 | 0.4 | 252.55 | 0.17 | 1.76 | 1 |  |
| *Ps*-9643 | 1.17 | 0.63 | 286.81 | -0.07 | 2.41 | 1 |  |
| *Pph* | 1.18 | 0.55 | 283.54 | 0.11 | 2.26 | 1 |  |
| R1-5300 | 2.02 | 0.49 | 277.92 | 1.06 | 2.99 | 1 |  |
| R2-leaf | 2.54 | 0.49 | 278.54 | 1.57 | 3.5 | 12 |  |
| *Pss*-9293 | 2.79 | 0.63 | 286.91 | 1.55 | 4.03 | 123 |  |
| R1-5244 | 4.44 | 0.52 | 280.63 | 3.42 | 5.45 | 23 |  |
| *Pss*-9097 | 4.83 | 0.55 | 283.38 | 3.75 | 5.91 | 3 |  |
|  |  |  |  |  |  |  |  |
| **Roundel** |  |  |  |  |  |  |  |
| strain | lsmean | SE | df | lower.CL | upper.CL | .group |  |
| RMA1 | 0.75 | 0.63 | 286.39 | -0.49 | 1.99 | 12 |  |
| Control | 0.78 | 0.42 | 250.14 | -0.05 | 1.6 | 1 |  |
| *Ps*-9643 | 1.03 | 0.55 | 282.72 | -0.05 | 2.1 | 123 |  |
| *Pph* | 1.2 | 0.63 | 286.68 | -0.04 | 2.44 | 1234 |  |
| R1-5300 | 1.42 | 0.55 | 282.67 | 0.34 | 2.5 | 1234 |  |
| *Pss*-9293 | 2.92 | 0.55 | 283 | 1.84 | 4 | 234 |  |
| R1-5244 | 3 | 0.58 | 284.81 | 1.85 | 4.15 | 234 |  |
| *Pss*-9097 | 3.49 | 0.55 | 283.22 | 2.41 | 4.56 | 4 |  |
| R2-leaf | 3.53 | 0.63 | 286.41 | 2.29 | 4.77 | 34 |  |
|  |  |  |  |  |  |  |  |
| **Van** |  |  |  |  |  |  |  |
| strain | lsmean | SE | df | lower.CL | upper.CL | .group |  |
| R1-5300 | 0.4 | 0.49 | 278.68 | -0.57 | 1.36 | 1 |  |
| RMA1 | 0.78 | 0.58 | 285.19 | -0.37 | 1.92 | 12 |  |
| Control | 0.79 | 0.37 | 235.1 | 0.06 | 1.52 | 1 |  |
| *Pph* | 0.88 | 0.55 | 283.54 | -0.2 | 1.95 | 12 |  |
| *Ps*-9643 | 1.03 | 0.49 | 278.64 | 0.06 | 1.99 | 12 |  |
| *Pss*-9293 | 1.31 | 0.55 | 283.28 | 0.23 | 2.38 | 12 |  |
| R2-leaf | 1.98 | 0.52 | 281.13 | 0.96 | 2.99 | 12 |  |
| R1-5244 | 2.74 | 0.52 | 281.12 | 1.72 | 3.75 | 2 |  |
| *Pss*-9097 | 3.02 | 0.55 | 283.22 | 1.95 | 4.1 | 2 |  |

**Table S8: REML analysis of field inoculation of cherry inoculated by leaf scar.** The REML model and ANOVA are presented, followed by lsmean Tukey-HSD groupings for cultivars, strains and then strains on each cultivar (corresponds to groupings on Figure 3A-2).
