# Supplementary material for: Characterization of the pathogenicity of strains of Pseudomonas syringae towards cherry and plum
Source: Plant Pathol. 2018 Feb 14;67(5):1177–93. doi: 10.1111/ppa.12834 (PMC5993217; doi:10.1111/ppa.12834)
Supplement: Supplementary file 17 — Table S9. REML analysis of field inoculation of cherry inoculated by wound. [file PPA-67-1177-s017.docx]

| **l1 <- lmer(log2(length+1) ~ cv * strain + (1\|block/no.))** | | | | | |  |  |
| --- | --- | --- | --- | --- | --- | --- | --- |
|  |  |  |  |  |  |  |  |
| **Random effects** | |  |  |  |  |  |  |
| Groups | Name | Std.Dev. |  |  |  |  |  |
| no.:block | (Intercept) | 0.2 |  |  |  |  |  |
| block | (Intercept) | 0.13 |  |  |  |  |  |
| Residual | 0.76 |  |  |  |  |  |  |
|  |  |  |  |  |  |  |  |
| **ANOVA** |  |  |  |  |  |  |  |
|  | Sum sq | Mean Sq | NumDF | DenDF | F.value | Pr(>F) |  |
| cv | 10.72 | 3.57 | 3 | 58.35 | 6.21 | 0.001 | *** |
| strain | 107.01 | 13.38 | 8 | 268.37 | 23.25 | <2.20E-16 | *** |
| cv:strain | 19.49 | 0.81 | 24 | 266.85 | 1.41 | 0.1 |  |
| **Lsmeans Cultivars** | |  |  |  |  |  |  |
| cv | lsmean | SE | df | lower.CL | upper.CL | .group |  |
| Mglory | 4.1 | 0.1 | 38.85 | 3.89 | 4.3 | 1 |  |
| Napoleon | 4.44 | 0.1 | 43.51 | 4.23 | 4.65 | 12 |  |
| Van | 4.55 | 0.11 | 45.38 | 4.34 | 4.77 | 2 |  |
| Roundel | 4.64 | 0.12 | 51.09 | 4.41 | 4.87 | 2 |  |
| **Lsmeans Strains** | |  |  |  |  |  |  |
| strain | lsmean | SE | df | lower.CL | upper.CL | .group |  |
| Control | 3.87 | 0.11 | 68.38 | 3.64 | 4.09 | 1 |  |
| *Pph* | 3.92 | 0.15 | 153.88 | 3.63 | 4.21 | 1 |  |
| *Ps*-9643 | 3.93 | 0.14 | 138.91 | 3.66 | 4.21 | 1 |  |
| RMA1 | 4.01 | 0.15 | 156.83 | 3.72 | 4.31 | 1 |  |
| R1-5300 | 4.06 | 0.14 | 142.7 | 3.78 | 4.34 | 12 |  |
| *Pss*-9293 | 4.63 | 0.14 | 138.86 | 4.36 | 4.91 | 23 |  |
| R2-leaf | 4.82 | 0.15 | 151.05 | 4.53 | 5.11 | 3 |  |
| *Pss*-9097 | 5.1 | 0.13 | 124.91 | 4.83 | 5.36 | 34 |  |
| R1-5244 | 5.56 | 0.14 | 141.16 | 5.28 | 5.84 | 4 |  |
| **Lsmeans Strain x Cultivar** | |  |  |  |  |  |  |
| **Merton Glory** | |  |  |  |  |  |  |
| strain | lsmean | SE | df | lower.CL | upper.CL | .group |  |
| *Ps*-9643 | 3.74 | 0.25 | 284.89 | 3.25 | 4.24 | 1 |  |
| Control | 3.75 | 0.19 | 231.18 | 3.38 | 4.11 | 1 |  |
| RMA1 | 3.78 | 0.26 | 287.69 | 3.26 | 4.3 | 1 |  |
| *Pph* | 3.96 | 0.25 | 284.88 | 3.47 | 4.46 | 1 |  |
| R1-5300 | 4 | 0.25 | 284.88 | 3.5 | 4.49 | 1 |  |
| *Pss*-9293 | 4.2 | 0.25 | 284.89 | 3.7 | 4.69 | 1 |  |
| R2-leaf | 4.34 | 0.26 | 287.74 | 3.82 | 4.86 | 1 |  |
| R1-5244 | 4.48 | 0.26 | 287.71 | 3.96 | 5 | 1 |  |
| *Pss*-9097 | 4.62 | 0.25 | 284.82 | 4.12 | 5.11 | 1 |  |
|  |  |  |  |  |  |  |  |
| **Napoleon** |  |  |  |  |  |  |  |
| strain | lsmean | SE | df | lower.CL | upper.CL | .group |  |
| *Pph* | 3.65 | 0.28 | 290.71 | 3.1 | 4.2 | 1 |  |
| Control | 3.89 | 0.2 | 246.7 | 3.49 | 4.28 | 1 |  |
| RMA1 | 3.99 | 0.28 | 290.47 | 3.43 | 4.54 | 12 |  |
| *Ps*-9643 | 4.02 | 0.25 | 284.9 | 3.53 | 4.52 | 12 |  |
| R1-5300 | 4.07 | 0.25 | 284.91 | 3.58 | 4.57 | 12 |  |
| *Pss*-9293 | 4.58 | 0.25 | 284.9 | 4.09 | 5.08 | 12 |  |
| *Pss*-9097 | 4.94 | 0.25 | 284.9 | 4.44 | 5.43 | 23 |  |
| R2-leaf | 5.03 | 0.3 | 292.59 | 4.44 | 5.62 | 23 |  |
| R1-5244 | 5.83 | 0.26 | 287.95 | 5.31 | 6.35 | 3 |  |
|  |  |  |  |  |  |  |  |
| **Roundel** |  |  |  |  |  |  |  |
| .group | lsmean | SE | df | lower.CL | upper.CL | .group |  |
| *Pph* | 3.92 | 0.32 | 294.46 | 3.28 | 4.55 | 1 |  |
| *Ps*-9643 | 3.96 | 0.3 | 292.56 | 3.38 | 4.55 | 1 |  |
| Control | 3.99 | 0.23 | 270.95 | 3.53 | 4.44 | 1 |  |
| R1-5300 | 4.32 | 0.32 | 294.37 | 3.68 | 4.95 | 12 |  |
| RMA1 | 4.33 | 0.3 | 292.89 | 3.75 | 4.92 | 12 |  |
| *Pss*-9293 | 4.75 | 0.3 | 292.49 | 4.16 | 5.34 | 123 |  |
| *Pss*-9097 | 5.21 | 0.26 | 287.95 | 4.69 | 5.73 | 23 |  |
| R2-leaf | 5.47 | 0.28 | 290.41 | 4.92 | 6.02 | 23 |  |
| R1-5244 | 5.83 | 0.3 | 292.54 | 5.24 | 6.42 | 3 |  |
|  |  |  |  |  |  |  |  |
| **Van** |  |  |  |  |  |  |  |
| strain | lsmean | SE | df | lower.CL | upper.CL | .group |  |
| Control | 3.84 | 0.21 | 259.49 | 3.42 | 4.26 | 1 |  |
| R1-5300 | 3.85 | 0.26 | 287.83 | 3.33 | 4.37 | 12 |  |
| RMA1 | 3.96 | 0.3 | 292.64 | 3.37 | 4.54 | 12 |  |
| *Ps*-9643 | 4.01 | 0.28 | 290.54 | 3.46 | 4.56 | 12 |  |
| *Pph* | 4.14 | 0.28 | 290.37 | 3.59 | 4.69 | 12 |  |
| R2-leaf | 4.44 | 0.28 | 290.61 | 3.89 | 5 | 12 |  |
| *Pss*-9293 | 5 | 0.28 | 290.44 | 4.45 | 5.55 | 23 |  |
| *Pss*-9097 | 5.62 | 0.26 | 287.59 | 5.1 | 6.14 | 3 |  |
| R1-5244 | 6.11 | 0.26 | 287.5 | 5.59 | 6.64 | 3 |  |

**Table S9: REML analysis of field inoculation of cherry inoculated by wound.** The REML model and ANOVA are presented, followed by lsmean Tukey-HSD groupings for cultivars, strains and then strains on each cultivar (corresponds to groupings on Figure 3B-2).
