# Supplementary material for: Characterization of the pathogenicity of strains of Pseudomonas syringae towards cherry and plum
Source: Plant Pathol. 2018 Feb 14;67(5):1177–93. doi: 10.1111/ppa.12834 (PMC5993217; doi:10.1111/ppa.12834)
Supplement: Supplementary file 18 — Table S10. Proportional odds model (POM) analysis of the plum field inoculations. [file PPA-67-1177-s018.docx]

| **Optimising models** | | | | | | | | | | | | | | | | | | | | | | | | | | |
| --- | --- | --- | --- | --- | --- | --- | --- | --- | --- | --- | --- | --- | --- | --- | --- | --- | --- | --- | --- | --- | --- | --- | --- | --- | --- | --- |
|  | **formula:** | | | | | | | | | | | **link:** | | | | | | **threshold:** | | | | | | | | |
| fm1 | score ~ 1 | | | | | | | | | | | logit | | | | | | flexible | | | | | | | | |
| fm2 | score ~ strain + block | | | | | | | | | | | logit | | | | | | flexible | | | | | | | | |
| fm3 | score ~ strain + cv + block | | | | | | | | | | | logit | | | | | | flexible | | | | | | | | |
| fm4 | score ~ strain + cv + ino + block | | | | | | | | | | | logit | | | | | | flexible | | | | | | | | |
| fm7 | score ~ strain + cv * ino + block | | | | | | | | | | | logit | | | | | | flexible | | | | | | | | |
| fm6 | score ~ strain * ino + cv + block | | | | | | | | | | | logit | | | | | | flexible | | | | | | | | |
| fm5 | score ~ strain * cv + ino + block | | | | | | | | | | | logit | | | | | | flexible | | | | | | | | |
|  | **no.par** | | | | **AIC** | | **logLik** | | **LR.stat** | | **df** | | **Pr(>Chisq)** | | | | | | |  | | | | |  |  |
| fm1 | 3 | | | | 600.44 | | -297.22 | |  | |  | |  | | | | | | |  | | | | |  |  |
| fm2 | 20 | | | | 566.6 | | -263.3 | | 67.85 | | 17 | | 5.05E-08 | | | | | | | *** | | | | |  |  |
| fm3 | 21 | | | | 562.3 | | -260.15 | | 6.3 | | 1 | | 0.01 | | | | | | | * | | | | |  |  |
| fm4 | 22 | | | | 520.14 | | -238.07 | | 44.16 | | 1 | | 3.03E-11 | | | | | | | *** | | | | |  |  |
| fm7 | 23 | | | | 522 | | -238 | | 0.15 | | 1 | | 0.7 | | | | | | |  | | | | |  |  |
| fm5 | 30 | | | | 529.75 | | -234.88 | | 6.25 | | 7 | | 0.51 | | | | | | |  | | | | |  |  |
| fm6 | 30 | | | | 529.76 | | -234.88 | | -0.01 | | 0 | |  | | | | | | |  | | | | |  |  |
| **Final model: clm(score~strain+cv+ino + block,data=plum)** | | | | | | | | | | | | | | | | | | | | | | | | | |  |
| link | | threshold | | | | nobs | | logLik | | AIC | | niter | | | | max.grad | | | | | cond.H | | | | |  |
| logit | | flexible | | | | 295 | | -238.07 | | 520.14 | | 6(0) | | | | 2.50E-14 | | | | | 2.50E+02 | | | | |  |
| **Coefficients:** | | | | | | | |  | |  | | | |  | | |  | | | | |  | | | |  |
|  | | | Estimate | | | Std. | | Error | | z value | | | Pr(>\|z\|) | | | | | |  | | | | |  | |  |
| strain*Pph* | | | 0.69 | | | 0.57 | | 1.22 | | 0.22 | | | | |  | |  | | | | | |  | | |  |
| strain*Ps*-9643 | | | 0.8 | | | 0.56 | | 1.44 | | 0.15 | | | | |  | |  | | | | | |  | | |  |
| strain*Pss*-9097 | | | 2.89 | | | 0.52 | | 5.51 | | 3.53E-08 | | | | | *** | |  | | | | | |  | | |  |
| strain*Pss*-9293 | | | 2.24 | | | 0.52 | | 4.26 | | 2.01E-05 | | | | | *** | |  | | | | | |  | | |  |
| strainR1-5244 | | | 2.34 | | | 0.52 | | 4.5 | | 6.72E-06 | | | | | *** | |  | | | | | |  | | |  |
| strainR1-5300 | | | 2.58 | | | 0.52 | | 4.94 | | 7.79E-07 | | | | | *** | |  | | | | | |  | | |  |
| strainR2-leaf | | | 1.52 | | | 0.56 | | 2.72 | | 0.006 | | | | | ** | |  | | | | | |  | | |  |
| strainRMA1 | | | 0.84 | | | 0.59 | | 1.42 | | 0.16 | | | | |  | |  | | | | | |  | | |  |
| cvvictoria | | | 0.99 | | | 0.28 | | 3.54 | | 0.0004 | | | | | *** | |  | | | | | |  | | |  |
| inowound | | | 1.83 | | | 0.29 | | 6.24 | | 4.51E-10 | | | | | *** | |  | | | | | |  | | |  |
| blockB | | | 0.16 | | | 0.58 | | 0.27 | | 0.79 | | | | |  | |  | | | | | |  | | |  |
| blockC | | | -0.25 | | | 0.57 | | -0.44 | | 0.66 | | | | |  | |  | | | | | |  | | |  |
| blockD | | | -0.42 | | | 0.6 | | -0.71 | | 0.48 | | | | |  | |  | | | | | |  | | |  |
| blockE | | | -2.42 | | | 0.8 | | -3.01 | | 0.003 | | | | | ** | |  | | | | | |  | | |  |
| blockF | | | 0.82 | | | 0.56 | | 1.45 | | 0.15 | | | | |  | |  | | | | | |  | | |  |
| blockG | | | 0.33 | | | 0.55 | | 0.6 | | 0.55 | | | | |  | |  | | | | | |  | | |  |
| blockH | | | 0.2 | | | 0.54 | | 0.38 | | 0.71 | | | | |  | |  | | | | | |  | | |  |
| blockI | | | 0.63 | | | 0.53 | | 1.19 | | 0.23 | | | | |  | |  | | | | | |  | | |  |
| blockJ | | | | 0.05 | | 0.58 | | 0.09 | | 0.93 | | | |  | | |  | | | | |  | | | |  |

**Table S10: POM analysis of the plum field inoculations.** Model comparisons are first shown with the ANOVA comparing models. The summary of the final model (score~strain+cv+ino+block) is then presented.
