# Supplementary material for: Characterization of the pathogenicity of strains of Pseudomonas syringae towards cherry and plum
Source: Plant Pathol. 2018 Feb 14;67(5):1177–93. doi: 10.1111/ppa.12834 (PMC5993217; doi:10.1111/ppa.12834)
Supplement: Supplementary file 19 — Table S11. Lsmeans Tukey‐HSD groupings for different treatment combinations from the proportional odds model (POM) analysis of plum field inoculations. [file PPA-67-1177-s019.docx]

| **Lsmeans (strains on different cultivars)** | | | | | | |
| --- | --- | --- | --- | --- | --- | --- |
| **Marjorie's Seedling scar inoculation** | | | | | | |
| strain | lsmean | SE | df | asymp.LCL | asymp.UCL | .group |
| Control | -5.31 | 0.55 | NA | -6.39 | -4.23 | 1 |
| *Pph* | -4.62 | 0.58 | NA | -5.76 | -3.48 | 12 |
| *Ps*-9643 | -4.51 | 0.55 | NA | -5.59 | -3.43 | 12 |
| RMA1 | -4.47 | 0.6 | NA | -5.65 | -3.29 | 123 |
| R2-leaf | -3.79 | 0.52 | NA | -4.81 | -2.76 | 1234 |
| *Pss*-9293 | -3.07 | 0.47 | NA | -4 | -2.15 | 234 |
| R1-5244 | -2.96 | 0.47 | NA | -3.88 | -2.05 | 234 |
| R1-5300 | -2.73 | 0.47 | NA | -3.65 | -1.81 | 34 |
| *Pss*-9097 | -2.42 | 0.46 | NA | -3.32 | -1.52 | 4 |
| **Marjorie's Seedling wound inoculation** | | | | | | |
| strain | lsmean | SE | df | asymp.LCL | asymp.UCL | .group |
| Control | -3.48 | 0.44 | NA | -4.35 | -2.61 | 1 |
| *Pph* | -2.79 | 0.49 | NA | -3.76 | -1.83 | 12 |
| *Ps*-9643 | -2.68 | 0.48 | NA | -3.62 | -1.75 | 12 |
| RMA1 | -2.64 | 0.52 | NA | -3.66 | -1.63 | 123 |
| R2-leaf | -1.96 | 0.46 | NA | -2.86 | -1.05 | 1234 |
| *Pss*-9293 | -1.25 | 0.41 | NA | -2.05 | -0.44 | 234 |
| R1-5244 | -1.14 | 0.4 | NA | -1.93 | -0.34 | 234 |
| R1-5300 | -0.91 | 0.41 | NA | -1.7 | -0.11 | 34 |
| *Pss*-9097 | -0.59 | 0.41 | NA | -1.39 | 0.2 | 4 |
| **Victoria scar inoculation** | | | | | | |
| strain | lsmean | SE | df | asymp.LCL | asymp.UCL | .group |
| Control | -4.32 | 0.49 | NA | -5.27 | -3.37 | 1 |
| *Pph* | -3.63 | 0.53 | NA | -4.66 | -2.59 | 12 |
| *Ps*-9643 | -3.52 | 0.5 | NA | -4.51 | -2.53 | 12 |
| RMA1 | -3.48 | 0.55 | NA | -4.56 | -2.39 | 123 |
| R2-leaf | -2.79 | 0.48 | NA | -3.74 | -1.84 | 1234 |
| *Pss*-9293 | -2.08 | 0.42 | NA | -2.91 | -1.25 | 234 |
| R1-5244 | -1.97 | 0.41 | NA | -2.78 | -1.17 | 234 |
| R1-5300 | -1.74 | 0.41 | NA | -2.55 | -0.93 | 34 |
| *Pss*-9097 | -1.43 | 0.4 | NA | -2.22 | -0.64 | 4 |
| **Victoria wound inoculation** | | | | | | |
| strain | lsmean | SE | df | asymp.LCL | asymp.UCL | .group |
| Control | -2.49 | 0.41 | NA | -3.28 | -1.7 | 1 |
| *Pph* | -1.8 | 0.47 | NA | -2.72 | -0.88 | 12 |
| *Ps*-9643 | -1.69 | 0.46 | NA | -2.59 | -0.79 | 12 |
| RMA1 | -1.65 | 0.5 | NA | -2.63 | -0.67 | 123 |
| R2-leaf | -0.97 | 0.46 | NA | -1.87 | -0.07 | 1234 |
| *Pss*-9293 | -0.25 | 0.4 | NA | -1.04 | 0.53 | 234 |
| R1-5244 | -0.14 | 0.39 | NA | -0.91 | 0.62 | 234 |
| R1-5300 | 0.09 | 0.39 | NA | -0.67 | 0.85 | 34 |
| *Pss*-9097 | 0.4 | 0.39 | NA | -0.36 | 1.16 | 4 |
| **Lsmeans (cultivars with different inoculation methods)** | | | | | | |
| **Marjorie's Seedling** | | | | | | |
| ino | lsmean | SE | df | asymp.LCL | asymp.UCL | .group |
| scar | -3.77 | 0.36 | NA | -4.48 | -3.05 | 1 |
| wound | -1.94 | 0.25 | NA | -2.43 | -1.45 | 2 |
| **Victoria** | | | | | | |
| ino | lsmean | SE | df | asymp.LCL | asymp.UCL | .group |
| scar | -2.77 | 0.29 | NA | -3.33 | -2.21 | 1 |
| wound | -0.95 | 0.22 | NA | -1.37 | -0.52 | 2 |
| **Lsmeans (strains with different inoculation methods)** | | | | |  |  |
| **Scar inoculation** | |  |  |  |  |  |
| strain | lsmean | SE | df | asymp.LCL | asymp.UCL | .group |
| Control | -4.81 | 0.5 | NA | -5.79 | -3.83 | 1 |
| *Pph* | -4.12 | 0.54 | NA | -5.18 | -3.07 | 12 |
| *Ps*-9643 | -4.02 | 0.51 | NA | -5.01 | -3.02 | 12 |
| RMA1 | -3.97 | 0.56 | NA | -5.07 | -2.88 | 123 |
| R2-leaf | -3.29 | 0.48 | NA | -4.24 | -2.34 | 1234 |
| *Pss*-9293 | -2.58 | 0.43 | NA | -3.41 | -1.74 | 234 |
| R1-5244 | -2.47 | 0.42 | NA | -3.28 | -1.65 | 234 |
| R1-5300 | -2.24 | 0.42 | NA | -3.06 | -1.42 | 34 |
| *Pss*-9097 | -1.92 | 0.41 | NA | -2.73 | -1.12 | 4 |
| **Wound inoculation** | |  |  |  |  |  |
| strain | lsmean | SE | df | asymp.LCL | asymp.UCL | .group |
| Control | -2.99 | 0.4 | NA | -3.77 | -2.2 | 1 |
| *Pph* | -2.3 | 0.46 | NA | -3.2 | -1.39 | 12 |
| *Ps*-9643 | -2.19 | 0.45 | NA | -3.06 | -1.31 | 12 |
| RMA1 | -2.15 | 0.49 | NA | -3.11 | -1.19 | 123 |
| R2-leaf | -1.46 | 0.44 | NA | -2.32 | -0.6 | 1234 |
| *Pss*-9293 | -0.75 | 0.38 | NA | -1.5 | 0 | 234 |
| R1-5244 | -0.64 | 0.37 | NA | -1.37 | 0.09 | 234 |
| R1-5300 | -0.41 | 0.37 | NA | -1.14 | 0.32 | 34 |
| *Pss*-9097 | -0.1 | 0.37 | NA | -0.82 | 0.63 | 4 |

**Table S11: Lsmeans Tukey-HSD groupings for different treatment combinations from the POM analysis of plum field inoculations.** Groups for strains on different cultivars with each inoculation method are presented, followed by groupings of cultivars using each inoculation method and then strains across the two inoculation methods. This table corresponds to the statistical groupings seen in Figure 4A1/B1.
