# Supplementary material for: Characterization of the pathogenicity of strains of Pseudomonas syringae towards cherry and plum
Source: Plant Pathol. 2018 Feb 14;67(5):1177–93. doi: 10.1111/ppa.12834 (PMC5993217; doi:10.1111/ppa.12834)
Supplement: Supplementary file 20 — Table S12. REML analysis of field inoculation of plum inoculated by leaf scar. [file PPA-67-1177-s020.docx]

| l1 <- lmer(log2(length+1) ~ cv * strain + (1\|block/no.)) | | | | | |  |  |
| --- | --- | --- | --- | --- | --- | --- | --- |
|  |  |  |  |  |  |  |  |
| REML criterion at convergence: 430.77 | | | |  |  |  |  |
| Random | effects: |  |  |  |  |  |  |
| Groups | Name | Std.Dev. |  |  |  |  |  |
| no.:block | (Intercept) | 0.23 |  |  |  |  |  |
| block | (Intercept) | 0.43 |  |  |  |  |  |
| Residual | 1.23 |  |  |  |  |  |  |
|  |  |  |  |  |  |  |  |
| **ANOVA** |  |  |  |  |  |  |  |
|  | Sum Sq | Mean Sq | NumDF | DenDF | F.value | Pr(>F) |  |
| cv | 2.65 | 2.65 | 1 | 19.8 | 1.75 | 0.2 |  |
| strain | 38.06 | 4.76 | 8 | 105.17 | 3.15 | 0.003 | ** |
| cv:strain | 15.82 | 1.98 | 8 | 105.14 | 1.31 | 0.25 |  |
| **Lsmeans Strains** | |  |  |  |  |  |  |
| strain | lsmean | SE | df | lower.CL | upper.CL | .group |  |
| Control | 0.36 | 0.29 | 39.51 | -0.22 | 0.94 | 1 |  |
| *Pph* | 0.53 | 0.38 | 83.59 | -0.23 | 1.3 | 1 |  |
| *Ps*-9643 | 0.65 | 0.36 | 70.92 | -0.07 | 1.37 | 1 |  |
| R2-leaf | 0.73 | 0.37 | 77.04 | 0 | 1.46 | 12 |  |
| *Pss*-9293 | 1.13 | 0.37 | 74.49 | 0.4 | 1.87 | 12 |  |
| RMA1 | 1.14 | 0.39 | 86.39 | 0.36 | 1.92 | 12 |  |
| R1-5244 | 1.21 | 0.36 | 73.43 | 0.5 | 1.93 | 12 |  |
| R1-5300 | 1.57 | 0.38 | 77.48 | 0.82 | 2.33 | 12 |  |
| *Pss*-9097 | 2.2 | 0.39 | 80.85 | 1.43 | 2.96 | 2 |  |
| **Lsmeans Strain x Cultivar** | |  |  |  |  |  |  |
| **Marjorie's Seedling** | |  |  |  |  |  |  |
| strain | lsmean | SE | df | lower.CL | upper.CL | .group |  |
| Control | 0.14 | 0.41 | 84.93 | -0.67 | 0.96 | 1 |  |
| *Pph* | 0.21 | 0.58 | 114.33 | -0.95 | 1.36 | 1 |  |
| *Pss*-9293 | 0.61 | 0.53 | 111.24 | -0.45 | 1.67 | 1 |  |
| *Ps*-9643 | 0.77 | 0.53 | 111.3 | -0.29 | 1.83 | 1 |  |
| R2-leaf | 0.96 | 0.53 | 111.24 | -0.1 | 2.02 | 1 |  |
| R1-5244 | 1.22 | 0.53 | 111.24 | 0.16 | 2.27 | 1 |  |
| *Pss*-9097 | 1.27 | 0.58 | 114.32 | 0.11 | 2.42 | 1 |  |
| R1-5300 | 1.33 | 0.58 | 114.38 | 0.17 | 2.48 | 1 |  |
| RMA1 | 1.51 | 0.58 | 114.39 | 0.35 | 2.66 | 1 |  |
|  |  |  |  |  |  |  |  |
| **Victoria** |  |  |  |  |  |  |  |
| strain | lsmean | SE | df | lower.CL | upper.CL | .group |  |
| R2-leaf | 0.5 | 0.46 | 105.68 | -0.42 | 1.42 | 1 |  |
| *Ps*-9643 | 0.54 | 0.44 | 100.8 | -0.34 | 1.41 | 1 |  |
| Control | 0.57 | 0.35 | 71.31 | -0.13 | 1.28 | 1 |  |
| RMA1 | 0.77 | 0.5 | 109.3 | -0.21 | 1.75 | 1 |  |
| *Pph* | 0.86 | 0.46 | 105.68 | -0.07 | 1.78 | 1 |  |
| R1-5244 | 1.21 | 0.44 | 101.78 | 0.34 | 2.08 | 12 |  |
| *Pss*-9293 | 1.66 | 0.47 | 105.09 | 0.73 | 2.58 | 12 |  |
| R1-5300 | 1.82 | 0.44 | 100.73 | 0.94 | 2.69 | 12 |  |
| *Pss*-9097 | 3.13 | 0.47 | 105.13 | 2.2 | 4.05 | 2 |  |

**Table S12: REML analysis of field inoculation of plum inoculated by leaf scar.** The REML model and ANOVA are presented, followed by lsmean Tukey-HSD groupings for strains and then strains on each cultivar (corresponds to groupings on Figure 4A2).
