# Supplementary material for: Characterization of the pathogenicity of strains of Pseudomonas syringae towards cherry and plum
Source: Plant Pathol. 2018 Feb 14;67(5):1177–93. doi: 10.1111/ppa.12834 (PMC5993217; doi:10.1111/ppa.12834)
Supplement: Supplementary file 21 — Table S13. REML analysis of field inoculation of plum inoculated by wound. [file PPA-67-1177-s021.docx]

| **l1 <- lmer(log2(length+1) ~ cv * strain + (1\|block/no.), data = plumwound)** | | | | | | | |
| --- | --- | --- | --- | --- | --- | --- | --- |
|  |  |  |  |  |  |  |  |
| REML criterion at convergence: 336.81 | | | |  |  |  |  |
| Random effects: | | | | | | | |
| Groups | Name | Std.Dev. |  |  |  |  |  |
| no.:block | (Intercept) | 0 |  |  |  |  |  |
| block | (Intercept) | 0.27 |  |  |  |  |  |
| Residual | 0.67 |  |  |  |  |  |  |
|  |  |  |  |  |  |  |  |
| **ANOVA** |  |  |  |  |  |  |  |
|  | Sum Sq | Mean Sq | NumDF | DenDF | F.value | Pr(>F) |  |
| cv | 0.39 | 0.39 | 1 | 137.49 | 0.87 | 0.35 |  |
| strain | 18.59 | 2.32 | 8 | 132.48 | 5.18 | 1.21E-05 | *** |
| cv:strain | 2.56 | 0.32 | 8 | 132.51 | 0.71 | 0.68 |  |
| **Lsmeans Strains** | | |  |  |  |  |  |
| strain | lsmean | SE | df | lower.CL | upper.CL | .group |  |
| Control | 3.6 | 0.15 | 37.51 | 3.3 | 3.9 | 1 |  |
| RMA1 | 3.71 | 0.2 | 85.79 | 3.31 | 4.1 | 1 |  |
| *Ps*-9643 | 3.71 | 0.18 | 68.87 | 3.35 | 4.07 | 1 |  |
| *Pph* | 3.97 | 0.19 | 76.19 | 3.59 | 4.34 | 12 |  |
| *Pss*-9293 | 4.06 | 0.19 | 76.19 | 3.68 | 4.43 | 12 |  |
| R2-leaf | 4.11 | 0.2 | 85.77 | 3.72 | 4.51 | 12 |  |
| R1-5244 | 4.15 | 0.19 | 76.18 | 3.77 | 4.52 | 12 |  |
| R1-5300 | 4.48 | 0.18 | 72.16 | 4.11 | 4.84 | 2 |  |
| *Pss*-9097 | 4.63 | 0.19 | 74.05 | 4.26 | 5 | 2 |  |
| **Lsmeans Strain x cultivar** | | |  |  |  |  |  |
| **Marjorie's Seedling** | |  |  |  |  |  |  |
| strain | lsmean | SE | df | lower.CL | upper.CL | .group |  |
| *Ps*-9643 | 3.5 | 0.23 | 109.5 | 3.05 | 3.95 | 1 |  |
| RMA1 | 3.61 | 0.27 | 126.73 | 3.08 | 4.15 | 12 |  |
| Control | 3.62 | 0.19 | 80.75 | 3.24 | 4 | 1 |  |
| *Pss*-9293 | 3.84 | 0.25 | 121.26 | 3.35 | 4.34 | 12 |  |
| R2-leaf | 3.93 | 0.27 | 126.82 | 3.4 | 4.46 | 12 |  |
| *Pph* | 4.09 | 0.25 | 121.24 | 3.59 | 4.59 | 12 |  |
| R1-5244 | 4.32 | 0.25 | 121.23 | 3.82 | 4.82 | 12 |  |
| R1-5300 | 4.42 | 0.25 | 121.23 | 3.92 | 4.92 | 12 |  |
| *Pss*-9097 | 4.59 | 0.27 | 126.73 | 4.06 | 5.12 | 2 |  |
|  |  |  |  |  |  |  |  |
| **Victoria** |  |  |  |  |  |  |  |
| strain | lsmean | SE | df | lower.CL | upper.CL | .group |  |
| Control | 3.58 | 0.19 | 76.15 | 3.2 | 3.95 | 1 |  |
| RMA1 | 3.8 | 0.27 | 126.77 | 3.27 | 4.33 | 12 |  |
| *Pph* | 3.84 | 0.25 | 121.32 | 3.34 | 4.34 | 12 |  |
| *Ps*-9643 | 3.91 | 0.25 | 121.36 | 3.41 | 4.41 | 12 |  |
| R1-5244 | 3.98 | 0.25 | 121.23 | 3.48 | 4.47 | 12 |  |
| *Pss*-9293 | 4.27 | 0.25 | 121.24 | 3.77 | 4.77 | 12 |  |
| R2-leaf | 4.29 | 0.27 | 126.71 | 3.76 | 4.83 | 12 |  |
| R1-5300 | 4.53 | 0.24 | 115.44 | 4.06 | 5.01 | 2 |  |
| *Pss*-9097 | 4.67 | 0.23 | 109.5 | 4.22 | 5.13 | 2 |  |

**Table S13: REML analysis of field inoculation of plum inoculated by wound.** The REML model and ANOVA are presented, followed by lsmean Tukey-HSD groupings for strains and then strains on each cultivar (corresponds to groupings on Figure 4B2).
