# Supplementary material for: Characterization of the pathogenicity of strains of Pseudomonas syringae towards cherry and plum
Source: Plant Pathol. 2018 Feb 14;67(5):1177–93. doi: 10.1111/ppa.12834 (PMC5993217; doi:10.1111/ppa.12834)
Supplement: Supplementary file 22 — Table S14. ANOVA table of cut shoot inoculations. [file PPA-67-1177-s022.docx]

| **ANOVA** |  | |  | | |  |  | | |  | |  | | | | |
| --- | --- | --- | --- | --- | --- | --- | --- | --- | --- | --- | --- | --- | --- | --- | --- | --- |
|  | Df | | Sum Sq | | | Mean Sq | F value | | | Pr(>F) | |  | | | | |
| strain | 8 | | 403.8 | | | 50.47 | 36.81 | | | <2.00E-16 | | *** | | | | |
| host | 1 | | 0.4 | | | 0.45 | 0.33 | | | 0.57 | |  | | | | |
| block | 9 | | 20.1 | | | 2.24 | 1.63 | | | 0.1 | |  | | | | |
| strain:host | 8 | | 138.7 | | | 17.34 | 12.64 | | | <2.00E-16 | | *** | | | | |
| strain:host:cv | 36 | | 148 | | | 4.11 | 3 | | | 4.92E-08 | | *** | | | | |
| Residuals | 474 | | 649.9 | | | 1.37 |  | | |  | |  | | | | |
| **Groups** |  | |  | | |  |  | |  | | | | |  | |  |
| **Merton Glory** | | | |  |  | | | | |  |  | |  | |  |  |
| strain | | lsmean | | SE | df | | | lower.CL | | | upper.CL | | .group | |  |  |
| Control | | 1.06 | | 0.37 | 474 | | | 0.33 | | | 1.79 | | 1 | |  |  |
| *Psv* | | 1.08 | | 0.37 | 474 | | | 0.35 | | | 1.81 | | 1 | |  |  |
| R1-5300 | | 1.19 | | 0.37 | 474 | | | 0.46 | | | 1.92 | | 1 | |  |  |
| *Pph* | | 1.25 | | 0.37 | 474 | | | 0.52 | | | 1.98 | | 1 | |  |  |
| *Pss*-9293 | | 1.68 | | 0.37 | 474 | | | 0.95 | | | 2.41 | | 1 | |  |  |
| RMA1 | | 1.91 | | 0.39 | 474 | | | 1.14 | | | 2.67 | | 1 | |  |  |
| R1-5244 | | 2.56 | | 0.37 | 474 | | | 1.83 | | | 3.29 | | 12 | |  |  |
| *Pss*-9097 | | 3.61 | | 0.37 | 474 | | | 2.88 | | | 4.34 | | 2 | |  |  |
| R2-5255 | | 3.82 | | 0.37 | 474 | | | 3.1 | | | 4.55 | | 2 | |  |  |
|  | |  | |  |  | | |  | | |  | |  | |  |  |
| **Napoleon** | |  | |  |  | | |  | | |  | |  | |  |  |
| strain | | lsmean | | SE | df | | | lower.CL | | | upper.CL | | .group | |  |  |
| Control | | 1.12 | | 0.37 | 474 | | | 0.4 | | | 1.85 | | 1 | |  |  |
| *Pph* | | 1.61 | | 0.37 | 474 | | | 0.89 | | | 2.34 | | 1 | |  |  |
| *Psv* | | 1.69 | | 0.37 | 474 | | | 0.96 | | | 2.42 | | 1 | |  |  |
| R1-5300 | | 1.81 | | 0.37 | 474 | | | 1.08 | | | 2.54 | | 1 | |  |  |
| *Pss*-9293 | | 1.96 | | 0.37 | 474 | | | 1.23 | | | 2.68 | | 1 | |  |  |
| RMA1 | | 1.98 | | 0.37 | 474 | | | 1.25 | | | 2.71 | | 1 | |  |  |
| R1-5244 | | 3.62 | | 0.37 | 474 | | | 2.89 | | | 4.35 | | 2 | |  |  |
| R2-5255 | | 4.04 | | 0.37 | 474 | | | 3.31 | | | 4.77 | | 2 | |  |  |
| *Pss*-9097 | | 4.95 | | 0.37 | 474 | | | 4.22 | | | 5.68 | | 2 | |  |  |
|  | |  | |  |  | | |  | | |  | |  | |  |  |
| **Roundel** | |  | |  |  | | |  | | |  | |  | |  |  |
| strain | | lsmean | | SE | df | | | lower.CL | | | upper.CL | | .group | |  |  |
| *Pph* | | 1.01 | | 0.37 | 474 | | | 0.28 | | | 1.74 | | 1 | |  |  |
| *Psv* | | 1.36 | | 0.37 | 474 | | | 0.63 | | | 2.09 | | 1 | |  |  |
| R1-5300 | | 1.43 | | 0.39 | 474 | | | 0.66 | | | 2.2 | | 1 | |  |  |
| *Pss*-9293 | | 1.51 | | 0.37 | 474 | | | 0.79 | | | 2.24 | | 1 | |  |  |
| Control | | 1.53 | | 0.37 | 474 | | | 0.8 | | | 2.26 | | 1 | |  |  |
| RMA1 | | 1.91 | | 0.39 | 474 | | | 1.15 | | | 2.68 | | 12 | |  |  |
| R1-5244 | | 3.27 | | 0.37 | 474 | | | 2.54 | | | 3.99 | | 23 | |  |  |
| *Pss*-9097 | | 4.29 | | 0.37 | 474 | | | 3.56 | | | 5.02 | | 34 | |  |  |
| R2-5255 | | 5.15 | | 0.37 | 474 | | | 4.42 | | | 5.88 | | 4 | |  |  |
|  | |  | |  |  | | |  | | |  | |  | |  |  |
| **Van** | |  | |  |  | | |  | | |  | |  | |  |  |
| strain | | lsmean | | SE | df | | | lower.CL | | | upper.CL | | .group | |  |  |
| Control | | 1.19 | | 0.37 | 474 | | | 0.46 | | | 1.92 | | 1 | |  |  |
| *Pph* | | 1.35 | | 0.37 | 474 | | | 0.62 | | | 2.07 | | 1 | |  |  |
| *Psv* | | 1.45 | | 0.37 | 474 | | | 0.72 | | | 2.18 | | 1 | |  |  |
| R1-5300 | | 1.99 | | 0.37 | 474 | | | 1.26 | | | 2.71 | | 12 | |  |  |
| *Pss*-9293 | | 2.41 | | 0.37 | 474 | | | 1.68 | | | 3.14 | | 12 | |  |  |
| RMA1 | | 2.62 | | 0.37 | 474 | | | 1.89 | | | 3.35 | | 123 | |  |  |
| R2-5255 | | 3.25 | | 0.37 | 474 | | | 2.52 | | | 3.98 | | 234 | |  |  |
| R1-5244 | | 4.18 | | 0.37 | 474 | | | 3.45 | | | 4.91 | | 34 | |  |  |
| *Pss*-9097 | | 4.48 | | 0.37 | 474 | | | 3.76 | | | 5.21 | | 4 | |  |  |
|  | |  | |  |  | | |  | | |  | |  | |  |  |
| **Marjorie's Seedling** | | | |  |  | | |  | | |  | |  | |  |  |
| strain | | lsmean | | SE | df | | | lower.CL | | | upper.CL | | .group | |  |  |
| R1-5244 | | 1.22 | | 0.37 | 474 | | | 0.49 | | | 1.94 | | 1 | |  |  |
| Control | | 1.47 | | 0.37 | 474 | | | 0.74 | | | 2.19 | | 1 | |  |  |
| *Pss*-9293 | | 1.6 | | 0.37 | 474 | | | 0.87 | | | 2.33 | | 1 | |  |  |
| R2-5255 | | 1.69 | | 0.37 | 474 | | | 0.96 | | | 2.41 | | 1 | |  |  |
| *Pph* | | 1.69 | | 0.37 | 474 | | | 0.96 | | | 2.42 | | 1 | |  |  |
| RMA1 | | 1.73 | | 0.37 | 474 | | | 1 | | | 2.46 | | 1 | |  |  |
| R1-5300 | | 2.02 | | 0.37 | 474 | | | 1.29 | | | 2.75 | | 1 | |  |  |
| *Psv* | | 2.17 | | 0.37 | 474 | | | 1.44 | | | 2.9 | | 1 | |  |  |
| *Pss*-9097 | | 2.39 | | 0.37 | 474 | | | 1.66 | | | 3.12 | | 1 | |  |  |
|  | |  | |  |  | | |  | | |  | |  | |  |  |
| **Victoria** | |  | |  |  | | |  | | |  | |  | |  |  |
| strain | | lsmean | | SE | df | | | lower.CL | | | upper.CL | | .group | |  |  |
| *Psv* | | 1.44 | | 0.37 | 474 | | | 0.72 | | | 2.17 | | 1 | |  |  |
| R1-5244 | | 1.94 | | 0.37 | 474 | | | 1.21 | | | 2.66 | | 12 | |  |  |
| Control | | 2.05 | | 0.37 | 474 | | | 1.32 | | | 2.78 | | 12 | |  |  |
| *Pph* | | 2.29 | | 0.37 | 474 | | | 1.57 | | | 3.02 | | 12 | |  |  |
| *Pss*-9293 | | 2.9 | | 0.37 | 474 | | | 2.17 | | | 3.63 | | 123 | |  |  |
| R2-5255 | | 2.95 | | 0.37 | 474 | | | 2.23 | | | 3.68 | | 123 | |  |  |
| R1-5300 | | 3.28 | | 0.37 | 474 | | | 2.56 | | | 4.01 | | 23 | |  |  |
| *Pss*-9097 | | 4.31 | | 0.37 | 474 | | | 3.58 | | | 5.04 | | 3 | |  |  |
| RMA1 | | 4.46 | | 0.37 | 474 | | | 3.73 | | | 5.19 | | 3 | |  |  |
| **Groups (comparing cultivars)** | | | | | | | | | | | | | | |  |  |
| cv | | lsmean | | SE | df | | | lower.CL | | | upper.CL | | .group | |  |  |
| Marjorie’s Seedling | | 1.77 | | 0.12 | 474 | | | 1.53 | | | 2.02 | | 1 | |  |  |
| Merton Glory | | 2.02 | | 0.12 | 474 | | | 1.77 | | | 2.26 | | 12 | |  |  |
| Roundel | | 2.38 | | 0.12 | 474 | | | 2.14 | | | 2.63 | | 23 | |  |  |
| Napoleon | | 2.53 | | 0.12 | 474 | | | 2.29 | | | 2.77 | | 3 | |  |  |
| Van | | 2.55 | | 0.12 | 474 | | | 2.3 | | | 2.79 | | 3 | |  |  |
| Victoria | | 2.85 | | 0.12 | 474 | | | 2.61 | | | 3.09 | | 3 | |  |  |

**Table S14: ANOVA table of cut shoot inoculations** followed by lsmeans Tukey-HSD groupings for the strains on each cultivar, and then just the comparison of cultivars (corresponds to groupings on Figure 5).
