# Supplementary material for: Characterization of the pathogenicity of strains of Pseudomonas syringae towards cherry and plum
Source: Plant Pathol. 2018 Feb 14;67(5):1177–93. doi: 10.1111/ppa.12834 (PMC5993217; doi:10.1111/ppa.12834)
Supplement: Supplementary file 23 — Table S15. ANOVA table of immature cherry fruit inoculations. [file PPA-67-1177-s023.docx]

| **ANOVA** |  |  |  |  |  |  |
| --- | --- | --- | --- | --- | --- | --- |
|  | Df | Sum Sq | Mean Sq | F.value | Pr(>F) |  |
| strain | 21 | 71.25 | 3.39 | 32.83 | <2.00E-16 | *** |
| rep | 4 | 1.98 | 0.5 | 4.79 | 0.002 | ** |
| Residuals | 84 | 8.68 | 0.1 |  |  |  |
|  |  |  |  |  |  |  |
| **Groups** |  |  |  |  |  |  |
| trt | means | M |  |  |  |  |
| *Pss*-9097 | 3.71 | a |  |  |  |  |
| *Pss*-9630 | 3.57 | ab |  |  |  |  |
| *Pss*-9644 | 3.17 | abc |  |  |  |  |
| *Pss*-9654 | 3.04 | abc |  |  |  |  |
| *Pss*-9293 | 3.02 | abc |  |  |  |  |
| *Pss*-9659 | 2.93 | bc |  |  |  |  |
| *Pss*-9656 | 2.65 | cd |  |  |  |  |
| R2-5255 | 1.9 | de |  |  |  |  |
| R2-SC214 | 1.85 | e |  |  |  |  |
| R1-5244 | 1.85 | e |  |  |  |  |
| *Ps*-9643 | 1.73 | ef |  |  |  |  |
| R1-9646 | 1.69 | ef |  |  |  |  |
| R2-leaf | 1.6 | ef |  |  |  |  |
| R2-5260 | 1.56 | ef |  |  |  |  |
| R1-5300 | 1.56 | ef |  |  |  |  |
| R1-9326 | 1.55 | ef |  |  |  |  |
| R1-9657 | 1.5 | ef |  |  |  |  |
| R1-9629 | 1.42 | ef |  |  |  |  |
| RMA1 | 1.31 | ef |  |  |  |  |
| *Psv* | 1.23 | ef |  |  |  |  |
| *Pph* | 1.2 | ef |  |  |  |  |
| Control | 1.07 | f |  |  |  |  |

**Table S15: ANOVA table of immature cherry fruit inoculations** of all strains followed by Tukey-HSD groupings of the strains (corresponds to groupings on Figure 6A).
