# Supplementary material for: Characterization of the pathogenicity of strains of Pseudomonas syringae towards cherry and plum
Source: Plant Pathol. 2018 Feb 14;67(5):1177–93. doi: 10.1111/ppa.12834 (PMC5993217; doi:10.1111/ppa.12834)
Supplement: Supplementary file 24 — Table S16. REML analysis of immature cherry fruit inoculations where bacterial strains were inoculated onto different host cultivars. [file PPA-67-1177-s024.docx]

| model: lmer(log2(length) ~ cultivar * strain + (1\|exp/block)) | | | | | | | | | | | | | | | | | | | | | | | | | | | | | | | | |  | | | | |  | |  |  |  |
| --- | --- | --- | --- | --- | --- | --- | --- | --- | --- | --- | --- | --- | --- | --- | --- | --- | --- | --- | --- | --- | --- | --- | --- | --- | --- | --- | --- | --- | --- | --- | --- | --- | --- | --- | --- | --- | --- | --- | --- | --- | --- | --- |
|  | | | | | |  | | | | | | |  | | | | | | |  |  | | | | | | |  | | | | |  | | | | |  | |  |  |  |
| REML criterion at convergence: 215.27 | | | | | | | | | | | | | | | | | | | | |  | | | | | | |  | | | | |  | | | | |  | |  |  |  |
| Random effects: | | | | | | | | | | | | |  | | | | | | |  |  | | | | | | |  | | | | |  | | | | |  | |  |  |  |
| Groups | | | | | | Name | | | | | | | Std.Dev. | | | | | | |  |  | | | | | | |  | | | | |  | | | | |  | |  |  |  |
| block:exp | | | | | | (Intercept) | | | | | | | 0.05 | | | | | | |  |  | | | | | | |  | | | | |  | | | | |  | |  |  |  |
| exp | | | | | | (Intercept) | | | | | | | 0.05 | | | | | | |  |  | | | | | | |  | | | | |  | | | | |  | |  |  |  |
| Residual | | | | | | 0.32651 | | | | | | |  | | | | | | |  |  | | | | | | |  | | | | |  | | | | |  | |  |  |  |
|  | | | | | |  | | | | | | |  | | | | | | |  |  | | | | | | |  | | | | |  | | | | |  | |  |  |  |
| **ANOVA** | | | | | |  | | | | | | |  | | | | | | |  |  | | | | | | |  | | | | |  | | | | | |  | |  |  |
|  | | | Sum Sq | | | | Mean Sq | | | | | | NumDF | | | | | | | DenDF | | | | | | | F.value | | | | | | Pr(>F) | | | | | |  | |  |  |
| cultivar | | | 2.62 | | | | 0.87 | | | | | | 3 | | | | | | | 256.61 | | | | | | | 8.19 | | | | | | 3.17E-05 | | | | | | *** | |  |  |
| strain | | | 177.93 | | | | 59.31 | | | | | | 3 | | | | | | | 256.05 | | | | | | | 556.33 | | | | | | <2.20E-16 | | | | | | *** | |  |  |
| cultivar:  strain | | | 2.89 | | | | 0.32 | | | | | | 9 | | | | | | | 256.11 | | | | | | | 3.01 | | | | | | 0.002 | | | | | | ** | |  |  |
| **Lsmeans Cultivars** | | | | | | | | | | | | |  | | | | | | |  | | | | |  | | | | | |  | | | | |  | | | |  |  |  |
| **Merton Glory** | | | | | | | | | | | | |  | | | | | | |  | | | | |  | | | | | |  | | | | |  | | | |  |  |  |
| strain | | | | | | lsmean | | | | | | SE | | | | df | | | | | | lower.CL | | | | | | | upper.CL | | | | | .group | | | | | |  |  |  |
| Control | | | | | | 1.01 | | | | | | 0.08 | | | | 13.77 | | | | | | 0.83 | | | | | | | 1.19 | | | | | 1 | | | | | |  |  |  |
| R2-5255 | | | | | | 1.6 | | | | | | 0.09 | | | | 14.97 | | | | | | 1.42 | | | | | | | 1.78 | | | | | 2 | | | | | |  |  |  |
| R1-5244 | | | | | | 1.62 | | | | | | 0.09 | | | | 16.35 | | | | | | 1.43 | | | | | | | 1.81 | | | | | 2 | | | | | |  |  |  |
| *Pss*-9097 | | | | | | 3.04 | | | | | | 0.08 | | | | 12.73 | | | | | | 2.86 | | | | | | | 3.22 | | | | | 3 | | | | | |  |  |  |
|  | | | | | |  | | | | | |  | | | |  | | | | | |  | | | | | | |  | | | | |  | | | | | |  |  |  |
| **Napoleon** | | | | | |  | | | | | |  | | | |  | | | | | |  | | | | | | |  | | | | |  | | | | | |  |  |  |
| strain | | | | | | lsmean | | | | | | SE | | | | df | | | | | | lower.CL | | | | | | | upper.CL | | | | | .group | | | | | |  |  |  |
| Control | | | | | | 0.92 | | | | | | 0.09 | | | | 14.97 | | | | | | 0.74 | | | | | | | 1.11 | | | | | 1 | | | | | |  |  |  |
| R2-5255 | | | | | | 1.74 | | | | | | 0.09 | | | | 14.97 | | | | | | 1.56 | | | | | | | 1.93 | | | | | 2 | | | | | |  |  |  |
| R1-5244 | | | | | | 1.75 | | | | | | 0.08 | | | | 13.77 | | | | | | 1.56 | | | | | | | 1.93 | | | | | 2 | | | | | |  |  |  |
| *Pss*-9097 | | | | | | 2.81 | | | | | | 0.09 | | | | 14.97 | | | | | | 2.63 | | | | | | | 2.99 | | | | | 3 | | | | | |  |  |  |
|  | | | | | |  | | | | | |  | | | |  | | | | | |  | | | | | | |  | | | | |  | | | | | |  |  |  |
| **Roundel** | | | | | |  | | | | | |  | | | |  | | | | | |  | | | | | | |  | | | | |  | | | | | |  |  |  |
| strain | | | | | | lsmean | | | | | | SE | | | | df | | | | | | lower.CL | | | | | | | upper.CL | | | | | .group | | | | | |  |  |  |
| Control | | | | | | 0.94 | | | | | | 0.09 | | | | 19.84 | | | | | | 0.75 | | | | | | | 1.13 | | | | | 1 | | | | | |  |  |  |
| R2-5255 | | | | | | 1.72 | | | | | | 0.09 | | | | 17.96 | | | | | | 1.53 | | | | | | | 1.92 | | | | | 2 | | | | | |  |  |  |
| R1-5244 | | | | | | 1.77 | | | | | | 0.08 | | | | 13.77 | | | | | | 1.59 | | | | | | | 1.95 | | | | | 2 | | | | | |  |  |  |
| *Pss*-9097 | | | | | | 3.27 | | | | | | 0.09 | | | | 14.97 | | | | | | 3.08 | | | | | | | 3.45 | | | | | 3 | | | | | |  |  |  |
|  | | | | | |  | | | | | |  | | | |  | | | | | |  | | | | | | |  | | | | |  | | | | | |  |  |  |
| **Van** | | | | | |  | | | | | |  | | | |  | | | | | |  | | | | | | |  | | | | |  | | | | | |  |  |  |
| strain | | | | | | lsmean | | | | | | SE | | | | df | | | | | | lower.CL | | | | | | | upper.CL | | | | | .group | | | | | |  |  |  |
| Control | | | | | | 0.96 | | | | | | 0.09 | | | | 19.84 | | | | | | 0.76 | | | | | | | 1.15 | | | | | 1 | | | | | |  |  |  |
| R2-5255 | | | | | | 1.85 | | | | | | 0.08 | | | | 12.73 | | | | | | 1.67 | | | | | | | 2.03 | | | | | 2 | | | | | |  |  |  |
| R1-5244 | | | | | | 1.92 | | | | | | 0.09 | | | | 14.97 | | | | | | 1.73 | | | | | | | 2.1 | | | | | 2 | | | | | |  |  |  |
| *Pss*-9097 | | | | | | 3.44 | | | | | | 0.08 | | | | 12.73 | | | | | | 3.26 | | | | | | | 3.62 | | | | | 3 | | | | | |  |  |  |
| **Lsmeans (global)** | | | | | | | | | | | | |  | | | | | | |  | | | | |  | | | | | |  | | | | |  | | | |  |  |  |
| strain | cultivar | | | | | | | lsmean | | | | | | SE | | | | | df | | | | | | | lower.CL | | | | | | | upper.CL | | | | .group | | | | |  |
| Control | Napoleon | | | | | | | 0.92 | | | | | | 0.09 | | | | | 14.97 | | | | | | | 0.74 | | | | | | | 1.11 | | | | 1 | | | | |  |
| Control | Roundel | | | | | | | 0.94 | | | | | | 0.09 | | | | | 19.84 | | | | | | | 0.75 | | | | | | | 1.13 | | | | 1 | | | | |  |
| Control | Van | | | | | | | 0.96 | | | | | | 0.09 | | | | | 19.84 | | | | | | | 0.76 | | | | | | | 1.15 | | | | 1 | | | | |  |
| Control | M.Glory | | | | | | | 1.01 | | | | | | 0.08 | | | | | 13.77 | | | | | | | 0.83 | | | | | | | 1.19 | | | | 1 | | | | |  |
| R2-5255 | M.Glory | | | | | | | 1.6 | | | | | | 0.09 | | | | | 14.97 | | | | | | | 1.42 | | | | | | | 1.78 | | | | 2 | | | | |  |
| R1-5244 | M.Glory | | | | | | | 1.62 | | | | | | 0.09 | | | | | 16.35 | | | | | | | 1.43 | | | | | | | 1.81 | | | | 2 | | | | |  |
| R2-5255 | Roundel | | | | | | | 1.72 | | | | | | 0.09 | | | | | 17.96 | | | | | | | 1.53 | | | | | | | 1.92 | | | | 2 | | | | |  |
| R2-5255 | Napoleon | | | | | | | 1.74 | | | | | | 0.09 | | | | | 14.97 | | | | | | | 1.56 | | | | | | | 1.93 | | | | 2 | | | | |  |
| R1-5244 | Napoleon | | | | | | | 1.75 | | | | | | 0.08 | | | | | 13.77 | | | | | | | 1.56 | | | | | | | 1.93 | | | | 2 | | | | |  |
| R1-5244 | Roundel | | | | | | | 1.77 | | | | | | 0.08 | | | | | 13.77 | | | | | | | 1.59 | | | | | | | 1.95 | | | | 2 | | | | |  |
| R2-5255 | Van | | | | | | | 1.85 | | | | | | 0.08 | | | | | 12.73 | | | | | | | 1.67 | | | | | | | 2.03 | | | | 2 | | | | |  |
| R1-5244 | Van | | | | | | | 1.92 | | | | | | 0.09 | | | | | 14.97 | | | | | | | 1.73 | | | | | | | 2.1 | | | | 2 | | | | |  |
| *Pss*-9097 | Napoleon | | | | | | | 2.81 | | | | | | 0.09 | | | | | 14.97 | | | | | | | 2.63 | | | | | | | 2.99 | | | | 3 | | | | |  |
| *Pss*-9097 | M.Glory | | | | | | | 3.04 | | | | | | 0.08 | | | | | 12.73 | | | | | | | 2.86 | | | | | | | 3.22 | | | | 34 | | | | |  |
| *Pss*-9097 | Roundel | | | | | | | 3.27 | | | | | | 0.09 | | | | | 14.97 | | | | | | | 3.08 | | | | | | | 3.45 | | | | 45 | | | | |  |
| *Pss*-9097 | Van | | | | | | | 3.44 | | | | | | 0.08 | | | | | 12.73 | | | | | | | 3.26 | | | | | | | 3.62 | | | | 5 | | | | |  |
| **Lsmeans Cultivars only** | | | | | | | | | | | | | | | | | | | | | | | | | | | | | | | | | | | | | | | | | | |
| cultivar | | | | | lsmean | | | | | | SE | | | | | | | df | | | | | | lower.CL | | | | | | | | upper.CL | | | | | | .group | | | | |
| M.Glory | | | | 1.8 | | | | | | 0.06 | | | | | | | 67.32 | | | | | | | 1.69 | | | | | | | | 1.92 | | | | | | 1 | | | | |
| Napoleon | | | | 1.83 | | | | | | 0.06 | | | | | | | 66.76 | | | | | | | 1.71 | | | | | | | | 1.94 | | | | | | 1 | | | | |
| Roundel | | | | 1.96 | | | | | | 0.06 | | | | | | | 79.67 | | | | | | | 1.83 | | | | | | | | 2.09 | | | | | | 12 | | | | |
| Van | | | | 2.12 | | | | | | 0.06 | | | | | | | 69.92 | | | | | | | 2 | | | | | | | | 2.24 | | | | | | 2 | | | | |
|  | |  | | | | | | |  | | | | | |  | | | | | | | |  | | | | | | |  | | | | |  | | | | | | |  |

**Table S16: REML analysis of immature cherry fruit inoculations where different bacterial strains were inoculated onto different host cultivars.** The REML model is presented. Lsmeans Tukey-HSD groups for strains on different cultivars are presented (corresponds to groupings on Figure 6B), followed by groupings based on all possible treatments and then a comparison of cultivars only.
