# Supplementary material for: Characterization of the pathogenicity of strains of Pseudomonas syringae towards cherry and plum
Source: Plant Pathol. 2018 Feb 14;67(5):1177–93. doi: 10.1111/ppa.12834 (PMC5993217; doi:10.1111/ppa.12834)
Supplement: Supplementary file 25 — Table S17. ANOVA table of day‐10 leaf population counts of different bacterial strains inoculated on cherry and plum. [file PPA-67-1177-s025.docx]

| **Cherry leaves over time** | | | | | | |
| --- | --- | --- | --- | --- | --- | --- |
| **ANOVA** |  |  |  |  |  |  |
|  | Df | Sum Sq | Mean Sq | F.value | Pr(>F) |  |
| strain | 6 | 1198.1 | 199.69 | 97.13 | <2.00E-16 | *** |
| leaf | 2 | 22.1 | 11.06 | 5.38 | 0.008 | ** |
| leaf:rep | 6 | 1.1 | 0.18 | 0.09 | 1 |  |
| Residuals | 48 | 98.7 | 2.06 |  |  |  |
|  |  |  |  |  |  |  |
| **Groups** |  |  |  |  |  |  |
| strain | lsmean | SE | df | lower.CL | upper.CL | .group |
| *Pph* | 14.47 | 0.48 | 48 | 13.51 | 15.43 | 1 |
| RMA1 | 14.8 | 0.48 | 48 | 13.84 | 15.76 | 1 |
| *Psv* | 16.13 | 0.48 | 48 | 15.17 | 17.09 | 1 |
| R1-5300 | 19.15 | 0.48 | 48 | 18.19 | 20.11 | 2 |
| *Pss*-9097 | 23.31 | 0.48 | 48 | 22.35 | 24.27 | 3 |
| R1-5244 | 24.92 | 0.48 | 48 | 23.96 | 25.88 | 3 |
| R2-leaf | 25.04 | 0.48 | 48 | 24.08 | 26 | 3 |
|  | | | | | | |
| **Plum leaves over time** | | | | | | |
| **ANOVA** |  |  |  |  |  |  |
|  | Df | Sum Sq | Mean Sq | F.value | Pr(>F) |  |
| strain | 6 | 1371.9 | 228.65 | 67.71 | <2e-16 | *** |
| leaf | 2 | 19.3 | 9.65 | 2.86 | 0.0672 | . |
| leaf:rep | 6 | 0.4 | 0.07 | 0.02 | 1 |  |
| Residuals | 48 | 162.1 | 3.38 |  |  |  |
|  |  |  |  |  |  |  |
| **Groups** |  |  |  |  |  |  |
| strain | lsmean | SE | df | lower.CL | upper.CL | .group |
| *Pph* | 12.06 | 0.61 | 48 | 10.83 | 13.3 | 1 |
| *Psv* | 14.64 | 0.61 | 48 | 13.41 | 15.87 | 1 |
| RMA1 | 21.64 | 0.61 | 48 | 20.41 | 22.87 | 2 |
| R1-5244 | 22.51 | 0.61 | 48 | 21.28 | 23.74 | 23 |
| R2-leaf | 22.85 | 0.61 | 48 | 21.62 | 24.08 | 23 |
| R1-5300 | 24.41 | 0.61 | 48 | 23.18 | 25.64 | 3 |
| *Pss*-9097 | 25.06 | 0.61 | 48 | 23.83 | 26.29 | 3 |

**Table S17: ANOVA table of day 10 leaf population counts of different bacterial strains inoculated on cherry and plum.** Tukey-HSD groups for strains are presented (corresponds to groupings on Figure 7).
