# Supplementary material for: Characterization of the pathogenicity of strains of Pseudomonas syringae towards cherry and plum
Source: Plant Pathol. 2018 Feb 14;67(5):1177–93. doi: 10.1111/ppa.12834 (PMC5993217; doi:10.1111/ppa.12834)
Supplement: Supplementary file 26 — Table S18. REML analysis of day‐10 leaf population counts of reference bacterial strains inoculated on cherry leaves. [file PPA-67-1177-s026.docx]

| Model: lmer(log2(cfu) ~ strain + (1\|exp/leaf/rep)) | | | | |  |  |  |
| --- | --- | --- | --- | --- | --- | --- | --- |
| **ANOVA** | | | | |  |  |  |
|  | Sum Sq | Mean Sq | NumDF | DenDF | F.value | Pr(>F) |  |
| strain | 1920.3 | 240.04 | 8 | 130.19 | 105.71 | <2.20E-16 | *** |
| **Lsmeans** |  |  | | |  |  |  |
| strain | lsmean | SE | df | lower.CL | upper.CL | .group |  |
| RMA1 | 14.96 | 0.6 | 2.04 | 12.43 | 17.49 | 1 |  |
| *Pph* | 16.52 | 0.6 | 2.04 | 13.99 | 19.05 | 12 |  |
| *Psv* | 17.44 | 0.6 | 2.04 | 14.91 | 19.97 | 2 |  |
| *Ps*-9643 | 18.43 | 0.71 | 3.93 | 16.44 | 20.41 | 23 |  |
| R1-5300 | 19.25 | 0.6 | 2.04 | 16.72 | 21.78 | 3 |  |
| *Pss*-9293 | 22.04 | 0.71 | 3.93 | 20.06 | 24.02 | 4 |  |
| *Pss*-9097 | 23.6 | 0.6 | 2.04 | 21.07 | 26.13 | 45 |  |
| R1-5244 | 24.39 | 0.6 | 2.04 | 21.87 | 26.92 | 5 |  |
| R2-leaf | 25.17 | 0.6 | 2.04 | 22.65 | 27.7 | 5 |  |

**Table S18: REML analysis of day 10 leaf population counts of reference bacterial strains inoculated on cherry leaves.** The model is shown followed by ANOVA table. Lsmeans Tukey-HSD groups for strains are presented (corresponds to groupings on Figure 8A).
