# Supplementary material for: Characterization of the pathogenicity of strains of Pseudomonas syringae towards cherry and plum
Source: Plant Pathol. 2018 Feb 14;67(5):1177–93. doi: 10.1111/ppa.12834 (PMC5993217; doi:10.1111/ppa.12834)
Supplement: Supplementary file 27 — Table S19. REML analysis of day‐10 leaf population counts of reference bacterial strains inoculated on plum leaves. [file PPA-67-1177-s027.docx]

| **Model: lmer(log2(cfu) ~ strain + (1\|exp/leaf/rep))** | | | | |  |  |  |
| --- | --- | --- | --- | --- | --- | --- | --- |
|  |  |  |  |  |  |  |  |
| **ANOVA** |  |  |  |  |  |  |  |
|  | Sum Sq | Mean Sq | NumDF | DenDF | F.value | Pr(>F) |  |
| strain | 1462.7 | 182.84 | 8 | 130 | 49.58 | <2.20E-16 | *** |
| **Lsmeans** |  |  |  |  |  |  |  |
| strain | lsmean | SE | df | lower.CL | upper.CL | .group |  |
| *Pph* | 16.72 | 1.82 | 1.11 | -1.51 | 34.96 | 1 |  |
| *Psv* | 17.48 | 1.82 | 1.11 | -0.75 | 35.72 | 1 |  |
| *Ps*-9643 | 20.97 | 1.89 | 1.28 | 6.37 | 35.57 | 2 |  |
| RMA1 | 22.65 | 1.82 | 1.11 | 4.42 | 40.89 | 23 |  |
| *Pss*-9293 | 23.15 | 1.89 | 1.28 | 8.55 | 37.75 | 234 |  |
| R1-5244 | 23.61 | 1.82 | 1.11 | 5.38 | 41.85 | 34 |  |
| R2-leaf | 24.25 | 1.82 | 1.11 | 6.01 | 42.48 | 345 |  |
| R1-5300 | 25.04 | 1.82 | 1.11 | 6.81 | 43.28 | 45 |  |
| *Pss*-9097 | 25.85 | 1.82 | 1.11 | 7.62 | 44.09 | 5 |  |

**Table S19: REML analysis of day 10 leaf population counts of reference bacterial strains inoculated on plum leaves.** The model is shown followed by ANOVA table. Lsmeans Tukey-HSD groups for strains are presented (corresponds to groupings on Figure 8A).
