# Supplementary material for: Characterization of the pathogenicity of strains of Pseudomonas syringae towards cherry and plum
Source: Plant Pathol. 2018 Feb 14;67(5):1177–93. doi: 10.1111/ppa.12834 (PMC5993217; doi:10.1111/ppa.12834)
Supplement: Supplementary file 28 — Table S20. ANOVA table of day‐10 leaf population counts of different bacterial strains inoculated on different cherry cultivars. [file PPA-67-1177-s028.docx]

| **ANOVA** |  |  |  |  |  |  |
| --- | --- | --- | --- | --- | --- | --- |
|  | Df | Sum Sq | Mean Sq | F value | Pr(>F) |  |
| strain | 2 | 40.9 | 20.43 | 6.95 | 0.001 | ** |
| cultivar | 3 | 49.7 | 16.58 | 5.64 | 0.001 | ** |
| exp | 1 | 67.9 | 67.91 | 23.09 | 3.16E-06 | *** |
| strain:cultivar | 6 | 97.3 | 16.22 | 5.52 | 2.78E-05 | *** |
| exp:leaf | 4 | 12.8 | 3.21 | 1.09 | 0.36 |  |
| exp:leaf:rep | 12 | 3.1 | 0.26 | 0.09 | 1 |  |
| Residuals | 187 | 550 | 2.94 |  |  |  |
| **Lsmeans (strains on different cultivars)** | | | | |  |  |
| **Merton Glory** | |  |  |  |  |  |
| strain | lsmean | SE | df | lower.CL | upper.CL | .group |
| R2-leaf | 21.77 | 0.4 | 187 | 20.98 | 22.57 | 1 |
| *Pss*-9097 | 21.81 | 0.4 | 187 | 21.01 | 22.61 | 1 |
| R1-5244 | 21.89 | 0.4 | 187 | 21.09 | 22.68 | 1 |
|  |  |  |  |  |  |  |
| **Napoleon** |  |  |  |  |  |  |
| strain | lsmean | SE | df | lower.CL | upper.CL | .group |
| *Pss*-9097 | 21.45 | 0.4 | 187 | 20.65 | 22.25 | 1 |
| R1-5244 | 22.47 | 0.4 | 187 | 21.68 | 23.27 | 1 |
| R2-leaf | 24.78 | 0.4 | 187 | 23.99 | 25.58 | 2 |
|  |  |  |  |  |  |  |
| **Roundel** |  |  |  |  |  |  |
| strain | lsmean | SE | df | lower.CL | upper.CL | .group |
| R1-5244 | 21.1 | 0.4 | 187 | 20.31 | 21.9 | 1 |
| *Pss*-9097 | 21.25 | 0.4 | 187 | 20.45 | 22.04 | 1 |
| R2-leaf | 22.6 | 0.4 | 187 | 21.81 | 23.4 | 2 |
|  |  |  |  |  |  |  |
| **Van** |  |  |  |  |  |  |
| strain | lsmean | SE | df | lower.CL | upper.CL | .group |
| R2-leaf | 21.68 | 0.4 | 187 | 20.88 | 22.47 | 1 |
| *Pss*-9097 | 22.13 | 0.4 | 187 | 21.33 | 22.92 | 1 |
| R1-5244 | 22.65 | 0.4 | 187 | 21.85 | 23.45 | 1 |
|  |  |  |  |  |  |  |
| **Lsmeans (different cultivars)** | | |  |  |  |  |
| cultivar | lsmean | SE | df | lower.CL | upper.CL | .group |
| Roundel | 21.65 | 0.23 | 187 | 21.19 | 22.11 | 1 |
| Merton Glory | 21.82 | 0.23 | 187 | 21.36 | 22.28 | 1 |
| Van | 22.15 | 0.23 | 187 | 21.69 | 22.61 | 12 |
| Napoleon | 22.9 | 0.23 | 187 | 22.44 | 23.36 | 2 |

**Table S20: ANOVA table of day 10 leaf population counts of different bacterial strains inoculated on different cherry cultivars.** Tukey-HSD groups for strains on different cultivars are presented (corresponds to groupings on Figure 8C), followed by a comparison of cultivars only.
