# Supplementary material for: Characterization of the pathogenicity of strains of Pseudomonas syringae towards cherry and plum
Source: Plant Pathol. 2018 Feb 14;67(5):1177–93. doi: 10.1111/ppa.12834 (PMC5993217; doi:10.1111/ppa.12834)
Supplement: Supplementary file 29 — Table S21. ANOVA table of leaf population counts of all strains used in this study. [file PPA-67-1177-s029.docx]

| **ANOVA** |  |  |  |  |  |  |
| --- | --- | --- | --- | --- | --- | --- |
|  | Df | Sum Sq | Mean Sq | F.value | Pr(>F) |  |
| strain | 20 | 2465.6 | 123.28 | 64.07 | <2.00E-16 | *** |
| leaf | 2 | 31.8 | 15.9 | 8.26 | 0.0004 | *** |
| leaf:rep | 6 | 0.2 | 0.03 | 0.01 | 1 |  |
| Residuals | 160 | 307.9 | 1.92 |  |  |  |
|  |  |  |  |  |  |  |
| **Groups** |  |  |  |  |  |  |
| strain | lsmean | SE | df | lower.CL | upper.CL | .group |
| RMA1 | 14.35 | 0.46 | 160 | 13.44 | 15.27 | 1 |
| *Ps*-9643 | 17.86 | 0.46 | 160 | 16.95 | 18.78 | 2 |
| *Pph* | 18.39 | 0.46 | 160 | 17.48 | 19.3 | 2 |
| *Psv* | 21.95 | 0.46 | 160 | 21.04 | 22.86 | 3 |
| R1-5300 | 22.07 | 0.46 | 160 | 21.16 | 22.98 | 3 |
| R1-9629 | 22.17 | 0.46 | 160 | 21.26 | 23.08 | 3 |
| R1-9326 | 22.31 | 0.46 | 160 | 21.4 | 23.22 | 3 |
| R1-9657 | 23.11 | 0.46 | 160 | 22.2 | 24.03 | 3 |
| R2-5260 | 25.75 | 0.46 | 160 | 24.83 | 26.66 | 4 |
| *Pss*-9293 | 26.05 | 0.46 | 160 | 25.14 | 26.96 | 4 |
| R2-5255 | 26.17 | 0.46 | 160 | 25.25 | 27.08 | 4 |
| *Pss*-9644 | 26.21 | 0.46 | 160 | 25.3 | 27.13 | 4 |
| *Pss*-9654 | 26.24 | 0.46 | 160 | 25.33 | 27.15 | 4 |
| R1-9646 | 26.34 | 0.46 | 160 | 25.43 | 27.26 | 4 |
| *Pss*-9097 | 26.87 | 0.46 | 160 | 25.96 | 27.79 | 4 |
| R2-leaf | 27 | 0.46 | 160 | 26.09 | 27.91 | 4 |
| *Pss*-9659 | 27.05 | 0.46 | 160 | 26.13 | 27.96 | 4 |
| *Pss*-9656 | 27.25 | 0.46 | 160 | 26.34 | 28.16 | 4 |
| *Pss*-9630 | 27.27 | 0.46 | 160 | 26.36 | 28.19 | 4 |
| R1-5244 | 27.38 | 0.46 | 160 | 26.47 | 28.3 | 4 |
| R2-SC214 | 27.39 | 0.46 | 160 | 26.48 | 28.31 | 4 |

**Table S21: ANOVA table of leaf population counts of all strains used in this study**, followed by Tukey-HSD groupings of the strains (corresponds to groupings on Figure S6).
