# Supplementary material for: Characterization of the pathogenicity of strains of Pseudomonas syringae towards cherry and plum
Source: Plant Pathol. 2018 Feb 14;67(5):1177–93. doi: 10.1111/ppa.12834 (PMC5993217; doi:10.1111/ppa.12834)
Supplement: Supplementary file 30 — Table S22. ANOVA table of AUDPC analysis of symptom score on leaves of several bacterial strains inoculated at different concentrations. [file PPA-67-1177-s030.docx]

| **ANOVA** |  |  |  |  |  |  |
| --- | --- | --- | --- | --- | --- | --- |
|  | Df | Sum Sq | Mean Sq | F value | Pr(>F) |  |
| strain | 4 | 82195 | 20549 | 182.17 | <2.00E-16 | *** |
| conc | 3 | 21514 | 7171 | 63.57 | <2.00E-16 | *** |
| strain:conc | 12 | 17222 | 1435 | 12.72 | 3.97E-11 | *** |
| conc:leaf | 12 | 1498 | 125 | 1.11 | 0.38 |  |
| Residuals | 48 | 5414 | 113 |  |  |  |
|  |  |  |  |  |  |  |
| **2.5x10^7^** |  |  |  |  |  |  |
| strain | lsmean | SE | df | lower.CL | upper.CL | .group |
| R1-5244 | -1.05E-13 | 5.31 | 48 | -10.68 | 10.68 | 1 |
| R1-5300 | 5.18E-14 | 5.31 | 48 | -10.68 | 10.68 | 1 |
| RMA1 | 5.18E-14 | 5.31 | 48 | -10.68 | 10.68 | 1 |
| R2-leaf | 6.00E+00 | 5.31 | 48 | -4.68 | 16.68 | 1 |
| *Pss*-9097 | 9.00E+01 | 5.31 | 48 | 79.32 | 100.68 | 2 |
|  |  |  |  |  |  |  |
| **5x10^7^** |  |  |  |  |  |  |
| strain | lsmean | SE | df | lower.CL | upper.CL | .group |
| R1-5244 | -1.40E-14 | 5.31 | 48 | -10.68 | 10.68 | 1 |
| RMA1 | 1.20E+01 | 5.31 | 48 | 1.32 | 22.68 | 12 |
| R2-leaf | 1.20E+01 | 5.31 | 48 | 1.32 | 22.68 | 12 |
| R1-5300 | 2.40E+01 | 5.31 | 48 | 13.32 | 34.68 | 2 |
| *Pss*-9097 | 8.40E+01 | 5.31 | 48 | 73.32 | 94.68 | 3 |
|  |  |  |  |  |  |  |
| **1x10^8^** |  |  |  |  |  |  |
| strain | lsmean | SE | df | lower.CL | upper.CL | .group |
| R1-5244 | -1.91E-14 | 5.31 | 48 | -10.68 | 10.68 | 1 |
| R2-leaf | 6.00E+00 | 5.31 | 48 | -4.68 | 16.68 | 12 |
| RMA1 | 2.40E+01 | 5.31 | 48 | 13.32 | 34.68 | 2 |
| R1-5300 | 6.00E+01 | 5.31 | 48 | 49.32 | 70.68 | 3 |
| *Pss*-9097 | 1.02E+02 | 5.31 | 48 | 91.32 | 112.68 | 4 |
|  |  |  |  |  |  |  |
| **2x10^8^** |  |  |  |  |  |  |
| strain | lsmean | SE | df | lower.CL | upper.CL | .group |
| R1-5244 | -1.80E-14 | 5.31 | 48 | -10.68 | 10.68 | 1 |
| R2-leaf | 4.20E+01 | 5.31 | 48 | 31.32 | 52.68 | 2 |
| R1-5300 | 7.80E+01 | 5.31 | 48 | 67.32 | 88.68 | 3 |
| RMA1 | 9.00E+01 | 5.31 | 48 | 79.32 | 100.68 | 34 |
| *Pss*-9097 | 1.02E+02 | 5.31 | 48 | 91.32 | 112.68 | 4 |

**Table S22: ANOVA table of AUDPC analysis of symptom score on leaves of several bacterial strains inoculated at different concentrations.** This is followed by lsmeans Tukey-HSD groupings for the strains at different concentrations (corresponds to groupings on Figure 9A).
