# Supplementary material for: Characterization of the pathogenicity of strains of Pseudomonas syringae towards cherry and plum
Source: Plant Pathol. 2018 Feb 14;67(5):1177–93. doi: 10.1111/ppa.12834 (PMC5993217; doi:10.1111/ppa.12834)
Supplement: Supplementary file 31 — Table S23. ANOVA table of AUDPC analysis of leaf symptom score over time of different bacterial strains inoculated on cherry. [file PPA-67-1177-s031.docx]

| **ANOVA** |  |  |  |  |  |  |
| --- | --- | --- | --- | --- | --- | --- |
|  | Df | Sum Sq | Mean Sq | F value | Pr(>F) |  |
| strain | 8 | 34992 | 4374 | 13.05 | 2.09E-10 | *** |
| exp | 1 | 16928 | 16928 | 50.51 | 2.30E-09 | *** |
| exp:leaf | 6 | 1888 | 315 | 0.94 | 0.46 |  |
| Residuals | 56 | 18768 | 335 |  |  |  |
| **Groups** |  |  |  |  |  |  |
| trt | means |  |  |  |  |  |
| *Ps*-9643 | 78 | a |  |  |  |  |
| *Pss*-9097 | 78 | a |  |  |  |  |
| *Pss*-9293 | 69 | ab |  |  |  |  |
| RMA1 | 66 | ab |  |  |  |  |
| R1-5300 | 48 | bc |  |  |  |  |
| *Psv*BP631 | 45 | bc |  |  |  |  |
| *Pph* | 42 | bc |  |  |  |  |
| R2-leaf | 36 | c |  |  |  |  |
| R1-5244 | 6 | d |  |  |  |  |

**Table S23: ANOVA table of AUDPC analysis of leaf symptom score over time of different bacterial strains inoculated on cherry.** Tukey-HSD groups for strains are presented (corresponds to groupings on Figure 9B).
