# Supplementary material for: Characterization of the pathogenicity of strains of Pseudomonas syringae towards cherry and plum
Source: Plant Pathol. 2018 Feb 14;67(5):1177–93. doi: 10.1111/ppa.12834 (PMC5993217; doi:10.1111/ppa.12834)
Supplement: Supplementary file 32 — Table S24. ANOVA table of AUDPC analysis of leaf symptom score over time of different bacterial strains inoculated on plum. [file PPA-67-1177-s032.docx]

| **ANOVA** |  |  |  |  |  |  |
| --- | --- | --- | --- | --- | --- | --- |
|  | Df | Sum Sq | Mean Sq | F value | Pr(>F) |  |
| strain | 8 | 28336 | 3542 | 12.14 | 7.13E-10 | *** |
| exp | 1 | 38088 | 38088 | 130.57 | 2.93E-16 | *** |
| exp:leaf | 6 | 10160 | 1693 | 5.81 | 9.40E-05 | *** |
| Residuals | 56 | 16336 | 292 |  |  |  |
| **Groups** |  |  |  |  |  |  |
| trt | means |  |  |  |  |  |
| *Pss*-9293 | 84 | a |  |  |  |  |
| *Pss*-9097 | 75 | ab |  |  |  |  |
| *Ps*-9643 | 66 | ab |  |  |  |  |
| *Psv* | 54 | bc |  |  |  |  |
| RMA1 | 54 | bc |  |  |  |  |
| *Pph* | 48 | bcd |  |  |  |  |
| R2-leaf | 33 | cd |  |  |  |  |
| R1-5300 | 27 | cd |  |  |  |  |
| R1-5244 | 24 | d |  |  |  |  |

**Table S24: ANOVA table of AUDPC analysis of leaf symptom score over time of different bacterial strains inoculated on plum.** Tukey-HSD groups for strains are presented (corresponds to groupings on Figure 9B).
