# Supplementary material for: Characterization of the pathogenicity of strains of Pseudomonas syringae towards cherry and plum
Source: Plant Pathol. 2018 Feb 14;67(5):1177–93. doi: 10.1111/ppa.12834 (PMC5993217; doi:10.1111/ppa.12834)
Supplement: Supplementary file 33 — Table S25. AUDPC values based on symptom development 0–48 h after inoculation for several bacterial strains on cherry leaves inoculated at different starting concentrations (0.5 × 10^7^, 107, 108, 2 × 10^8^ CFU mL−1). [file PPA-67-1177-s033.docx]

| Concentration | Leaf | Strain | AUDPC |
| --- | --- | --- | --- |
| 1 | 1 | *Pss*-9097 | 72 |
| 1 | 2 | *Pss*-9097 | 96 |
| 1 | 3 | *Pss*-9097 | 96 |
| 1 | 4 | *Pss*-9097 | 96 |
| 1 | 1 | R1-5244 | 0 |
| 1 | 2 | R1-5244 | 0 |
| 1 | 3 | R1-5244 | 0 |
| 1 | 4 | R1-5244 | 0 |
| 1 | 1 | R1-5300 | 0 |
| 1 | 2 | R1-5300 | 0 |
| 1 | 3 | R1-5300 | 0 |
| 1 | 4 | R1-5300 | 0 |
| 1 | 1 | R2-leaf | 24 |
| 1 | 2 | R2-leaf | 0 |
| 1 | 3 | R2-leaf | 0 |
| 1 | 4 | R2-leaf | 0 |
| 1 | 1 | RMA1 | 0 |
| 1 | 2 | RMA1 | 0 |
| 1 | 3 | RMA1 | 0 |
| 1 | 4 | RMA1 | 0 |
| 2 | 1 | *Pss*-9097 | 96 |
| 2 | 2 | *Pss*-9097 | 96 |
| 2 | 3 | *Pss*-9097 | 72 |
| 2 | 4 | *Pss*-9097 | 72 |
| 2 | 1 | R1-5244 | 0 |
| 2 | 2 | R1-5244 | 0 |
| 2 | 3 | R1-5244 | 0 |
| 2 | 4 | R1-5244 | 0 |
| 2 | 1 | R1-5300 | 24 |
| 2 | 2 | R1-5300 | 24 |
| 2 | 3 | R1-5300 | 24 |
| 2 | 4 | R1-5300 | 24 |
| 2 | 1 | R2-leaf | 24 |
| 2 | 2 | R2-leaf | 24 |
| 2 | 3 | R2-leaf | 0 |
| 2 | 4 | R2-leaf | 0 |
| 2 | 1 | RMA1 | 24 |
| 2 | 2 | RMA1 | 0 |
| 2 | 3 | RMA1 | 0 |
| 2 | 4 | RMA1 | 24 |
| 3 | 1 | *Pss*-9097 | 120 |
| 3 | 2 | *Pss*-9097 | 96 |
| 3 | 3 | *Pss*-9097 | 96 |
| 3 | 4 | *Pss*-9097 | 96 |
| 3 | 1 | R1-5244 | 0 |
| 3 | 2 | R1-5244 | 0 |
| 3 | 3 | R1-5244 | 0 |
| 3 | 4 | R1-5244 | 0 |
| 3 | 1 | R1-5300 | 72 |
| 3 | 2 | R1-5300 | 48 |
| 3 | 3 | R1-5300 | 48 |
| 3 | 4 | R1-5300 | 72 |
| 3 | 1 | R2-leaf | 24 |
| 3 | 2 | R2-leaf | 0 |
| 3 | 3 | R2-leaf | 0 |
| 3 | 4 | R2-leaf | 0 |
| 3 | 1 | RMA1 | 24 |
| 3 | 2 | RMA1 | 24 |
| 3 | 3 | RMA1 | 24 |
| 3 | 4 | RMA1 | 24 |
| 4 | 1 | *Pss*-9097 | 96 |
| 4 | 2 | *Pss*-9097 | 96 |
| 4 | 3 | *Pss*-9097 | 96 |
| 4 | 4 | *Pss*-9097 | 120 |
| 4 | 1 | R1-5244 | 0 |
| 4 | 2 | R1-5244 | 0 |
| 4 | 3 | R1-5244 | 0 |
| 4 | 4 | R1-5244 | 0 |
| 4 | 1 | R1-5300 | 96 |
| 4 | 2 | R1-5300 | 72 |
| 4 | 3 | R1-5300 | 72 |
| 4 | 4 | R1-5300 | 72 |
| 4 | 1 | R2-leaf | 48 |
| 4 | 2 | R2-leaf | 72 |
| 4 | 3 | R2-leaf | 24 |
| 4 | 4 | R2-leaf | 24 |
| 4 | 1 | RMA1 | 72 |
| 4 | 2 | RMA1 | 96 |
| 4 | 3 | RMA1 | 96 |
| 4 | 4 | RMA1 | 96 |

**Table S25: AUDPC values based on symptom development 0-48 hours after inoculation for several bacterial strains on cherry leaves inoculated at different starting concentrations (0.5x10^7^, 1.0x10^7^, 1.0x10^8^, 2.0x10^8^ CFU/ml).**
