# Supplementary material for: Characterization of the pathogenicity of strains of Pseudomonas syringae towards cherry and plum
Source: Plant Pathol. 2018 Feb 14;67(5):1177–93. doi: 10.1111/ppa.12834 (PMC5993217; doi:10.1111/ppa.12834)
Supplement: Supplementary file 36 — Table S28. Data used in correlation analysis for Figure 10. [file PPA-67-1177-s036.docx]

| **Experiment** | **Strain** | **Replicate** | **Original data** | **Standardised** |
| --- | --- | --- | --- | --- |
| Cut shoot inoculation | Cherry *Psm* R1 | 1 | 13.45 | 0.04 |
| Cut shoot inoculation | Cherry *Psm* R1 | 2 | 23.42 | 0.61 |
| Cut shoot inoculation | Cherry *Psm* R1 | 3 | 1.29 | -0.66 |
| Cut shoot inoculation | Cherry *Psm* R1 | 4 | 9.14 | -0.21 |
| Cut shoot inoculation | Cherry *Psm* R1 | 5 | 3.25 | -0.55 |
| Cut shoot inoculation | Cherry *Psm* R1 | 6 | 56.98 | 2.53 |
| Cut shoot inoculation | Cherry *Psm* R1 | 7 | 51.51 | 2.22 |
| Cut shoot inoculation | Cherry *Psm* R1 | 8 | 25.67 | 0.74 |
| Cut shoot inoculation | Cherry *Psm* R1 | 9 | 29.31 | 0.95 |
| Cut shoot inoculation | Cherry *Psm* R1 | 10 | 43.72 | 1.77 |
| Cut shoot inoculation | Cherry *Psm* R2 | 1 | 7.38 | -0.31 |
| Cut shoot inoculation | Cherry *Psm* R2 | 2 | 5.88 | -0.39 |
| Cut shoot inoculation | Cherry *Psm* R2 | 3 | 3.37 | -0.54 |
| Cut shoot inoculation | Cherry *Psm* R2 | 4 | 15.31 | 0.15 |
| Cut shoot inoculation | Cherry *Psm* R2 | 5 | 4.68 | -0.46 |
| Cut shoot inoculation | Cherry *Psm* R2 | 6 | 23 | 0.59 |
| Cut shoot inoculation | Cherry *Psm* R2 | 7 | 14.29 | 0.09 |
| Cut shoot inoculation | Cherry *Psm* R2 | 8 | 6.89 | -0.34 |
| Cut shoot inoculation | Cherry *Psm* R2 | 9 | 18.66 | 0.34 |
| Cut shoot inoculation | Cherry *Psm* R2 | 10 | 3.54 | -0.53 |
| Cut shoot inoculation | Plum *Psm* R1 | 1 | 4.3 | -0.48 |
| Cut shoot inoculation | Plum *Psm* R1 | 2 | 1.12 | -0.67 |
| Cut shoot inoculation | Plum *Psm* R1 | 3 | 7.35 | -0.31 |
| Cut shoot inoculation | Plum *Psm* R1 | 4 | 3.23 | -0.55 |
| Cut shoot inoculation | Plum *Psm* R1 | 5 | 1.13 | -0.67 |
| Cut shoot inoculation | Plum *Psm* R1 | 6 | 4.24 | -0.49 |
| Cut shoot inoculation | Plum *Psm* R1 | 7 | 3.35 | -0.54 |
| Cut shoot inoculation | Plum *Psm* R1 | 8 | 5.63 | -0.41 |
| Cut shoot inoculation | Plum *Psm* R1 | 9 | 0.59 | -0.7 |
| Cut shoot inoculation | Plum *Psm* R1 | 10 | 3.7 | -0.52 |
| Cut shoot inoculation | Plum *Pss* | 1 | 2.13 | -0.61 |
| Cut shoot inoculation | Plum *Pss* | 2 | 4.01 | -0.5 |
| Cut shoot inoculation | Plum *Pss* | 3 | 1.03 | -0.67 |
| Cut shoot inoculation | Plum *Pss* | 4 | 6.18 | -0.38 |
| Cut shoot inoculation | Plum *Pss* | 5 | 13.5 | 0.04 |
| Cut shoot inoculation | Plum *Pss* | 6 | 11.26 | -0.09 |
| Cut shoot inoculation | Plum *Pss* | 7 | 5.73 | -0.4 |
| Cut shoot inoculation | Plum *Pss* | 8 | 2.53 | -0.59 |
| Cut shoot inoculation | Plum *Pss* | 9 | 1.65 | -0.64 |
| Cut shoot inoculation | Plum *Pss* | 10 | 6.11 | -0.38 |
| Cut shoot inoculation | Cherry *Pss* | 1 | 21.46 | 0.5 |
| Cut shoot inoculation | Cherry *Pss* | 2 | 23.21 | 0.6 |
| Cut shoot inoculation | Cherry *Pss* | 3 | 2.15 | -0.61 |
| Cut shoot inoculation | Cherry *Pss* | 4 | 14 | 0.07 |
| Cut shoot inoculation | Cherry *Pss* | 5 | 70.24 | 3.29 |
| Cut shoot inoculation | Cherry *Pss* | 6 | 12.28 | -0.03 |
| Cut shoot inoculation | Cherry *Pss* | 7 | 52.83 | 2.3 |
| Cut shoot inoculation | Cherry *Pss* | 8 | 4.39 | -0.48 |
| Cut shoot inoculation | Cherry *Pss* | 9 | 69.37 | 3.24 |
| Cut shoot inoculation | Cherry *Pss* | 10 | 62.21 | 2.83 |
| Cut shoot inoculation | *Pph* | 1 | 9.64 | -0.18 |
| Cut shoot inoculation | *Pph* | 2 | 2.1 | -0.61 |
| Cut shoot inoculation | *Pph* | 3 | 0.81 | -0.69 |
| Cut shoot inoculation | *Pph* | 4 | 1.36 | -0.65 |
| Cut shoot inoculation | *Pph* | 5 | 1.75 | -0.63 |
| Cut shoot inoculation | *Pph* | 6 | 1.26 | -0.66 |
| Cut shoot inoculation | *Pph* | 7 | 2.4 | -0.59 |
| Cut shoot inoculation | *Pph* | 8 | 0.39 | -0.71 |
| Cut shoot inoculation | *Pph* | 9 | 0.08 | -0.73 |
| Cut shoot inoculation | *Pph* | 10 | 1.52 | -0.64 |
| Cut shoot inoculation | RMA1 | 1 | 1.83 | -0.63 |
| Cut shoot inoculation | RMA1 | 2 | 2.78 | -0.57 |
| Cut shoot inoculation | RMA1 | 3 | 5.59 | -0.41 |
| Cut shoot inoculation | RMA1 | 4 | 9.27 | -0.2 |
| Cut shoot inoculation | RMA1 | 5 | 5.89 | -0.39 |
| Cut shoot inoculation | RMA1 | 6 | 4.97 | -0.45 |
| Cut shoot inoculation | RMA1 | 7 | 1.88 | -0.62 |
| Cut shoot inoculation | RMA1 | 8 | 15.09 | 0.13 |
| Cut shoot inoculation | RMA1 | 9 | 0.28 | -0.72 |
| Cut shoot inoculation | RMA1 | 10 | 42.83 | 1.72 |
| Detached immature fruit | Plum *Psm* R1 | 1 | 1.89 | -0.57 |
| Detached immature fruit | Plum *Psm* R1 | 2 | 1.88 | -0.57 |
| Detached immature fruit | Plum *Psm* R1 | 3 | 2.05 | -0.53 |
| Detached immature fruit | Plum *Psm* R1 | 4 | 2.33 | -0.46 |
| Detached immature fruit | Plum *Psm* R1 | 5 | 1.6 | -0.64 |
| Detached immature fruit | Cherry *Psm* R1 | 1 | 2.91 | -0.31 |
| Detached immature fruit | Cherry *Psm* R1 | 2 | 2.9 | -0.31 |
| Detached immature fruit | Cherry *Psm* R1 | 3 | 2.89 | -0.32 |
| Detached immature fruit | Cherry *Psm* R1 | 4 | 3.41 | -0.19 |
| Detached immature fruit | Cherry *Psm* R1 | 5 | 1.31 | -0.71 |
| Detached immature fruit | Cherry *Psm* R2 | 1 | 1.22 | -0.74 |
| Detached immature fruit | Cherry *Psm* R2 | 2 | 2.4 | -0.44 |
| Detached immature fruit | Cherry *Psm* R2 | 3 | 1.89 | -0.57 |
| Detached immature fruit | Cherry *Psm* R2 | 4 | 2.76 | -0.35 |
| Detached immature fruit | Cherry *Psm* R2 | 5 | 2.1 | -0.51 |
| Detached immature fruit | Cherry *Pss* | 1 | 13.49 | 2.35 |
| Detached immature fruit | Cherry *Pss* | 2 | 12.04 | 1.98 |
| Detached immature fruit | Cherry *Pss* | 3 | 10.06 | 1.48 |
| Detached immature fruit | Cherry *Pss* | 4 | 13.29 | 2.3 |
| Detached immature fruit | Cherry *Pss* | 5 | 11.88 | 1.94 |
| Detached immature fruit | Plum *Pss* | 1 | 4.89 | 0.19 |
| Detached immature fruit | Plum *Pss* | 2 | 5.31 | 0.29 |
| Detached immature fruit | Plum *Pss* | 3 | 7.21 | 0.77 |
| Detached immature fruit | Plum *Pss* | 4 | 9.02 | 1.22 |
| Detached immature fruit | Plum *Pss* | 5 | 10.45 | 1.58 |
| Detached immature fruit | RMA1 | 1 | 1.6 | -0.64 |
| Detached immature fruit | RMA1 | 2 | 1.49 | -0.67 |
| Detached immature fruit | RMA1 | 3 | 1.31 | -0.71 |
| Detached immature fruit | RMA1 | 4 | 1.36 | -0.7 |
| Detached immature fruit | RMA1 | 5 | 1.7 | -0.61 |
| Detached immature fruit | *Pph* | 1 | 1.01 | -0.79 |
| Detached immature fruit | *Pph* | 2 | 1.55 | -0.65 |
| Detached immature fruit | *Pph* | 3 | 1.36 | -0.7 |
| Detached immature fruit | *Pph* | 4 | 1.23 | -0.73 |
| Detached immature fruit | *Pph* | 5 | 1.37 | -0.7 |
| Detached leaf population count | *Pph* | 1 | 2.00E+06 | -0.92 |
| Detached leaf population count | *Pph* | 2 | 1700000 | -0.93 |
| Detached leaf population count | *Pph* | 3 | 2100000 | -0.92 |
| Detached leaf population count | *Pph* | 4 | 110000 | -0.94 |
| Detached leaf population count | *Pph* | 5 | 1.00E+05 | -0.94 |
| Detached leaf population count | *Pph* | 6 | 160000 | -0.94 |
| Detached leaf population count | *Pph* | 7 | 150000 | -0.94 |
| Detached leaf population count | *Pph* | 8 | 170000 | -0.94 |
| Detached leaf population count | *Pph* | 9 | 210000 | -0.94 |
| Detached leaf population count | Cherry *Pss* | 1 | 2.10E+08 | 1.46 |
| Detached leaf population count | Cherry *Pss* | 2 | 3.00E+08 | 2.49 |
| Detached leaf population count | Cherry *Pss* | 3 | 2.50E+08 | 1.91 |
| Detached leaf population count | Cherry *Pss* | 4 | 1.30E+08 | 0.54 |
| Detached leaf population count | Cherry *Pss* | 5 | 1.00E+08 | 0.2 |
| Detached leaf population count | Cherry *Pss* | 6 | 1.00E+08 | 0.2 |
| Detached leaf population count | Cherry *Pss* | 7 | 5.00E+07 | -0.37 |
| Detached leaf population count | Cherry *Pss* | 8 | 7.00E+07 | -0.15 |
| Detached leaf population count | Cherry *Pss* | 9 | 9.00E+07 | 0.08 |
| Detached leaf population count | Plum *Pss* | 1 | 1.70E+08 | 1 |
| Detached leaf population count | Plum *Pss* | 2 | 1.50E+08 | 0.77 |
| Detached leaf population count | Plum *Pss* | 3 | 7.00E+07 | -0.15 |
| Detached leaf population count | Plum *Pss* | 4 | 1.80E+07 | -0.74 |
| Detached leaf population count | Plum *Pss* | 5 | 1.70E+07 | -0.75 |
| Detached leaf population count | Plum *Pss* | 6 | 2.10E+07 | -0.71 |
| Detached leaf population count | Plum *Pss* | 7 | 1.30E+08 | 0.54 |
| Detached leaf population count | Plum *Pss* | 8 | 1.50E+08 | 0.77 |
| Detached leaf population count | Plum *Pss* | 9 | 1.70E+08 | 1 |
| Detached leaf population count | Cherry *Psm* R1 | 1 | 1.80E+08 | 1.11 |
| Detached leaf population count | Cherry *Psm* R1 | 2 | 2.10E+08 | 1.46 |
| Detached leaf population count | Cherry *Psm* R1 | 3 | 2.10E+08 | 1.46 |
| Detached leaf population count | Cherry *Psm* R1 | 4 | 1.50E+08 | 0.77 |
| Detached leaf population count | Cherry *Psm* R1 | 5 | 2.20E+08 | 1.57 |
| Detached leaf population count | Cherry *Psm* R1 | 6 | 1.80E+08 | 1.11 |
| Detached leaf population count | Cherry *Psm* R1 | 7 | 1.70E+08 | 1 |
| Detached leaf population count | Cherry *Psm* R1 | 8 | 1.60E+08 | 0.88 |
| Detached leaf population count | Cherry *Psm* R1 | 9 | 1.20E+08 | 0.43 |
| Detached leaf population count | Plum *Psm* R1 | 1 | 1.90E+07 | -0.73 |
| Detached leaf population count | Plum *Psm* R1 | 2 | 2.20E+07 | -0.69 |
| Detached leaf population count | Plum *Psm* R1 | 3 | 2.20E+07 | -0.69 |
| Detached leaf population count | Plum *Psm* R1 | 4 | 1500000 | -0.93 |
| Detached leaf population count | Plum *Psm* R1 | 5 | 1800000 | -0.93 |
| Detached leaf population count | Plum *Psm* R1 | 6 | 1600000 | -0.93 |
| Detached leaf population count | Plum *Psm* R1 | 7 | 2100000 | -0.92 |
| Detached leaf population count | Plum *Psm* R1 | 8 | 2400000 | -0.92 |
| Detached leaf population count | Plum *Psm* R1 | 9 | 3100000 | -0.91 |
| Detached leaf population count | Cherry *Psm* R2 | 1 | 1.80E+08 | 1.11 |
| Detached leaf population count | Cherry *Psm* R2 | 2 | 2.40E+08 | 1.8 |
| Detached leaf population count | Cherry *Psm* R2 | 3 | 2.70E+08 | 2.14 |
| Detached leaf population count | Cherry *Psm* R2 | 4 | 1.50E+08 | 0.77 |
| Detached leaf population count | Cherry *Psm* R2 | 5 | 1.20E+08 | 0.43 |
| Detached leaf population count | Cherry *Psm* R2 | 6 | 1.20E+08 | 0.43 |
| Detached leaf population count | Cherry *Psm* R2 | 7 | 1.00E+08 | 0.2 |
| Detached leaf population count | Cherry *Psm* R2 | 8 | 8.00E+07 | -0.03 |
| Detached leaf population count | Cherry *Psm* R2 | 9 | 7.00E+07 | -0.15 |
| Detached leaf population count | RMA1 | 1 | 15000 | -0.95 |
| Detached leaf population count | RMA1 | 2 | 9000 | -0.95 |
| Detached leaf population count | RMA1 | 3 | 1000 | -0.95 |
| Detached leaf population count | RMA1 | 4 | 11000 | -0.95 |
| Detached leaf population count | RMA1 | 5 | 15000 | -0.95 |
| Detached leaf population count | RMA1 | 6 | 12000 | -0.95 |
| Detached leaf population count | RMA1 | 7 | 130000 | -0.94 |
| Detached leaf population count | RMA1 | 8 | 130000 | -0.94 |
| Detached leaf population count | RMA1 | 9 | 170000 | -0.94 |
| Field tree leaf scar inoculation | Cherry *Psm* R1 | 1 | 7.4 | -0.03 |
| Field tree leaf scar inoculation | Cherry *Psm* R1 | 2 | 1 | -0.29 |
| Field tree leaf scar inoculation | Cherry *Psm* R1 | 3 | 3.5 | -0.19 |
| Field tree leaf scar inoculation | Cherry *Psm* R1 | 4 | 6.1 | -0.09 |
| Field tree leaf scar inoculation | Cherry *Psm* R1 | 5 | 174 | 6.79 |
| Field tree leaf scar inoculation | Cherry *Psm* R1 | 6 | NA | NA |
| Field tree leaf scar inoculation | Cherry *Psm* R1 | 7 | 5.4 | -0.11 |
| Field tree leaf scar inoculation | Cherry *Psm* R1 | 8 | 4.6 | -0.15 |
| Field tree leaf scar inoculation | Cherry *Psm* R1 | 9 | 7.5 | -0.03 |
| Field tree leaf scar inoculation | Cherry *Psm* R1 | 10 | 0 | -0.33 |
| Field tree leaf scar inoculation | Plum *Psm* R1 | 1 | 3.6 | -0.19 |
| Field tree leaf scar inoculation | Plum *Psm* R1 | 2 | 0 | -0.33 |
| Field tree leaf scar inoculation | Plum *Psm* R1 | 3 | 0 | -0.33 |
| Field tree leaf scar inoculation | Plum *Psm* R1 | 4 | 2.1 | -0.25 |
| Field tree leaf scar inoculation | Plum *Psm* R1 | 5 | 0 | -0.33 |
| Field tree leaf scar inoculation | Plum *Psm* R1 | 6 | 0 | -0.33 |
| Field tree leaf scar inoculation | Plum *Psm* R1 | 7 | 0 | -0.33 |
| Field tree leaf scar inoculation | Plum *Psm* R1 | 8 | 0 | -0.33 |
| Field tree leaf scar inoculation | Plum *Psm* R1 | 9 | 0 | -0.33 |
| Field tree leaf scar inoculation | Plum *Psm* R1 | 10 | 0 | -0.33 |
| Field tree leaf scar inoculation | Cherry *Pss* | 1 | 2.4 | -0.24 |
| Field tree leaf scar inoculation | Cherry *Pss* | 2 | 1.5 | -0.27 |
| Field tree leaf scar inoculation | Cherry *Pss* | 3 | 29.2 | 0.86 |
| Field tree leaf scar inoculation | Cherry *Pss* | 4 | NA | NA |
| Field tree leaf scar inoculation | Cherry *Pss* | 5 | 33.5 | 1.04 |
| Field tree leaf scar inoculation | Cherry *Pss* | 6 | NA | NA |
| Field tree leaf scar inoculation | Cherry *Pss* | 7 | 4.2 | -0.16 |
| Field tree leaf scar inoculation | Cherry *Pss* | 8 | 9.2 | 0.04 |
| Field tree leaf scar inoculation | Cherry *Pss* | 9 | 1.2 | -0.29 |
| Field tree leaf scar inoculation | Cherry *Pss* | 10 | 32.7 | 1 |
| Field tree leaf scar inoculation | Plum *Pss* | 1 | 2.4 | -0.24 |
| Field tree leaf scar inoculation | Plum *Pss* | 2 | 2.5 | -0.23 |
| Field tree leaf scar inoculation | Plum *Pss* | 3 | 0 | -0.33 |
| Field tree leaf scar inoculation | Plum *Pss* | 4 | NA | NA |
| Field tree leaf scar inoculation | Plum *Pss* | 5 | 0 | -0.33 |
| Field tree leaf scar inoculation | Plum *Pss* | 6 | NA | NA |
| Field tree leaf scar inoculation | Plum *Pss* | 7 | 5.7 | -0.1 |
| Field tree leaf scar inoculation | Plum *Pss* | 8 | 0 | -0.33 |
| Field tree leaf scar inoculation | Plum *Pss* | 9 | 0 | -0.33 |
| Field tree leaf scar inoculation | Plum *Pss* | 10 | 11.5 | 0.14 |
| Field tree leaf scar inoculation | Cherry *Psm* R2 | 1 | 2.1 | -0.25 |
| Field tree leaf scar inoculation | Cherry *Psm* R2 | 2 | 9.1 | 0.04 |
| Field tree leaf scar inoculation | Cherry *Psm* R2 | 3 | 0 | -0.33 |
| Field tree leaf scar inoculation | Cherry *Psm* R2 | 4 | 0 | -0.33 |
| Field tree leaf scar inoculation | Cherry *Psm* R2 | 5 | 2.1 | -0.25 |
| Field tree leaf scar inoculation | Cherry *Psm* R2 | 6 | NA | NA |
| Field tree leaf scar inoculation | Cherry *Psm* R2 | 7 | 3.2 | -0.2 |
| Field tree leaf scar inoculation | Cherry *Psm* R2 | 8 | 0 | -0.33 |
| Field tree leaf scar inoculation | Cherry *Psm* R2 | 9 | 10 | 0.07 |
| Field tree leaf scar inoculation | Cherry *Psm* R2 | 10 | 35 | 1.1 |
| Field tree leaf scar inoculation | *Pph* | 1 | 56.5 | 1.98 |
| Field tree leaf scar inoculation | *Pph* | 2 | 0 | -0.33 |
| Field tree leaf scar inoculation | *Pph* | 3 | NA | NA |
| Field tree leaf scar inoculation | *Pph* | 4 | 0 | -0.33 |
| Field tree leaf scar inoculation | *Pph* | 5 | 2.7 | -0.22 |
| Field tree leaf scar inoculation | *Pph* | 6 | 0 | -0.33 |
| Field tree leaf scar inoculation | *Pph* | 7 | 0 | -0.33 |
| Field tree leaf scar inoculation | *Pph* | 8 | NA | NA |
| Field tree leaf scar inoculation | *Pph* | 9 | 0 | -0.33 |
| Field tree leaf scar inoculation | *Pph* | 10 | 0 | -0.33 |
| Field tree leaf scar inoculation | RMA1 | 1 | 0 | -0.33 |
| Field tree leaf scar inoculation | RMA1 | 2 | 8.9 | 0.03 |
| Field tree leaf scar inoculation | RMA1 | 3 | NA | NA |
| Field tree leaf scar inoculation | RMA1 | 4 | NA | NA |
| Field tree leaf scar inoculation | RMA1 | 5 | 0.4 | -0.32 |
| Field tree leaf scar inoculation | RMA1 | 6 | 1.4 | -0.28 |
| Field tree leaf scar inoculation | RMA1 | 7 | 0 | -0.33 |
| Field tree leaf scar inoculation | RMA1 | 8 | NA | NA |
| Field tree leaf scar inoculation | RMA1 | 9 | 0 | -0.33 |
| Field tree leaf scar inoculation | RMA1 | 10 | 0 | -0.33 |
| Field tree wound inoculation | Cherry *Psm* R1 | 1 | 15.5 | -0.43 |
| Field tree wound inoculation | Cherry *Psm* R1 | 2 | 32.6 | -0.21 |
| Field tree wound inoculation | Cherry *Psm* R1 | 3 | NA | NA |
| Field tree wound inoculation | Cherry *Psm* R1 | 4 | 301 | 3.14 |
| Field tree wound inoculation | Cherry *Psm* R1 | 5 | 360.5 | 3.88 |
| Field tree wound inoculation | Cherry *Psm* R1 | 6 | 34 | -0.19 |
| Field tree wound inoculation | Cherry *Psm* R1 | 7 | 14.5 | -0.44 |
| Field tree wound inoculation | Cherry *Psm* R1 | 8 | 100 | 0.63 |
| Field tree wound inoculation | Cherry *Psm* R1 | 9 | 34.7 | -0.19 |
| Field tree wound inoculation | Cherry *Psm* R1 | 10 | 307 | 3.21 |
| Field tree wound inoculation | Plum *Psm* R1 | 1 | NA | NA |
| Field tree wound inoculation | Plum *Psm* R1 | 2 | 14 | -0.44 |
| Field tree wound inoculation | Plum *Psm* R1 | 3 | 17.4 | -0.4 |
| Field tree wound inoculation | Plum *Psm* R1 | 4 | 20 | -0.37 |
| Field tree wound inoculation | Plum *Psm* R1 | 5 | 16.3 | -0.42 |
| Field tree wound inoculation | Plum *Psm* R1 | 6 | 14.5 | -0.44 |
| Field tree wound inoculation | Plum *Psm* R1 | 7 | 13.4 | -0.45 |
| Field tree wound inoculation | Plum *Psm* R1 | 8 | 13.1 | -0.46 |
| Field tree wound inoculation | Plum *Psm* R1 | 9 | 13.3 | -0.45 |
| Field tree wound inoculation | Plum *Psm* R1 | 10 | 5.7 | -0.55 |
| Field tree wound inoculation | Cherry *Pss* | 1 | 10.6 | -0.49 |
| Field tree wound inoculation | Cherry *Pss* | 2 | 25.1 | -0.31 |
| Field tree wound inoculation | Cherry *Pss* | 3 | NA | NA |
| Field tree wound inoculation | Cherry *Pss* | 4 | 15.2 | -0.43 |
| Field tree wound inoculation | Cherry *Pss* | 5 | 111.5 | 0.77 |
| Field tree wound inoculation | Cherry *Pss* | 6 | 26.2 | -0.29 |
| Field tree wound inoculation | Cherry *Pss* | 7 | 33.7 | -0.2 |
| Field tree wound inoculation | Cherry *Pss* | 8 | 147 | 1.22 |
| Field tree wound inoculation | Cherry *Pss* | 9 | 210 | 2 |
| Field tree wound inoculation | Cherry *Pss* | 10 | 90 | 0.5 |
| Field tree wound inoculation | Plum *Pss* | 1 | 8.2 | -0.52 |
| Field tree wound inoculation | Plum *Pss* | 2 | 17.5 | -0.4 |
| Field tree wound inoculation | Plum *Pss* | 3 | NA | NA |
| Field tree wound inoculation | Plum *Pss* | 4 | 24.5 | -0.31 |
| Field tree wound inoculation | Plum *Pss* | 5 | NA | NA |
| Field tree wound inoculation | Plum *Pss* | 6 | 24.3 | -0.32 |
| Field tree wound inoculation | Plum *Pss* | 7 | 33.1 | -0.21 |
| Field tree wound inoculation | Plum *Pss* | 8 | 19.2 | -0.38 |
| Field tree wound inoculation | Plum *Pss* | 9 | 271 | 2.76 |
| Field tree wound inoculation | Plum *Pss* | 10 | 41 | -0.11 |
| Field tree wound inoculation | Cherry *Psm* R2 | 1 | NA | NA |
| Field tree wound inoculation | Cherry *Psm* R2 | 2 | 20 | -0.37 |
| Field tree wound inoculation | Cherry *Psm* R2 | 3 | 39.2 | -0.13 |
| Field tree wound inoculation | Cherry *Psm* R2 | 4 | 24.4 | -0.31 |
| Field tree wound inoculation | Cherry *Psm* R2 | 5 | NA | NA |
| Field tree wound inoculation | Cherry *Psm* R2 | 6 | 10.2 | -0.49 |
| Field tree wound inoculation | Cherry *Psm* R2 | 7 | 27.4 | -0.28 |
| Field tree wound inoculation | Cherry *Psm* R2 | 8 | 32 | -0.22 |
| Field tree wound inoculation | Cherry *Psm* R2 | 9 | 20 | -0.37 |
| Field tree wound inoculation | Cherry *Psm* R2 | 10 | 9.5 | -0.5 |
| Field tree wound inoculation | *Pph* | 1 | 12.6 | -0.46 |
| Field tree wound inoculation | *Pph* | 2 | 31 | -0.23 |
| Field tree wound inoculation | *Pph* | 3 | NA | NA |
| Field tree wound inoculation | *Pph* | 4 | 14.4 | -0.44 |
| Field tree wound inoculation | *Pph* | 5 | NA | NA |
| Field tree wound inoculation | *Pph* | 6 | 10.4 | -0.49 |
| Field tree wound inoculation | *Pph* | 7 | 15.2 | -0.43 |
| Field tree wound inoculation | *Pph* | 8 | 8.9 | -0.51 |
| Field tree wound inoculation | *Pph* | 9 | 11.2 | -0.48 |
| Field tree wound inoculation | *Pph* | 10 | 44 | -0.07 |
| Field tree wound inoculation | RMA1 | 1 | NA | NA |
| Field tree wound inoculation | RMA1 | 2 | 24.2 | -0.32 |
| Field tree wound inoculation | RMA1 | 3 | NA | NA |
| Field tree wound inoculation | RMA1 | 4 | 27 | -0.28 |
| Field tree wound inoculation | RMA1 | 5 | 11.4 | -0.48 |
| Field tree wound inoculation | RMA1 | 6 | 14.3 | -0.44 |
| Field tree wound inoculation | RMA1 | 7 | 12.1 | -0.47 |
| Field tree wound inoculation | RMA1 | 8 | 13.4 | -0.45 |
| Field tree wound inoculation | RMA1 | 9 | NA | NA |
| Field tree wound inoculation | RMA1 | 10 | 9 | -0.51 |

**Table S28: Data used in correlation analysis for Figure 10.** This is the standardised mean disease for a set of seven bacterial strains on cherry cv. Van using different inoculation methods
