# Supplementary material for: Characterization of the pathogenicity of strains of Pseudomonas syringae towards cherry and plum
Source: Plant Pathol. 2018 Feb 14;67(5):1177–93. doi: 10.1111/ppa.12834 (PMC5993217; doi:10.1111/ppa.12834)
Supplement: Supplementary file 37 — Table S29. Correlation coefficients calculated for each different inoculation experiment correlated with the symptom length results of the field tree wound inoculation experiment. [file PPA-67-1177-s037.docx]

| **Inoculation type** | **Correlation with field tree wound inoculation** |
| --- | --- |
| Field tree leaf scar inoculation | 0.9 |
| Cut shoot inoculation | 0.76 |
| Detached immature fruit | 0.37 |
| Detached leaf population count | 0.77 |

**Table S29: Correlation coefficients calculated for each different inoculation experiment correlated with the symptom length results of the field tree wound inoculation experiment**
