# Supplementary material for: Characterization of the pathogenicity of strains of Pseudomonas syringae towards cherry and plum
Source: Plant Pathol. 2018 Feb 14;67(5):1177–93. doi: 10.1111/ppa.12834 (PMC5993217; doi:10.1111/ppa.12834)
Supplement: Supplementary file 38 — Text S1. Supplementary methods. [file PPA-67-1177-s038.docx]

**SUPPLEMENTARY METHODS**

**Statistical analysis**

*Glasshouse whole tree inoculation*

The glasshouse whole-tree experiment produced ordinal categorical data (disease scores) and was analysed using the Proportional Odds Model (POM) of the R package ordinal (Christensen, 2015). This involved fitting a cumulative logit model: clm(score ~ strain). As there was too many strains with the same results, strains were collapsed into groups with similar disease score profiles and a subsequent simplified model compared to the initial model to check it was not significantly different.

*Field inoculations*

POM was used to analyse the field disease score data. Wound and scar data could be analysed together due to similar variance. For cherry variances: scar: 1.01 and wound: 1.43. For plum variances: scar: 0.49 and wound: 0.86. This was performed as in the glasshouse whole tree inoculation, fitting each model with additional factors and using ANOVA to determine if these factors affected the model. The Final model was clm(score ~ strain + cv + ino + block).

For the symptom length data obtained from the field experiment, a REML (Restricted Maximum Likelihood) analysis was used to account for missing values (18% cherry trees and 26% of plum trees were dead at time of assessment). As leaf scar and wound results had very different variances (1258 and 1884 for cherry, 331 and 132 for plum, leaf scar and wound respectively), they were analysed separately. Strain and cultivar were fixed effects, and block (with tree number nested within) was treated as a random effect. The model: lmer(log_2_(length+1) ~ cultivar * strain + (1|block/no.).

*Detached cut shoot inoculations*

The cut shoot test was analysed using an ANOVA on the log_2_-transformed data set. The model: aov(log_2_(area+1) ~ strain * host/cv + block ). Plum and cherry were analysed in the same model as the variances were not dissimilar (cherry: 221 and plum: 280).

*Immature cherry fruit inoculation*

For the cherry fruit experiment comparing all *P. syringae* strains, an ANOVA was used: aov(log_2_(length+1) ~ strain + replicate). For the cherry fruit experiment comparing cherry cultivars, a REML analysis was used due to missing values (19% of fruit were contaminated so were removed from the analysis). The model: lmer(log_2_(length) ~ cultivar * strain + (1|block).

*Detached leaf population counts and symptom score analysis*

To analyse individual leaf population experiments, ANOVA was used when the design was balanced. The model: aov(log_2_(cfu) ~ strain + experiment/leaf/replicate). Where the design was unbalanced (where number of replicates for strains was different) REML was utilised as it is robust to missing values. To analyse the symptom development during the time period indicative of a hypersensitive response on leaves, the Area Under the Disease Progression Curve (AUDPC) was calculated (de Mendiburu, 2016) between 0-48 hours. An ANOVA used to determine differences between strains. The model: aov(AUDPC~ strain + experiment/leaf).

**Electron microscopy**

Electron microscopy was performed by Dr Ian Brown (University of Kent) on infected cherry leaves. Detached leaves were infiltrated with bacteria at 2x10^6^ CFU/ml and incubated for one week at 22 °C. Microscopy was then performed on inoculation sites as previously described (Soylu *et al*., 2005).

**Post-hoc analyses**

For all analyses, a post-hoc Tukey-HSD (honest significant difference) test (p=0.05), available in the agricolae package of R (de Mendiburu, 2016), was used to group similar strains. Alternatively, for linear models, the lsmeans package (Lenth, 2016) was used to extract means and the cld function of the multcomp package (Hothorn *et al*., 2008) used to obtain groupings.

**Correlation of different pathogenicity tests**

To compare different inoculation methods, data for a set of strains used in all experiments (cherry Psm R1-5244, plum Psm R1-5300, cherry Psm R2-5255/R2-leaf, cherry Pss-9097, plum Pss-9293, non-host *Aquilegia vulgaris* RMA1 and non-host *Phaseolus vulgaris* *Pph*). *Psm* R2-5255 was used in the cut shoot assay, whilst *Psm* R2-leaf was used in all other assays (Note that these strains exhibited identical pathogenicity profiles in the glasshouse wound assay). The different datasets included the leaf scar symptom length, cut shoot percentage area of necrosis, immature cherry fruit symptom length, bacterial population counts in CFU/ml. Means were extracted and standardised (mean/sd). These standardised means were used in correlation analysis (Pearson’s correlation coefficient) with field wound symptom length. The package ggplot2 was used to make graphs of raw data (Warnes *et al*., 2016).

**Supplementary references**

Almeida NF, Yan S, Lindeberg M *et al.*, 2009. A draft genome sequence of *Pseudomonas syringae* pv. *tomato* T1 reveals a type III effector repertoire significantly divergent from that of *Pseudomonas syringae* pv. *tomato* DC3000. *Molecular plant-microbe interactions : MPMI* **22**, 52–62.

Baltrus DA, Nishimura MT, Romanchuk A *et al.*, 2011. Dynamic evolution of pathogenicity revealed by sequencing and comparative genomics of 19 *Pseudomonas syringae* isolates. *PLoS Pathogens* **7**, 22.

Baltrus DA, Yourstone S, Lind A *et al.*, 2014a. Draft genome sequences of a phylogenetically diverse suite of *Pseudomonas syringae* strains from multiple source populations. **2**, 1–2.

Baltrus DA, Dougherty K, Beckstrom-Sternberg SM, Beckstrom-Sternberg JS, Foster JT, 2014b. Incongruence between multi-locus sequence analysis (MLSA) and whole-genome-based phylogenies: *Pseudomonas syringae* pathovar *pisi* as a cautionary tale. *Molecular plant pathology*, **15**, 461-465.

Bartoli C, Carrere S, Lamichhane R, Varvaro L, Morris CE, 2015. Whole-genome sequencing of 10 *Pseudomonas syringae* strains representing different host range spectra. *Genome Announcements* **3**, 2–3.

Buell CR, Joardar V, Lindeberg M *et al.*, 2003. The complete genome sequence of the Arabidopsis and tomato pathogen *Pseudomonas syringae* pv. *tomato* DC3000. *Proceedings of the National Academy of Sciences of the United States of America* **100**, 10181–10186.

Dudnik A, Dudler R, 2013. High-quality draft genome sequence of *Pseudomonas syringae* pv. *syringae* strain SM, Isolated from Wheat. *Genome Announcements*, **1**, 6–7.

Feil H, Feil WS, Chain P *et al.*, 2005. Comparison of the complete genome sequences of P*seudomonas syringa*e pv. *syringae* B728a and pv. *tomato* DC3000. *Proceedings of the National Academy of Sciences of the United States of America* **102**, 11064–11069.

Green S, Studholme DJ, Laue BE *et al.*, 2010. Comparative genome analysis provides insights into the evolution and adaptation of *Pseudomonas syringae* pv. *aesculi* on *Aesculus hippocastanum*. *PloS one* **5**, e10224.

Guttman DS, Vinatzer B a, Sarkar SF, Ranall M V, Kettler G, Greenberg JT, 2002. A functional screen for the type III (Hrp) secretome of the plant pathogen *Pseudomonas* *syringae*. *Science.* **295**, 1722–1726.

Hockett KL, Nishimura MT, Karlsrud E, Dougherty K, Baltrus D a, 2014. *Pseudomonas syringae* CC1557: A highly virulent strain with an unusually small type III effector repertoire that includes a novel effector. *Molecular plant-microbe interactions : MPMI* **27**, 923–932.

Liu H, Qiu H, Zhao W *et al.*, 2012. Genome sequence of the plant pathogen *Pseudomonas syringae* pv. *panici* LMG 2367. *Journal of Bacteriology* **194**, 5693–5694.

Mazzaglia A, Studholme DJ, Taratufolo MC *et al.*, 2012. *Pseudomonas syringae* pv. *actinidiae* (PSA) isolates from recent bacterial canker of kiwifruit outbreaks belong to the same genetic lineage. *PLoS ONE* **7**, 1–11.

Mott GA, Thakur S, Smakowska E *et al.*, 2016. Genomic screens identify a new phytobacterial microbe-associated molecular pattern and the cognate *Arabidopsis* receptor-like kinase that mediates its immune elicitation. *Genome Biology* **17**, 98.

Mucyn TS, Yourstone S, Lind AL *et al.*, 2014. Variable suites of non-effector genes are co-regulated in the type III secretion virulence regulon across the *Pseudomonas syringae* phylogeny. *PLoS pathogens* **10**, e1003807.

Nowell RW, Laue BE, Sharp PM, Green S, 2016. Comparative genomics reveals genes significantly associated with woody hosts in the plant pathogen *Pseudomonas syringae*. *Molecular Plant Pathology*, 1–16.

O’Brien HE, Thakur S, Gong Y *et al.*, 2012. Extensive remodeling of the *Pseudomonas syringae* pv. *avellanae* type III secretome associated with two independent host shifts onto hazelnut. *BMC microbiology* **12**, 141.

Qi M, Wang D, Bradley CA, Zhao Y, 2011. Genome sequence analyses of *Pseudomonas savastanoi* pv. *glycinea* and subtractive hybridization-based comparative genomics with nine Pseudomonads. *PLoS ONE* **6**, e16451.

Ravindran A, Jalan N, Yuan JS, Wang N, Gross DC, 2015. Comparative genomics of *Pseudomonas syringae* pv. *syringae* strains B301D and HS191 and insights into intrapathovar traits associated with plant pathogenesis. *MicrobiologyOpen* **4**, 553–573.

Rodríguez-Palenzuela P, Matas IM, Murillo J *et al.*, 2010. Annotation and overview of the *Pseudomonas savastanoi* pv. *savastanoi* NCPPB 3335 draft genome reveals the virulence gene complement of a tumour-inducing pathogen of woody hosts. *Environmental microbiology* **12**, 1604–1620.

Thakur S, Weir BS, Guttman D, 2016. Phytopathogen genome announcement: Draft genome sequences of 62 *Pseudomonas syringae* type and pathotype strains. *Molecular Plant-Microbe Interactions* **29**, 243-246.

Zhao W, Jiang H, Tian Q, Hu J, 2015. Draft genome sequence of *Pseudomonas syringae* pv. *persicae* NCPPB 2254. **3**, 54–55.
