# Supplementary material for: Characterization of the pathogenicity of strains of Pseudomonas syringae towards cherry and plum
Source: Plant Pathol. 2018 Feb 14;67(5):1177–93. doi: 10.1111/ppa.12834 (PMC5993217; doi:10.1111/ppa.12834)
Supplement: Supplementary file 1 — Figure S1. Images of immature cherry fruits inoculated with Pseudomonas syringae pv. morsprunorum race 1 strains. Images were taken 10 dpi. Five replicate cherries were inoculated per strain. Strains from cherry are labelled in pink, whilst those from plum are in blue. [file PPA-67-1177-s001.pdf]

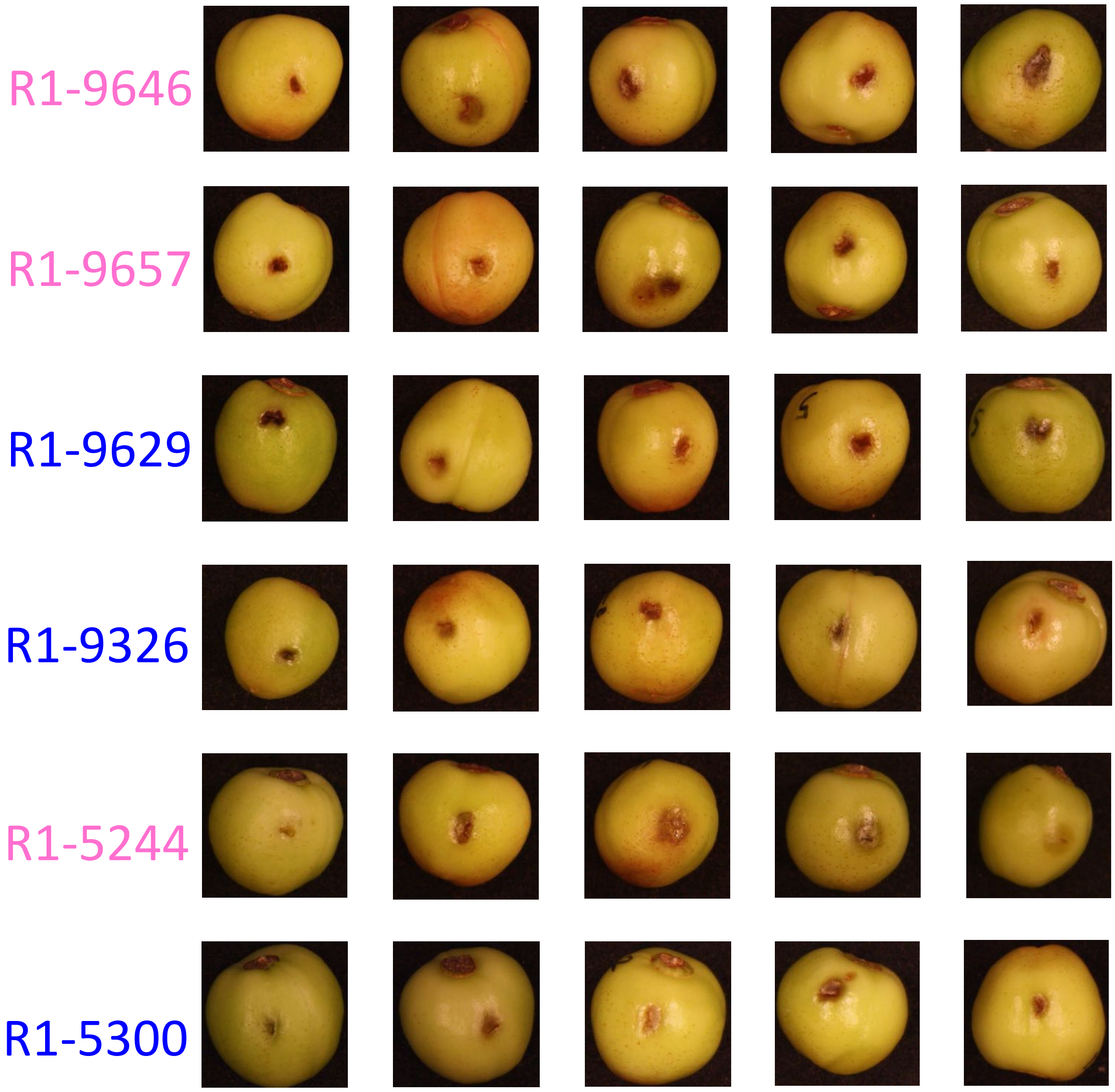
R1-9646
R1-9657
R1-9629
R1-9326
R1-5244
R1-5300
